# Description and comparison of Philippine hornbill (Bucerotidae) vocalizations

**DOI:** 10.3897/BDJ.7.e31723

**Published:** 2019-11-13

**Authors:** Shari Limbo Guerra, Juan Carlos T. Gonzalez, Emmanuel Francisco Rafael

**Affiliations:** 1 Institute of Biological Sciences, University of the Philippines Los Baños, Laguna, Philippines Institute of Biological Sciences, University of the Philippines Los Baños Laguna Philippines; 2 UPLB Museum of Natural History, Laguna, Philippines UPLB Museum of Natural History Laguna Philippines; 3 Avilon Wildlife Conservation Foundation, Pasig City, Philippines Avilon Wildlife Conservation Foundation Pasig City Philippines

**Keywords:** Species limits, hornbills, vocalisation, bioacoustics

## Abstract

The role of vocalisation for the Philippine hornbills' ecology and speciation and their implication in understanding speciation is not well understood. We described and compared recorded calls of seven hornbill taxa in captivity namely Mindanao Wrinkled hornbill (*Rhabdotorrhinus leucocephalus*), Rufous-headed hornbill (*Rhabdotorrhinus waldeni*), Luzon Rufous hornbill (*Buceros hydrocorax hydrocorax*), Samar Rufous hornbill (*Buceros hydrocorax semigaleatus*), Mindanao Rufous hornbill (*Buceros hydrocorax mindanensis*), Mindanao Tarictic hornbill (*Penelopides affinis*), Samar Tarictic hornbill (*Penelopides samarensis*), Visayan Tarictic hornbill (*Penelopides panini*) and Luzon Tarictic hornbill (*Penelopides manillae*), as well as comparison with the non-native Papuan hornbill (*Rhyticeros plicatus*). Vocalisation analysis included call duration, minimum frequency, maximum frequency, bandwidth and peak frequency. For each species in the sample, the mean and standard deviation were used to calculate the Cohen’s d statistic by using an effect size calculator. Results showed that the effect size for minimum frequency was small for *P. panini* vs. *P. samarensis* and *B. hydrocorax* vs. *B. h. mindanensis*. However, bandwidth, duration, minimum frequency, maximum frequency and peak frequency have large effect sizes for the rest of the allopatric species pairs. Hornbills' conspicuous resonating calls are sufficiently quantifiable for bioacoustic analysis and may provide new insights for their taxonomic review.

## Introduction

Hornbills (Bucerotidae) are a charismatic group of tropical birds, under the Order Bucerotiformes, recognised for their long decurved bill supported with a prominent casque. Some 60-64 species of Bucerotiformes are currently recognised worldwide, including two species of ground-hornbills within the family Bucorvidae. The majority of the species belong to the family Bucerotidae and which all share the unique trait of plastering the nest-cavity (Gonzalez 2012). Numerous studies for conservation focused on surveys and other correlated projects which include habitat re-establishment, breeding, instruction for handling and education and public awareness (Lum and Poonswad 2005).

The resounding vocalisations of hornbills aids their communication interaction with conspecifics and sympatrics in dense forest habitats, defence, territory and threat (Haimoff 2008, Poonswad et al. 2013, Policht et al. 2009), but there are few studies on their bioacoustics (Kemp 1998, Gonzalez 2012). However, bioacoustic analysis of the hornbills' calls has remained insufficiently studied despite its potential to provide valuable information to understanding communication and evolution as shown by Oba (1998). It has long been debated on the use of the casque and their implications to the resonating calls produced by hornbills (Kemp 1995, Kinnaird and O'Brien 2007). Tobias et al. (2010) proposed a standardised approach for delimiting of species and establishment of the taxonomic relationships between species and subspecies, based on multiple phenotypic characters i.e. biometrics, plumage and voice.

Studies by Gonzalez (2012) and Gonzalez et al. (2013) show that molecular phylogenetic relationships within Bucerotidae corroborate well with the vocal variations across the family. Currently, the lack of available records of hornbill calls, especially for the Philippine species, impedes further bioacoustic analyses. Therefore, the aim of our study is to record and examine the loud calls of Philippine hornbills kept in captivity in order to describe and compare hornbill vocalisations, based on the standardised criteria parameters given in the standardised criteria for species delimitation.

## Material and methods

**Study site and recording of vocalisations**

Observations took place during the day and the advertisement calls were recorded on an opportunistic basis with a Sony PBR-330 parabolic reflector, Uniso UC-0163 hands-free microphone and Sony IC recorder ICD-BX140. During the recording of the calls, the microphone was approximately 8 to 10 metres away from the hornbill species in captivity. The male hornbills, used in the study, were inside the cages with the other hornbills of the same species. The majority of the vocal sampling was derived from captive hornbills due to the limitations of recording vocalisations of Philippine hornbills in the wild. For comparison, analysis was supplemented by recordings available from online databases.

**Figure 1.**
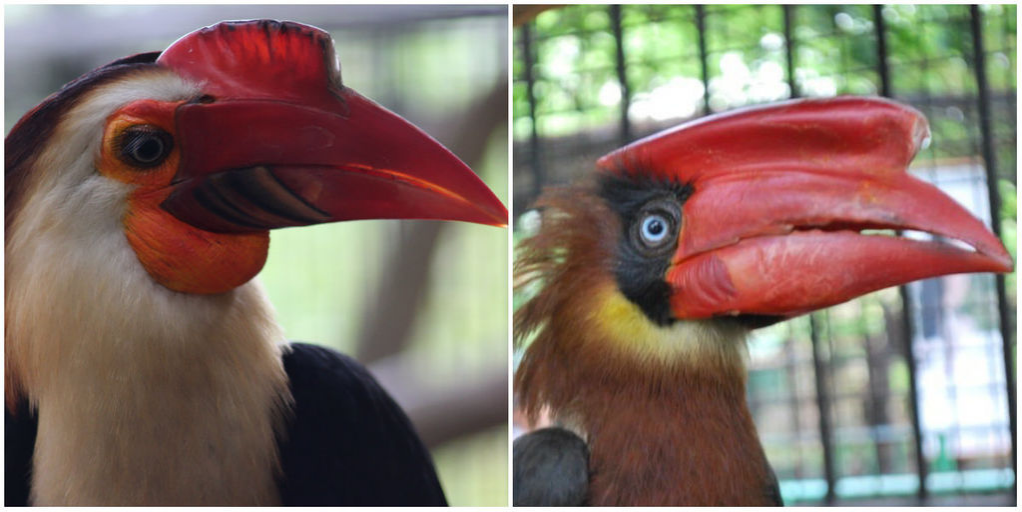
Male captive Philippine hornbill species. *Rhabdotorrhinus leucocephalus* (left) and *Buceros hydrocorax* (right).

Adult male captive hornbills in Avilon Zoo, Rodriguez, Rizal were used in this study (Fig. 1). These include the Mindanao Wrinkled hornbill (*Rhabdotorrhinus leucocephalus* (Vieillot, 1816), Luzon Rufous hornbill (*Buceros hydrocorax hydrocorax* Linnaeus, 1766), Visayan Rufous hornbill (*Buceros hydrocorax semigaleatus* Tweeddale, 1878), Mindanao Rufous hornbill (*Buceros hydrocorax mindanensis* Tweeddale, 1877), Luzon Tarictic hornbill (*Penelopides manillae* (Boddaert, 1783)) and Visayan Tarictic hornbill (*Penelopides panini* (Boddaert, 1783)). The Papuan hornbill (*Rhyticeros plicatus* (J.R. Forster, 1781)) was also incorporated in the study since it was formerly a subspecies of *R. leucocephalus*. For added comparison, the vocalisations of the Rufous-headed hornbill (*Rhabdotorrhinus waldeni* (Sharpe, 1877), Mindanao hornbill (*Penelopides affinis* Tweeddale, 1877) and Samar hornbill (*Penelopides samarensis* Steere, 1890) were obtained from Policht et al. (2009), Xeno-canto and Avocet, respectively.

**Analysis**

Each individual had six to ten replicates and the non-overlapping vocalisations with the lowest background noise were used for analysis. The editing and noise reduction tools were utilised in Audacity 2.1.3 to eliminate unnecessary noise. Recordings were digitised and analysed using waveforms and spectrograms generated by Raven Pro 1.2 software. The vocalisations were quantified following the criteria proposed by Tobias et al. (2010) which include duration, maximum frequency, minimum frequency, bandwidth and peak frequency. The software automatically generated values for the said parameters.

For each individual in the sample, the individual average for each parameter was determined. Thereafter, the mean and standard deviation were used to calculate the effect size index (Becker 2000) by using an excel effect size calculator. Cohen’s *d* is beneficial in raw units that are regarded random upon manifestation in units of variability. An effect size of 0.2, 0.5 and 0.8 is an indication of small, medium and large effect sizes, respectively (Cohen 1988). The variables with the strongest temporal (s) and strongest spectral (kHz) characters were used in computing for the total score (Table 1). There are four degrees of magnitude, namely minor (1), medium (2), major (3) and exceptional (4) differences. A threshold of 7 served as the basis for species delimitation amongst the taxa.

## Results and Discussion

Vocalisations of hornbills were found to reveal information on the individual (Policht et al. 2009). Tarictic hornbills are the smallest amongst the hornbills and they have narrow casques (Kennedy et al. 2000, Policht et al. 2009). They also emit relatively higher frequencies compared to those of *Buceros, Rhyticeros* and *Rhabdotorrhiunus*. This is due to the association of casque resonance frequency to the fundamental frequency (Alexander et al. 1994, Policht et al. 2009).

For this study, there were six individuals for *P. affinis*, five for *P. manillae* and four for *P. panini* and *P. samarensis* (Table 2). Results showed that *P. manillae* vs. *P. samarensis* had the greatest values of Cohen’s *d* in bandwidth (11.82), duration (6.55) and minimum frequency (6.66). On the other hand, *P. manillae* vs. *P. panini* obtained the highest value in minimum frequency (6.66) and maximum frequency (7.82), while *P. affinis* vs. *P. manillae* had the greatest value in peak frequency (9.88). Collectively, the calls of all members of the genus *Penelopides* are described as a high-pitched trumpeting bleat, but there are noticeable differences between them, which can be differentiated further, based on their quantified calls.

The effect sizes for *P. affinis* vs. *P. panini* were large for all vocal characteristics- bandwidth (4.93), duration (2.63), minimum frequency (1.12), maximum frequency (6.06) and peak frequency (4.57). A score of 5 was given to the species pair due to obtaining medium (2) and major (3) scores for the strongest temporal and strongest spectral characters, respectively.

On the other hand, *P. affinis* vs. *P. manillae* also generated a large effect size for bandwidth (2.99), duration (6.10), minimum frequency (4.60), maximum frequency (1.26) and peak frequency (9.88). The strongest temporal and strongest spectral character resulted in a score of 6. Thus, the large effect sizes amongst the species in *Penelopides* strongly demonstrated variation in vocalisation amongst the taxon.

The magnitude of the strongest temporal and strongest spectral characters were 2 and 3, respectively. In total, a score of 5 was given to *P. affinis* and *P. samarensis* due to obtaining large effect sizes for all vocal characteristics – bandwidth (3.02), duration (3.18), minimum frequency (1.00), maximum frequency (6.25) and peak frequency (7.16) acquired large effect sizes.

Between *P. manillae* vs. *P. samarensis*, the effect size was also large for all variables, earning a score of 7 – bandwidth (11.82), duration (6.55), minimum frequency (6.66), maximum frequency (4.91) and peak frequency (4.90). The major and exceptional values of duration and bandwidth resulted in a score of 7.

However, *P. panini* vs. *P. samarensis* had a small effect size on minimum frequency (0.26), but the other variables, bandwidth (17.53), duration (4.54), maximum frequency (23.51) and peak frequency (4.50), had large effect sizes. A score of 6 was given to the species pair upon acquiring medium and exceptional values.

Lastly, *P. manillae* vs. *P. panini* received a score of 6 because of having large effect sizes for bandwidth (3.99), duration (5.39), minimum frequency (6.66), maximum frequency (7.82) and peak frequency (7.46). Both of the greatest temporal and spectral characters gained major scores for the total cumulative score.

There were five individuals for *R. leucocephalus*, three for *R. plicatus* and one for *R. waldeni Table 3*. It can be inferred that the pair of *R. waldeni* vs. *R. plicatus* had the highest proportions in bandwidth, minimum frequency, maximum frequency and peak frequency. As for duration, *R. leucocephalus* vs. *R. waldeni* had the greatest proportion compared to the rest.

Between *R. leucocephalus* vs. *R. waldeni*, bandwidth (5.31), duration (7.14), minimum frequency (3.00), maximum frequency (3.44) and peak frequency (4.63) have large effect sizes. The strongest temporal and spectral characters attained a medium score which resulted in a total score of 6. As seen in Table 5, this certifies the recent split due to addition of acoustic data to the parallel results of morphological and genetic data (Gonzalez et al. 2013).

The species-pairs of *R. leucocephalus* vs. *R. plicatus* and *R. waldeni* vs. *R. plicatus* both obtained a score of 7 due to having large effect sizes for bandwidth (7.59 and 9.17), duration (5.99 and 5.60), minimum frequency (15.38 and 18.78), maximum frequency (11.15 and 11.84) and peak frequency (15.15 and 16.43), respectively. Major and exceptional scores were given to the greatest spectral and temporal characters (Table 5). In comparison, hornbills from the genus *Rhabdotorrhinus* tend to have a more staccato bark over the harsher bark notable from hornbills of the genus *Rhyticeros* (Gonzalez et al. 2013).

The species pair of *B. h. semigaleatus* and *B. h. mindanensis* had the highest Cohen’s *d* for bandwidth, minimum frequency, maximum frequency and peak frequency while *B. h. hydrocorax* vs. *B. h. semigaleatus* obtained the highest in duration (Table 4). There were three individuals for *B. h. hydrocorax*, two for *B. h. semigaleatus* and seven for *B. h. mindanensis*. Generally, the loud calls of *Buceros* can be described as a resonant honk, which is noticeably different from the raucous cackles of *Anthracoceros* and shrill cackles of *Anorrhinus* hornbills (Gonzalez et al. 2013).

A small effect size was obtained for the minimum frequency between *B. h. hydrocorax* vs. *B. h. mindanensis* (0.29), while large effect sizes for bandwidth (4.86), duration (1.97), maximum frequency (4.94) and peak frequency (4.95) (for the same species pair?). A score of 3 was given to the species pair upon acquiring minor and medium values. In comparison, *B. h. hydrocorax* vs. *B. h. semigaleatus* and *B. h. semigaleatus* vs. *B. h. mindanensis*, obtained large effect sizes for all vocal characters, hence earning cumulative scores of 8: bandwidth (4.21 and 12.41), duration (11.23 and 19.71), minimum frequency (13.02 and 16.67), maximum frequency (8.58 and 18.34) and peak frequency (13.10 and 16.64), respectively.

In comparison with the *Penelopides*, *Rhabdotorrhinus* and *Rhyticeros*, the allopatric species of *Buceros hydrocorax* obtained relatively low frequencies. Lower frequencies in *Buceros hydrocorax* was correlated with the prominent casque size (Alexander et al. 1994, Policht et al. 2009). The threshold for the phenotypic score and molecular divergence are 14 and 4%, respectively. Due to speciation being comprised of phenotype and genotype, the combination of the phenotypic and genotypic data will lead to a more precise taxonomic evaluation, most especially for the species in the biodiversity hotspots. Moreover, the recent studies validated the splits of *Aceros* and *Penelopides* and the probability of splitting *B. h. semigaleatus* and *B. h. mindanensis* from the nominotypical *B. h. hydrocorax* (Kemp and Crowe 1985, Gonzalez 2012, Gonzalez et al. 2013).

**Figure 2.**
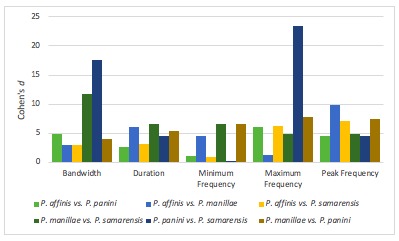
Summary of Cohen’s *d* of the allopatric species pairs for species pairs of *P. affinis, P. manillae, P. panini* and *P. samarensis* in vocal characters.

**Figure 3.**
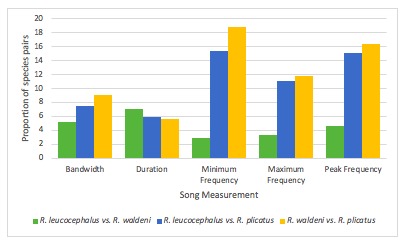
The summary of Cohen’s *d* of the species pairs species pairs of *R. leucocephalus, R. waldeni* and *R. plicatus* in vocal characters.

**Figure 4.**
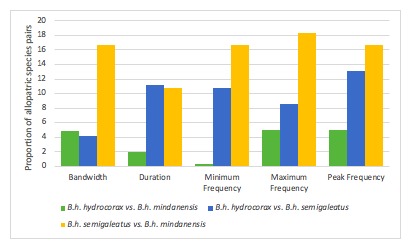
The summary of Cohen’s *d* of the allopatric species pairs of *B. h. hydrocorax, B. h. semigaleatus* and *B. h. mindanensis.* in vocal characters.

As seen in Table 5, all of the species and subspecies pairs obtained a phenotypic score not lower than 14 and have evidently reached the threshold value of 7. This combined phenotypic data with the newly quantified acoustic evaluation supports the proposition that subspecies within the *B. hydrocorax* complex required further review on their taxonomic status (Gonzalez 2012, see also Figs 2, 3, 4, 5, 6, 7).

Amongst the endemic Philippine Tarictic hornbills, small effect sizes for the minimum frequency were evident between *P. panini* and *P. samarensis.* However, large effect sizes were obtained from the bandwidth, duration, minimum frequency and maximum frequency of *P. affinis* vs. *P. panini, P. affinis* vs. *P. manillae, P. affinis* vs. *P. samarensis, P. manillae* vs. *P. samarensis, P. panini* vs. *P. samarensis* and *P. manillae* vs. *P. panini.* A distinctive trumpeting bleat which is highly onomatopoeic of its local name "Tarik-tik" or "Talik-tik", can be collectively referred to all members of the Philippine endemic genus *Penelopides.* These high pitched calls of Tarictic hornbills are comparable to the similarly toned staccato bark of the genus *Rhabdotorrhinus* to which they are closely related, but conversely *Penelopides* have relatively higher frequencies (Gonzalez et al. 2013). See Table 6, Suppl. materials 1, 2 for the raw data, data summary and spectrograms of this study.

## Conclusions

The above acoustic analyses of the trumpeting calls of Philippine Tarictic hornbills, belonging to the endemic genus *Penelopides*, support their genus allocation and distinction from the closely related genus *Rhabdotorrhinus* characterised with its staccato calls (see also Gonzales 2012, Gonzalez et al. 2013). Overall, casque peak frequency is associated with the fundamental frequency, which is reflected in hornbills having higher pitched vocalisations as compared to *Buceros hydrocorax, Rhyticeros* and *Rhabdotorrhinus*.

The large effect sizes in the acoustic data, observed amongst subspecies of *B. hydrocorax*, provide additional support on their proposed taxonomic revisions and potential split, subsequently based on the initial analysis using phenotypic and genetic data presented by Gonzalez (2012). Further comparative analysis of the three subspecies using call recordings from the wild may provide better insights into their taxonomic status. Although this study was largely limited to captive hornbills, further bioacoustic analysis may be needed, based on a wider breadth of sampling and done in comparison with added sampling of species in the wild. Essentially, providing combined phenotypic and vocal data will help support probable differences attributed to evolutionary adaptation that often befits speciation in tropical ecosystems. A better understanding of the taxonomic status of a group of threatened species like Philippine hornbills is invaluable to their effective conservation and management, both in captivity and in the wild.

## Supplementary Material

3ED93D54-E8B3-595D-8F4D-69BF81056E28 10.3897/BDJ.7.e31723.suppl1 Supplementary material 1 Raw Data of Vocalization Data type: Measurements of the vocal characters
File: oo_319775.docx https://binary.pensoft.net/file/319775 Shari L. Guerra

58662592-F20D-582B-9F4D-79E30ACA74D6 10.3897/BDJ.7.e31723.suppl2 Supplementary material 2 Spectrogram Data type: Images of spectrogram
File: oo_319782.pdf https://binary.pensoft.net/file/319782 Shari L. Guerra

## Figures and Tables

**Figure 5a.**
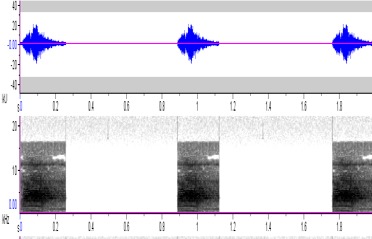
*Penelopides affinis*

**Figure 5b.**
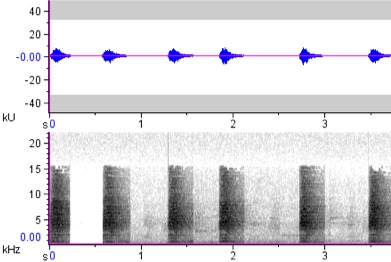
*Penelopides manillae*

**Figure 5c.**
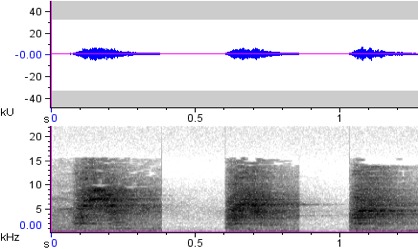
*Penelopides panini*

**Figure 5d.**
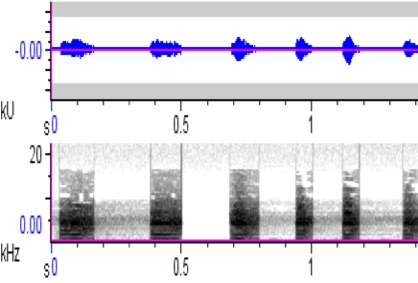
*Penelopides samarensis*

**Figure 6a.**
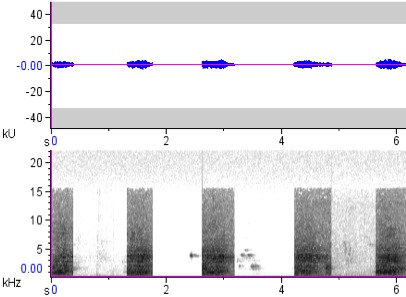
*Rhabdotorrhinus leucocephalus*

**Figure 6b.**
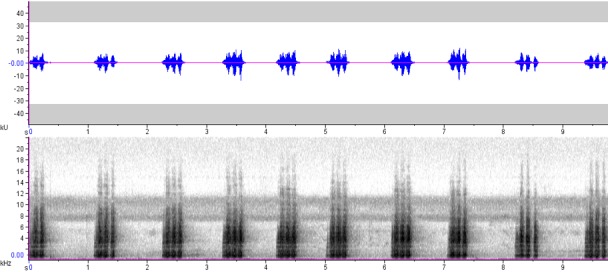
*Rhabdotorrhinus waldeni*

**Figure 6c.**
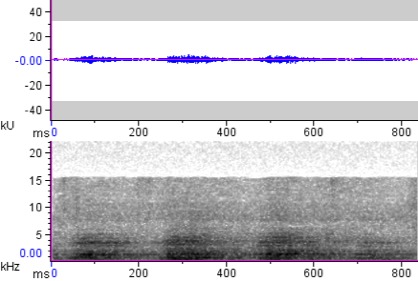
*Rhyticeros plicatus*

**Figure 7a.**
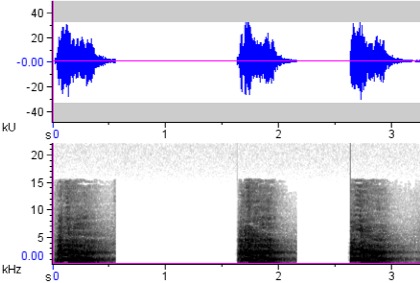
*Buceros hydrocorax hydrocorax*

**Figure 7b.**
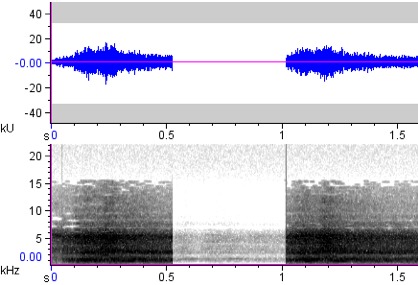
*Buceros hydrocorax semigaleatus*

**Figure 7c.**
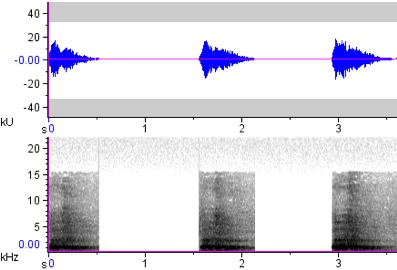
*Buceros hydrocorax mindanensis*

**Table 1.** Summary of phenotypic scoring procedures (Tobias et al. 2010).

**Trait**	**Magnitude (Score)**				
	**Frequency of scoring**	**Minor (1)**	**Medium (2)**	**Major (3)**	**Exceptional (4)**
Morphology (biometrics)	Strongest increase and strongest decrease only	Effect size: 0.2-2	Effect size: 2-5	Effect size: 5-10	Effect size: >10
Acoustics	Strongest temporal and spectral character only	Effect size: 0.2-2	Effect size: 2-5	Effect size: 5-10	Effect size: >10
Plumage and bare parts	Three strongest characters	A slightly different wash or suffusion to all parts of any area	Distinctly different tone/shade to all or part of a significant area of feathering	Contrastingly different hue/colour to all or part of a significant part of a significant area of feathering	Radically different colouration or pattern to most of plumage (striking contrast in colour, shade, shape)
Geographical relationship	n/a	Broad hybrid zone	Narrow hybrid zone	Parapatry	n/a

**Table 2.** Summary of mean, standard deviation, number of individuals, pooled variance, Cohen’s *d* score and total score using a standard quantitative criteria (Tobias et al. 2010) for compared species of *Penelopides sp*.

Vocal Characters	Mean	SD	n	Mean	SD	n	Pooled variance	Cohen's d	Score	Total Score
	*P. affinis*			*P. panini*						
Bandwidth	4.6735	0.2377	6	5.5377	0.0907	4	0.1753	4.93	2	5
Duration	0.1875	0.0148	6	0.1571	0.0091	4	0.01	2.63	2*	
Minimum Frequency	2.0346	0.2023	6	1.8686	0.071	4	0.1482	1.12	1	
Maximum Frequency	6.7081	0.1495	6	7.4063	0.0841	4	0.1153	6.06	3**	
Peak Frequency	4.7272	0.1604	6	4.171	0.0811	4	0.1218	4.57	2	
*P. affinis*				*P. manillae*						
Bandwidth	4.6735	0.2377	6	5.1882	0.1053	5	0.1724	2.99	2	6
Duration	0.1875	0.0148	6	0.1244	0.0046	5	0.01	6.1	3*	
Minimum Frequency	2.0346	0.2023	6	1.3492	0.0995	5	0.149	4.6	2	
Maximum Frequency	6.7081	0.1495	6	6.5374	0.15	5	0.1354	1.26	1	
Peak Frequency	4.7272	0.1604	6	3.2542	0.1704	5	0.1492	9.88	3**	
*P. affinis*				*P. samarensis*						
Bandwidth	4.6735	0.2377	6	4.1438	0.0929	4	0.1756	3.02	2	5
Duration	0.1875	0.0148	6	0.2562	0.0345	4	0.02	3.18	2*	
Minimum Frequency	2.0346	0.2023	6	1.8855	0.0792	4	0.1495	1	1	
Maximum Frequency	6.7081	0.1495	6	6.0293	0.0456	4	0.1086	6.25	3	
Peak Frequency	4.7272	0.1604	6	3.8559	0.0806	4	0.1217	7.16	3**	
*P. manillae*				*P. samarensis*						
Bandwidth	5.1882	0.1053	5	4.1438	0.0929	4	0.0884	11.82	4**	7
Duration	0.1244	0.0046	5	0.2562	0.0345	4	0.02	6.55	3*	
Minimum Frequency	1.3492	0.0995	5	1.8855	0.0792	4	0.0806	6.66	3	
Maximum Frequency	6.5374	0.15	5	6.0293	0.0456	4	0.1034	4.91	2	
Peak Frequency	3.2542	0.1704	5	3.8559	0.0806	4	0.1227	4.9	2	
*P. panini*				*P. samarensis*						
Bandwidth	5.5377	0.0907	4	4.1438	0.0929	4	0.0795	17.53	4	6
Duration	0.1571	0.0091	4	0.2562	0.0345	4	0.02	4.54	2*	
Minimum Frequency	1.8686	0.071	4	1.8855	0.0792	4	0.0651	0.26	1	
Maximum Frequency	7.4063	0.0841	4	6.0293	0.0456	4	0.0586	23.51	4**	
Peak Frequency	4.171	0.0811	4	3.8559	0.0806	4	0.07	4.5	2	
*P. manillae*				*P. panini*						
Bandwidth	5.1882	0.1053	5	5.5377	0.0907	4	0.09	3.99	2	6
Duration	0.1244	0.0046	5	0.1571	0.0091	4	0.01	5.39	3*	
Minimum Frequency	1.3492	0.0995	5	1.8686	0.071	4	0.08	6.66	3	
Maximum Frequency	6.5374	0.15	5	7.4063	0.0841	4	0.11	7.82	3**	
Peak Frequency	3.2542	0.1704	5	4.171	0.0811	4	0.12	7.46	3	
*strongest temporal character		**strongest spectral character								

**Table 3.** Summary of mean, standard deviation, number of individuals, pooled variance, Cohen’s *d* score and total score score using a standard quantitative criteria (Tobias et al. 2010) for compared species amongst *Rhabdotorrhinus leucocephalus, Rhabdotorrhinus waldeni* and *Rhyticeros plicatus*.

Vocal Characters	Mean	SD	n	Mean	SD	n	Pooled variance	Cohen's d	Score	Total Score
	*R. leucocephalus*			*R. waldeni*						
Bandwidth	3.8984	0.0901	5	4.2894	0.1198	1	0.0736	5.31	3**	6
Duration	0.2878	0.0151	5	0.2	0.0145	1	0.01	7.14	3*	
Minimum Frequency	0.7293	0.041	5	0.6288	0.0189	1	0.0335	3	2	
Maximum Frequency	4.6244	0.1046	5	4.9182	0.1284	1	0.0854	3.44	2	
Peak Frequency	2.5908	0.1454	5	2.0413	0.364	1	0.1187	4.63	2	
*R. leucocephalus*				*R. plicatus*						
Bandwidth	3.8984	0.0901	5	3.0039	0.1983	3	0.1179	7.59	4	7
Duration	0.2878	0.0151	5	0.5875	0.0978	3	0.05	5.99	3*	
Minimum Frequency	0.7293	0.041	5	0.2261	0.0303	3	0.0327	15.38	4**	
Maximum Frequency	4.6244	0.1046	5	3.23	0.2017	3	0.1251	11.15	4	
Peak Frequency	2.5908	0.1454	5	0.8506	0.1025	3	0.1149	15.15	4	
*R. waldeni*				*R. plicatus*						
Bandwidth	4.2894	0.1198	1	3.0039	0.1983	3	0.1402	9.17	3	7
Duration	0.2	0.0145	1	0.5875	0.0978	3	0.07	5.6	3*	
Minimum Frequency	0.6288	0.0189	1	0.2261	0.0303	3	0.0214	18.78	4**	
Maximum Frequency	4.9182	0.1284	1	3.23	0.2017	3	0.1426	11.84	4	
Peak Frequency	2.0413	0.364	1	0.8506	0.1025	3	0.0725	16.43	4	
*strongest temporal character		**strongest spectral character								

**Table 4.** Summary of mean, standard deviation, number of individuals, pooled variance, Cohen’s *d* score and total score score using a standard quantitative criteria (Tobias et al. 2010) for compared subspecies of *B. hydrocorax*.

Vocal Characters	Mean	SD	n	Mean	SD	n	Pooled variance	Cohen's d	Score	Total Score
	*B. h. hydrocorax*			*B. h. mindanensis*						
Bandwidth	2.1809	0.0945	3	2.493	0.0624	7	0.0642	4.86	2	3
Duration	0.292	0.0226	3	0.2713	0.0038	7	0.01	1.97	1*	
Minimum Frequency	0.5065	0.0159	3	0.5035	0.0093	7	0.0101	0.29	1	
Maximum Frequency	2.6873	0.0894	3	2.9965	0.0621	7	0.0626	4.94	2	
Peak Frequency	0.8234	0.0123	3	0.9223	0.0248	7	0.02	4.95	2**	
*B. h. hydrocorax*				*B. h. semigaleatus*						
Bandwidth	2.1809	0.0945	3	4.6767	0.022	2	0.06	4.21	2	8
Duration	0.292	0.0226	3	0.563	0.0434	2	0.02	11.23	4*	
Minimum Frequency	0.5065	0.0159	3	0.7911	0.023	2	0.01	13.02	4	
Maximum Frequency	2.6873	0.0894	3	5.4678	0.0197	2	0.06	8.58	3	
Peak Frequency	0.8234	0.0123	3	3.1199	0.3915	2	0.18	13.1	4**	
*B. h. hydrocorax*				*B. h. mindanensis*						
Bandwidth	2.1809	0.0945	3	2.493	0.0624	7	0.0642	4.86	2	3
Duration	0.292	0.0226	3	0.2713	0.0038	7	0.01	1.97	1*	
Minimum Frequency	0.5065	0.0159	3	0.5035	0.0093	7	0.0101	0.29	1	
Maximum Frequency	2.6873	0.0894	3	2.9965	0.0621	7	0.0626	4.94	2	
Peak Frequency	0.8234	0.0123	3	0.9223	0.0248	7	0.02	4.95	2**	
*strongest temporal character		**strongest spectral character								

**Table 5.** Phenotypic scores and molecular divergence (Gonzalez 2012) for species/subspecies pairs based on the quantitative criteria for species delimitation by Tobias et al. (2010).

**Species/subspecies pair**		**Phenotypic scores**				**Total phenotypic score**	% **molecular divergence**
		**Biometrics**	**Plumage and bare parts**	**Vocalisation**	**Geographical relationship**		
**1**	*Penelopides affinis* *Penelopides manillae*	2	6	6	0	14	3.52
**2**	*Penelopides panini* *Penelopides manillae*	3	6	6	0	15	4.53
**3**	*Penelopides panini* *Penelopides affinis*	3	7	5	0	15	3.4
**4**	*Penelopides samarensis* *Penelopides affinis*	2	5	5	0	12	2.06
**5**	*Buceros hydrocorax hydrocorax* *Buceros hydrocorax mindanensis*	2	7	3	0	12	8.85
**6**	*Buceros hydrocorax hydrocorax* *Buceros hydrocorax semigaleatus*	2	7	8	0	17	11.56
**7**	*Buceros hydrocorax semigaleatus* *Buceros hydrocorax mindanensis*	2	2	8	0	12	8.22
**8**	*Rhabdotorrhinus leucocephalus* *Rhabdotorrhinus waldeni*	3	6	6	0	15	5.36

**Table 6.** Recording sources for the Philippine hornbill vocalisations.

**QTY**	**SPECIES**	**RECORDIST**	**LOCALITY**
**2**	*Penelopides affinis*	Frank Lambert	Zamboanga, Pasonaca Watershed Reserve, Cabonegro
**1**	*Penelopides affinis*	Frank Lambert	Mt. Kitanglad, Mindanao
**1**	*Penelopides affinis*	Paul Noakes	PICOP, Bislig, Mindanao
**1**	*Penelopides affinis*	George Wagner	Baluno Station, Zamboanga Watershed, Mindanao
**1**	*Penelopides affinis*	David Edwards	PICOP, Bislig, Mindanao
**4**	*Penelopides manillae*	Shari Guerra	Avilon Zoo, Rodriguez, Rizal
**1**	*Penelopides manillae*	David Edwards	Hamut, baliuag, Sierra Madre Mountains, Luzon
**3**	*Penelopides panini*	Shari Guerra	Avilon Zoo, Rodriguez, Rizal
**1**	*Penelopides panini*	Frank Lambert	Bacolod, Negros Occidental
**3**	*Penelopides samarensis*	Bram Demeulemeester	Rajah Sikatuna National Park, Bohol
**1**	*Penelopides samarensis*	Ross Gallardy	Rajah Sikatuna National Park, Bohol
**3**	*Buceros hydrocorax hydrocorax*	Shari Guerra	Avilon Zoo, Rodriguez, Rizal
**2**	*Buceros hydrocorax semigaleatus*	Shari Guerra	Avilon Zoo, Rodriguez, Rizal
**7**	*Buceros hydrocorax mindanensis*	Shari Guerra	Avilon Zoo, Rodriguez, Rizal
**3**	*Rhabdotorrhinus leucocephalus*	Shari Guerra	Avilon Zoo, Rodriguez, Rizal
**2**	*Rhabdotorrhinus leucocephalus*	Desmond Allen	Sitio Siete, South Cotabato Province, Mindanao
**1**	*Rhabdotorrhinus waldeni*	Ross Gallardy	PICOP, Bislig, Mindanao
**1**	*Rhyticeros plicatus*	Shari Guerra	Avilon Zoo, Rodriguez, Rizal
**1**	*Rhyticeros plicatus*	Frank Lambert	Chupukama Ridge, Guadalcanal
**1**	*Rhyticeros plicatus*	Patrick Abueg	Lolobata National Park, Halmahera, Indonesia

## References

[B4711658] Alexander G. D., Houston D. C., Campbell M. (1994). A possible acoustic function for the casque structure in hornbills (Aves: Bucerotidae). Journal of Zoology.

[B4711678] Becker L A (2000). Effect Size (ES).

[B4718874] Cohen J. (1988). Statistical power analysis for the behavioral sciences.

[B4711801] Gonzalez J C T (2012). Origin and diversification of hornbills (Bucerotidae). Doctoral dissertation..

[B4711810] Gonzalez J C T, Sheldon B C., Collar N J., Tobias J A. (2013). A comprehensive molecular phylogeny for the hornbills (Aves: Bucerotidae). Molecular Phylogenetics and Evolution.

[B4711820] Haimoff E. H. (2008). A spectrographic analysis of the loud calls of helmeted hornbills *Rhinoplax
vigil*. Ibis.

[B4712134] Kemp AC, Crowe TM (1985). The systematics and zoogeography of oriental and Australasian hornbills (Aves: Bucerotidae).. Bonner Zoologische Beiträge.

[B4712144] Kemp A. C. (1995). The Hornbills.

[B4795889] Kemp A C, Poonswad P (1998). Techniques of bird sound recording for sonogram analysis. The Asian hornbills: Ecology and conservation. Thai Studies in Biodiversity.

[B4717054] Kennedy R S, Gonzales P C, Miranda H C, Fisher T H (2000). A guide to the birds of the Philippines.

[B5360138] Kinnaird Margaret F., O'Brien Timothy G. (2007). The ecology and conservation of Asian hornbills: Farmers of the forest.

[B4712164] Lum S, Poonswad P (2005). The ecology of hornbills: Reproduction and populations.

[B4711943] Policht R., Petrů M., Lastimoza L., Suarez L. (2009). Potential for the use of vocal individuality as a conservation research tool in two threatened Philippine hornbill species, the Visayan hornbill and the Rufous-headed hornbill. Bird Conservation International.

[B5360129] Poonswad Pilai, Kemp Alan, Strange Morten (2013). Hornbills of the World: A photographic guide.

[B4712009] Tobias J. A., Seddon N., Spottiswoode C. N., Pilgrim J. D., Fishpool L. D. C., Collar N. J. (2010). Quantitative criteria for species delimitation. Ibis.

