# Supplementary material for: Description and comparison of Philippine hornbill (Bucerotidae) vocalizations
Source: Biodivers Data J. 2019 Nov 13;7:e31723. doi: 10.3897/BDJ.7.e31723 (PMC6868047; doi:10.3897/BDJ.7.e31723)
Supplement: Supplementary material 1 — Raw Data of Vocalization [file bdj-07-e31723-s001.docx]

Appendix C.1. Measurements of the vocal characters for *P. affinis*.

| Bandwidth  (Hz) | Duration (s) | Minimum Frequency  (Hz) | Maximum Frequency  (Hz) | Peak Frequency  (Hz) |
| --- | --- | --- | --- | --- |
| 4134.4 | 0.1 | 4306.6 | 8441 | 6890.6 |
| 3617.6 | 0.1 | 4306.6 | 7924.2 | 7062.9 |
| 3962.1 | 0.1 | 4478.9 | 8441 | 6718.4 |
| 4478.9 | 0.1 | 3445.3 | 7924.2 | 7062.9 |
| 4995.7 | 0.1 | 3273 | 8268.8 | 4995.7 |
| 6029.3 | 0.1 | 3273 | 9302.3 | 7235.2 |
| 2584 | 0.1 | 2756.2 | 5340.2 | 3962.1 |
| 2928.5 | 0.1 | 2928.5 | 5857 | 4478.9 |
| 2928.5 | 0.1 | 2756.2 | 5684.8 | 4823.4 |
| 3962.1 | 0.1 | 2411.7 | 6373.8 | 4995.7 |
| 3100.8 | 0.2 | 2928.5 | 6029.3 | 4823.4 |
| 2411.7 | 0.1 | 3445.3 | 5857 | 4651.2 |
| 2928.5 | 0.1 | 3100.8 | 6029.3 | 4823.4 |
| 2239.5 | 0.1 | 3445.3 | 5684.8 | 4651.2 |
| 2067.2 | 0.1 | 3617.6 | 5684.8 | 4478.9 |
| 2584 | 0.2 | 3445.3 | 6029.3 | 3962.1 |
| 2239.5 | 0.1 | 3617.6 | 5857 | 4134.4 |
| 2067.2 | 0.1 | 3617.6 | 5684.8 | 4478.9 |
| 6373.8 | 0.1 | 2067.2 | 8441 | 4651.2 |
| 6029.3 | 0.2 | 1894.9 | 7924.2 | 4823.4 |
| 3789.8 | 0.1 | 3273 | 7062.9 | 5512.5 |
| 4995.7 | 0.1 | 2584 | 7579.7 | 4651.2 |
| 4134.4 | 0.1 | 2756.2 | 6890.6 | 4306.6 |
| 3100.8 | 0.1 | 3617.6 | 6718.4 | 4995.7 |
| 1550.4 | 0.1 | 4134.4 | 5684.8 | 4651.2 |
| 5340.2 | 0.2 | 0 | 5340.2 | 172.3 |
| 4306.6 | 0.1 | 1722.7 | 6029.3 | 4823.4 |
| 4134.4 | 0.1 | 1894.9 | 6029.3 | 4651.2 |
| 5437.5 | 0.4 | 750 | 6187.5 | 2437.5 |
| 8625 | 0.4 | 562.5 | 9187.5 | 4312.5 |
| 6562.5 | 0.2 | 562.5 | 7125 | 4125 |
| 5437.5 | 0.3 | 750 | 6187.5 | 4312.5 |

| Appendix C.1. Continued… | | | | |
| --- | --- | --- | --- | --- |
| Bandwidth  (Hz) | **Duration (s)** | **Minimum Frequency**  **(Hz)** | **Maximum Frequency**  **(Hz)** | **Peak Frequency**  **(Hz)** |
| 6187.5 | 0.3 | 375 | 6562.5 | 4500 |
| 5437.5 | 0.4 | 750 | 6187.5 | 4875 |
| 5812.5 | 0.3 | 562.5 | 6375 | 5250 |
| 6000 | 0.3 | 562.5 | 6562.5 | 3750 |
| 5812.5 | 0.2 | 562.5 | 6375 | 4687.5 |
| 4875 | 0.2 | 1125 | 6000 | 4687.5 |
| 5625 | 0.2 | 750 | 6375 | 4500 |
| 5625 | 0.3 | 562.5 | 6187.5 | 4500 |
| 6375 | 0.3 | 562.5 | 6937.5 | 4687.5 |
| 5812.5 | 0.3 | 562.5 | 6375 | 4312.5 |
| 5625 | 0.3 | 562.5 | 6187.5 | 4875 |
| 5625 | 0.3 | 562.5 | 6187.5 | 4875 |
| 5625 | 0.3 | 562.5 | 6187.5 | 4875 |
| 6187.5 | 0.2 | 562.5 | 6750 | 4125 |
| 7875 | 0.2 | 750 | 8625 | 5437.5 |
| 6750 | 0.4 | 562.5 | 7312.5 | 4312.5 |

Appendix C.2. Measurements of the vocal characters for *P. manillae*.

| Bandwidth  (Hz) | Duration (s) | Minimum Frequency  (Hz) | Maximum Frequency  (Hz) | Peak Frequency  (Hz) |
| --- | --- | --- | --- | --- |
| 5512.5 | 0.1 | 2584 | 8096.5 | 4995.7 |
| 5512.5 | 0.1 | 2928.5 | 8441 | 5168 |
| 3962.1 | 0.2 | 689.1 | 4651.2 | 2756.2 |
| 3789.8 | 0.1 | 1550.4 | 5340.2 | 3273 |
| 4823.4 | 0.1 | 1550.4 | 6373.8 | 3445.3 |
| 5512.5 | 0.1 | 2584 | 8096.5 | 4995.7 |
| 4823.4 | 0.1 | 3617.6 | 8441 | 5168 |
| 3273 | 0.1 | 2584 | 5857 | 4306.6 |
| 6201.6 | 0.1 | 2928.5 | 9130.1 | 3962.1 |
| 5168 | 0.1 | 3273 | 8441 | 4995.7 |
| 6373.8 | 0.1 | 2239.5 | 8613.3 | 5857 |
| 5684.8 | 0.1 | 2928.5 | 8613.3 | 6201.6 |
| 5168 | 0.1 | 3100.8 | 8268.8 | 4306.6 |
| 5340.2 | 0.1 | 3100.8 | 8441 | 4478.9 |
| 4995.7 | 0.1 | 3100.8 | 8096.5 | 4306.6 |
| 6029.3 | 0.1 | 2928.5 | 8957.8 | 3789.8 |
| 6373.8 | 0.1 | 2756.2 | 9130.1 | 4134.4 |
| 5857 | 0.1 | 3100.8 | 8957.8 | 6029.3 |
| 6890.6 | 0.1 | 1722.7 | 8613.3 | 4823.4 |
| 6890.6 | 0.1 | 1894.9 | 8785.5 | 8613.3 |
| 7407.4 | 0.1 | 689.1 | 8096.5 | 3617.6 |
| 7579.7 | 0.1 | 1722.7 | 9302.3 | 3617.6 |
| 7062.9 | 0.1 | 1894.9 | 8957.8 | 6029.3 |
| 5340.2 | 0.1 | 2928.5 | 8268.8 | 6029.3 |
| 5857 | 0.1 | 3100.8 | 8957.8 | 4306.6 |
| 6373.8 | 0.1 | 2928.5 | 9302.3 | 4306.6 |
| 9474.6 | 0.1 | 689.1 | 10163.7 | 4134.4 |
| 3100.8 | 0.1 | 2756.2 | 5857 | 5168 |
| 3962.1 | 0.2 | 2067.2 | 6029.3 | 4995.7 |
| 3617.6 | 0.1 | 2584 | 6201.6 | 4134.4 |
| 4134.4 | 0.1 | 2584 | 6718.4 | 3962.1 |
| 4134.4 | 0.1 | 2756.2 | 6890.6 | 3962.1 |

| Appendix C.2. Continued… | | | | |
| --- | --- | --- | --- | --- |
| Bandwidth  (Hz) | **Duration (s)** | **Minimum Frequency**  **(Hz)** | **Maximum Frequency**  **(Hz)** | **Peak Frequency**  **(Hz)** |
| 6201.6 | 0.1 | 861.3 | 7062.9 | 3273 |
| 6546.1 | 0.1 | 2411.7 | 8957.8 | 3273 |
| 7062.9 | 0.1 | 2584 | 9646.9 | 3273 |
| 6890.6 | 0.1 | 2756.2 | 9646.9 | 4478.9 |
| 8096.5 | 0.1 | 1550.4 | 9646.9 | 3100.8 |
| 8096.5 | 0.1 | 1033.6 | 9130.1 | 4306.6 |
| 5340.2 | 0.1 | 2928.5 | 8268.8 | 3789.8 |
| 8268.8 | 0.2 | 861.3 | 9130.1 | 3617.6 |
| 6890.6 | 0.1 | 1722.7 | 8613.3 | 4306.6 |
| 4823.4 | 0.1 | 2756.2 | 7579.7 | 3445.3 |
| 5512.5 | 0.1 | 2756.2 | 8268.8 | 3962.1 |
| 6890.6 | 0.1 | 1205.9 | 8096.5 | 4306.6 |
| 5684.8 | 0.1 | 2756.2 | 8441 | 3962.1 |
| 7235.2 | 0.1 | 1722.7 | 8957.8 | 3962.1 |
| 7235.2 | 0.1 | 2239.5 | 9474.6 | 5857 |
| 2928.5 | 0.1 | 2756.2 | 5684.8 | 3273 |
| 3273 | 0.1 | 2584 | 5857 | 3100.8 |
| 3962.1 | 0.1 | 2411.7 | 6373.8 | 3273 |
| 3100.8 | 0.1 | 2584 | 5684.8 | 3273 |
| 2756.2 | 0.1 | 2239.5 | 4995.7 | 3100.8 |
| 4823.4 | 0.2 | 516.8 | 5340.2 | 3445.3 |
| 4134.4 | 0.1 | 1033.6 | 5168 | 3789.8 |
| 4306.6 | 0.2 | 516.8 | 4823.4 | 3617.6 |
| 4823.4 | 0.1 | 1205.9 | 6029.3 | 4306.6 |
| 5512.5 | 0.1 | 1033.6 | 6546.1 | 5512.5 |
| 5684.8 | 0.1 | 1205.9 | 6890.6 | 3962.1 |
| 4651.2 | 0.1 | 1033.6 | 5684.8 | 5512.5 |
| 4478.9 | 0.1 | 1550.4 | 6029.3 | 3273 |
| 4478.9 | 0.1 | 1033.6 | 5512.5 | 3100.8 |
| 4478.9 | 0.1 | 1033.6 | 5512.5 | 3100.8 |
| 4651.2 | 0.1 | 1722.7 | 6373.8 | 3962.1 |
| 4995.7 | 0.1 | 1033.6 | 6029.3 | 4478.9 |
| 3962.1 | 0.1 | 1722.7 | 5684.8 | 3789.8 |

Appendix C.2. Continued…

| Bandwidth  (Hz) | Duration (s) | Minimum Frequency  (Hz) | Maximum Frequency  (Hz) | Peak Frequency  (Hz) |
| --- | --- | --- | --- | --- |
| 5168 | 0.1 | 1033.6 | 6201.6 | 3273 |
| 5168 | 0.2 | 516.8 | 5684.8 | 3273 |
| 5857 | 0.1 | 344.5 | 6201.6 | 1033.6 |
| 5684.8 | 0.1 | 344.5 | 6029.3 | 516.8 |
| 4478.9 | 0.1 | 516.8 | 4995.7 | 3273 |
| 4651.2 | 0.1 | 1550.4 | 6201.6 | 4651.2 |
| 5168 | 0.2 | 0 | 5168 | 0 |
| 4995.7 | 0.1 | 0 | 4995.7 | 172.3 |
| 4651.2 | 0.1 | 0 | 4651.2 | 0 |
| 4651.2 | 0.1 | 0 | 4651.2 | 0 |
| 4823.4 | 0.2 | 0 | 4823.4 | 3789.8 |
| 5684.8 | 0.2 | 0 | 5684.8 | 0 |
| 4823.4 | 0.1 | 0 | 4823.4 | 0 |
| 4823.4 | 0.1 | 0 | 4823.4 | 172.3 |
| 4651.2 | 0.1 | 0 | 4651.2 | 0 |
| 5168 | 0.2 | 0 | 5168 | 4478.9 |
| 4823.4 | 0.2 | 0 | 4823.4 | 3273 |
| 5340.2 | 0.2 | 0 | 5340.2 | 0 |
| 4823.4 | 0.1 | 0 | 4823.4 | 0 |
| 5340.2 | 0.1 | 0 | 5340.2 | 3273 |
| 4651.2 | 0.1 | 0 | 4651.2 | 0 |
| 4651.2 | 0.1 | 0 | 4651.2 | 172.3 |
| 4651.2 | 0.3 | 0 | 4651.2 | 0 |
| 4651.2 | 0.2 | 0 | 4651.2 | 0 |
| 4823.4 | 0.2 | 0 | 4823.4 | 3273 |
| 4478.9 | 0.2 | 0 | 4478.9 | 0 |
| 4651.2 | 0.2 | 0 | 4651.2 | 0 |
| 4478.9 | 0.1 | 0 | 4478.9 | 0 |
| 4651.2 | 0.2 | 0 | 4651.2 | 0 |
| 4651.2 | 0.3 | 0 | 4651.2 | 0 |
| 4651.2 | 0.3 | 0 | 4651.2 | 172.3 |
| 4651.2 | 0.2 | 0 | 4651.2 | 0 |

Appendix C.2. Continued…

| Bandwidth  (Hz) | Duration (s) | Minimum Frequency  (Hz) | Maximum Frequency  (Hz) | Peak Frequency  (Hz) |
| --- | --- | --- | --- | --- |
| 4651.2 | 0.3 | 0 | 4651.2 | 0 |
| 4823.4 | 0.2 | 516.8 | 5340.2 | 3445.3 |
| 4134.4 | 0.1 | 1033.6 | 5168 | 3789.8 |
| 4823.4 | 0.2 | 516.8 | 5340.2 | 3445.3 |
| 4134.4 | 0.1 | 1033.6 | 5168 | 3789.8 |
| 4306.6 | 0.2 | 516.8 | 4823.4 | 3617.6 |
| 4823.4 | 0.1 | 1205.9 | 6029.3 | 4306.6 |
| 5512.5 | 0.1 | 1033.6 | 6546.1 | 5512.5 |
| 5684.8 | 0.1 | 1205.9 | 6890.6 | 3962.1 |
| 4651.2 | 0.1 | 1033.6 | 5684.8 | 5512.5 |
| 4478.9 | 0.1 | 1550.4 | 6029.3 | 3273 |
| 4478.9 | 0.1 | 1033.6 | 5512.5 | 3100.8 |
| 4478.9 | 0.1 | 1033.6 | 5512.5 | 3100.8 |
| 4651.2 | 0.1 | 1722.7 | 6373.8 | 3962.1 |
| 4995.7 | 0.1 | 1033.6 | 6029.3 | 4478.9 |
| 3962.1 | 0.1 | 1722.7 | 5684.8 | 3789.8 |
| 5168 | 0.1 | 1033.6 | 6201.6 | 3962.1 |
| 5168 | 0.1 | 1033.6 | 6201.6 | 3273 |
| 5168 | 0.2 | 516.8 | 5684.8 | 3273 |
| 5857 | 0.1 | 344.5 | 6201.6 | 1033.6 |
| 5684.8 | 0.1 | 344.5 | 6029.3 | 516.8 |
| 4478.9 | 0.1 | 516.8 | 4995.7 | 3273 |
| 4651.2 | 0.1 | 1550.4 | 6201.6 | 4651.2 |

Appendix C.3. Measurements of the vocal characters for *P. panini*.

| Bandwidth  (Hz) | Duration (s) | Minimum Frequency  (Hz) | Maximum Frequency  (Hz) | Peak Frequency  (Hz) |
| --- | --- | --- | --- | --- |
| 3789.8 | 0.1 | 3273 | 7062.9 | 6373.8 |
| 5512.5 | 0.1 | 2067.2 | 7579.7 | 3789.8 |
| 5340.2 | 0.2 | 2756.2 | 8096.5 | 5340.2 |
| 2756.2 | 0.1 | 3617.6 | 6373.8 | 5168 |
| 4478.9 | 0.1 | 2928.5 | 7407.4 | 4995.7 |
| 4134.4 | 0.1 | 2756.2 | 6890.6 | 5340.2 |
| 4134.4 | 0.1 | 3100.8 | 7235.2 | 5340.2 |
| 3617.6 | 0.1 | 2928.5 | 6546.1 | 5340.2 |
| 4651.2 | 0.2 | 1894.9 | 6546.1 | 5168 |
| 6201.6 | 0.1 | 516.8 | 6718.4 | 3100.8 |
| 4651.2 | 0.1 | 2928.5 | 7579.7 | 5340.2 |
| 4995.7 | 0.1 | 1722.7 | 6718.4 | 3100.8 |
| 4651.2 | 0.1 | 1550.4 | 6201.6 | 3617.6 |
| 4134.4 | 0.1 | 2239.5 | 6373.8 | 3617.6 |
| 4651.2 | 0.1 | 3273 | 7924.2 | 5512.5 |
| 4478.9 | 0.1 | 3100.8 | 7579.7 | 4306.6 |
| 2411.7 | 0.1 | 4134.4 | 6546.1 | 5168 |
| 6373.8 | 0.1 | 1550.4 | 7924.2 | 5168 |
| 3617.6 | 0.1 | 1722.7 | 5340.2 | 5168 |
| 6373.8 | 0.1 | 1722.7 | 8096.5 | 5168 |
| 5168 | 0.1 | 1722.7 | 6890.6 | 5168 |
| 4651.2 | 0.1 | 3445.3 | 8096.5 | 6546.1 |
| 4995.7 | 0.1 | 3445.3 | 8441 | 7407.4 |
| 4651.2 | 0.1 | 3100.8 | 7752 | 5340.2 |
| 4306.6 | 0.2 | 3789.8 | 8096.5 | 4478.9 |
| 3617.6 | 0.1 | 3445.3 | 7062.9 | 5340.2 |
| 4306.6 | 0.1 | 2928.5 | 7235.2 | 4478.9 |
| 3100.8 | 0.1 | 3789.8 | 6890.6 | 4306.6 |
| 5512.5 | 0.1 | 2067.2 | 7579.7 | 3962.1 |
| 4995.7 | 0.1 | 3445.3 | 8441 | 4134.4 |
| 5857 | 0.1 | 2928.5 | 8785.5 | 4134.4 |
| 5684.8 | 0.1 | 2928.5 | 8613.3 | 4306.6 |

Appendix C.3. Continued…

| Bandwidth  (Hz) | Duration (s) | Minimum Frequency  (Hz) | Maximum Frequency  (Hz) | Peak Frequency  (Hz) |
| --- | --- | --- | --- | --- |
| 5512.5 | 0.1 | 2411.7 | 7924.2 | 4306.6 |
| 5340.2 | 0.1 | 3445.3 | 8785.5 | 6718.4 |
| 4823.4 | 0.1 | 2928.5 | 7752 | 5684.8 |
| 4134.4 | 0.1 | 2928.5 | 7062.9 | 4478.9 |
| 4134.4 | 0.1 | 3445.3 | 7579.7 | 5340.2 |
| 4306.6 | 0.1 | 2928.5 | 7235.2 | 5340.2 |
| 4823.4 | 0.1 | 2584 | 7407.4 | 5340.2 |
| 4995.7 | 0.1 | 3273 | 8268.8 | 5512.5 |
| 4478.9 | 0.1 | 3617.6 | 8096.5 | 5340.2 |
| 8268.8 | 0.2 | 516.8 | 8785.5 | 4995.7 |
| 5857 | 0.1 | 1550.4 | 7407.4 | 4478.9 |
| 5340.2 | 0.1 | 1894.9 | 7235.2 | 4134.4 |
| 6718.4 | 0.1 | 516.8 | 7235.2 | 3962.1 |
| 6029.3 | 0.1 | 516.8 | 6546.1 | 3445.3 |
| 3789.8 | 0.1 | 2584 | 6373.8 | 3445.3 |
| 3617.6 | 0.1 | 2411.7 | 6029.3 | 3962.1 |
| 6373.8 | 0.1 | 1033.6 | 7407.4 | 3962.1 |
| 4823.4 | 0.1 | 689.1 | 5512.5 | 4306.6 |
| 4651.2 | 0.1 | 1550.4 | 6201.6 | 3100.8 |
| 6890.6 | 0.1 | 3100.8 | 9991.4 | 3617.6 |
| 6029.3 | 0.1 | 3617.6 | 9646.9 | 4134.4 |
| 5512.5 | 0.1 | 3100.8 | 8613.3 | 5340.2 |
| 5340.2 | 0.1 | 2756.2 | 8096.5 | 3962.1 |
| 3962.1 | 0.1 | 2584 | 6546.1 | 3962.1 |
| 8268.8 | 0.1 | 2756.2 | 11025 | 3789.8 |
| 6201.6 | 0.1 | 3273 | 9474.6 | 4478.9 |
| 5857 | 0.1 | 2928.5 | 8785.5 | 4478.9 |
| 6890.6 | 0.1 | 2756.2 | 9646.9 | 3962.1 |
| 6029.3 | 0.1 | 1033.6 | 7062.9 | 4134.4 |
| 5684.8 | 0.1 | 861.3 | 6546.1 | 3962.1 |
| 5857 | 0.1 | 2756.2 | 8613.3 | 4478.9 |

Appendix C.3. Continued…

| Bandwidth  (Hz) | Duration (s) | Minimum Frequency  (Hz) | Maximum Frequency  (Hz) | Peak Frequency  (Hz) |
| --- | --- | --- | --- | --- |
| 7235.2 | 0.1 | 2239.5 | 9474.6 | 3962.1 |
| 6029.3 | 0.1 | 1033.6 | 7062.9 | 3962.1 |
| 5684.8 | 0.1 | 2756.2 | 8441 | 3962.1 |
| 6201.6 | 0.4 | 689.1 | 6890.6 | 3100.8 |
| 6029.3 | 0.2 | 861.3 | 6890.6 | 3789.8 |
| 5168 | 0.1 | 2067.2 | 7235.2 | 3789.8 |
| 6718.4 | 0.1 | 2584 | 9302.3 | 3789.8 |
| 6201.6 | 0.1 | 1722.7 | 7924.2 | 6201.6 |
| 6373.8 | 0.1 | 1722.7 | 8096.5 | 3962.1 |
| 7407.4 | 0.1 | 1894.9 | 9302.3 | 4651.2 |
| 5512.5 | 0.1 | 2411.7 | 7924.2 | 3962.1 |
| 5512.5 | 0.1 | 2067.2 | 7579.7 | 4823.4 |
| 7407.4 | 0.1 | 2756.2 | 10163.7 | 3617.6 |
| 4823.4 | 0.1 | 2584 | 7407.4 | 3789.8 |
| 4651.2 | 0.1 | 2239.5 | 6890.6 | 3617.6 |
| 4134.4 | 0.1 | 2239.5 | 6373.8 | 2584 |
| 7062.9 | 0.1 | 2584 | 9646.9 | 6718.4 |
| 6718.4 | 0.8 | 1378.1 | 8096.5 | 3789.8 |
| 7579.7 | 0.1 | 689.1 | 8268.8 | 4134.4 |
| 4823.4 | 0.1 | 1550.4 | 6373.8 | 3789.8 |
| 5168 | 0.1 | 1033.6 | 6201.6 | 3617.6 |
| 5684.8 | 0.1 | 1205.9 | 6890.6 | 3445.3 |
| 6201.6 | 0.1 | 2756.2 | 8957.8 | 3617.6 |
| 4306.6 | 0.1 | 2928.5 | 7235.2 | 3789.8 |
| 4823.4 | 0.1 | 1894.9 | 6718.4 | 3617.6 |
| 6546.1 | 0.1 | 344.5 | 6890.6 | 3962.1 |
| 6546.1 | 0.1 | 172.3 | 6718.4 | 3962.1 |
| 3617.6 | 0.1 | 2239.5 | 5857 | 3617.6 |
| 5625 | 0.1 | 1875 | 7500 | 5437.5 |
| 5062.5 | 0.1 | 2437.5 | 7500 | 6187.5 |

Appendix C.3. Continued…

| Bandwidth  (Hz) | Duration (s) | Minimum Frequency  (Hz) | Maximum Frequency  (Hz) | Peak Frequency  (Hz) |
| --- | --- | --- | --- | --- |
| 7312.5 | 0.1 | 750 | 8062.5 | 3750 |
| 6937.5 | 0.1 | 750 | 7687.5 | 3187.5 |
| 4875 | 0.1 | 1875 | 6750 | 3937.5 |
| 5812.5 | 0.1 | 1687.5 | 7500 | 5625 |
| 6375 | 0.1 | 2062.5 | 8437.5 | 4125 |
| 6375 | 0.1 | 1875 | 8250 | 7312.5 |
| 6562.5 | 0.1 | 1875 | 8437.5 | 5812.5 |
| 6000 | 0.1 | 1687.5 | 7687.5 | 2812.5 |
| 6750 | 0.1 | 1687.5 | 8437.5 | 4312.5 |
| 6750 | 0.1 | 1687.5 | 8437.5 | 3000 |
| 6187.5 | 0.1 | 1875 | 8062.5 | 2812.5 |
| 6937.5 | 0.1 | 1687.5 | 8625 | 2625 |
| 6937.5 | 0.1 | 1687.5 | 8625 | 2812.5 |
| 6187.5 | 0.1 | 1875 | 8062.5 | 3000 |
| 5812.5 | 0 | 1687.5 | 7500 | 5812.5 |
| 6187.5 | 0.1 | 2437.5 | 8625 | 7125 |
| 5437.5 | 0.1 | 1312.5 | 6750 | 3750 |
| 6000 | 0.1 | 1687.5 | 7687.5 | 5062.5 |
| 6375 | 0.1 | 1687.5 | 8062.5 | 2812.5 |
| 6187.5 | 0.1 | 1875 | 8062.5 | 2625 |
| 7500 | 0.1 | 1312.5 | 8812.5 | 6000 |
| 6562.5 | 0.1 | 1687.5 | 8250 | 2812.5 |
| 4875 | 0.1 | 2437.5 | 7312.5 | 4687.5 |
| 4875 | 0.1 | 2437.5 | 7312.5 | 5812.5 |
| 4687.5 | 0 | 2812.5 | 7500 | 5812.5 |
| 6029.3 | 0.2 | 861.3 | 6890.6 | 3273 |
| 7579.7 | 1 | 344.5 | 7924.2 | 2928.5 |
| 5684.8 | 0.2 | 861.3 | 6546.1 | 2928.5 |
| 3100.8 | 0.3 | 1722.7 | 4823.4 | 2411.7 |
| 5512.5 | 0.2 | 1033.6 | 6546.1 | 3789.8 |
| 4823.4 | 0.2 | 1550.4 | 6373.8 | 6029.3 |
| 4823.4 | 0.2 | 1550.4 | 6373.8 | 3962.1 |
| 5168 | 0.3 | 1033.6 | 6201.6 | 3962.1 |

Appendix C.3. Continued…

| Bandwidth  (Hz) | Duration (s) | Minimum Frequency  (Hz) | Maximum Frequency  (Hz) | Peak Frequency  (Hz) |
| --- | --- | --- | --- | --- |
| 6029.3 | 0.3 | 861.3 | 6890.6 | 3962.1 |
| 7924.2 | 0.3 | 689.1 | 8613.3 | 4134.4 |
| 6029.3 | 0.3 | 689.1 | 6718.4 | 3789.8 |
| 7062.9 | 0.3 | 689.1 | 7752 | 3962.1 |
| 7924.2 | 0.3 | 689.1 | 8613.3 | 3445.3 |
| 7579.7 | 0.4 | 861.3 | 8441 | 2067.2 |
| 6201.6 | 0.2 | 861.3 | 7062.9 | 3100.8 |
| 7752 | 0.4 | 689.1 | 8441 | 3789.8 |
| 6029.3 | 0.3 | 689.1 | 6718.4 | 3789.8 |
| 6029.3 | 0.1 | 689.1 | 6718.4 | 2928.5 |
| 4651.2 | 0.2 | 1378.1 | 6029.3 | 3789.8 |
| 4134.4 | 0.2 | 1378.1 | 5512.5 | 2067.2 |
| 2928.5 | 0.1 | 1894.9 | 4823.4 | 3100.8 |
| 5340.2 | 0.3 | 861.3 | 6201.6 | 1722.7 |
| 5684.8 | 0.3 | 689.1 | 6373.8 | 3445.3 |
| 5340.2 | 0.2 | 1033.6 | 6373.8 | 3789.8 |
| 4823.4 | 0.2 | 689.1 | 5512.5 | 2756.2 |
| 7235.2 | 0.5 | 689.1 | 7924.2 | 3445.3 |
| 7235.2 | 0.3 | 1033.6 | 8268.8 | 4306.6 |
| 7579.7 | 0.2 | 1033.6 | 8613.3 | 4134.4 |
| 6546.1 | 0.3 | 861.3 | 7407.4 | 3789.8 |
| 6890.6 | 0.1 | 689.1 | 7579.7 | 3100.8 |
| 5857 | 0.2 | 2928.5 | 8785.5 | 3617.6 |
| 5857 | 0.3 | 861.3 | 6718.4 | 3273 |
| 5512.5 | 0.3 | 1378.1 | 6890.6 | 3789.8 |
| 4995.7 | 0.3 | 1550.4 | 6546.1 | 3273 |
| 4823.4 | 0.3 | 1378.1 | 6201.6 | 4134.4 |
| 4823.4 | 0.2 | 1205.9 | 6029.3 | 3445.3 |
| 5168 | 0.1 | 861.3 | 6029.3 | 3100.8 |
| 4995.7 | 0.2 | 1033.6 | 6029.3 | 4306.6 |
| 5857 | 0.2 | 861.3 | 6718.4 | 3617.6 |
| 4823.4 | 0.3 | 1722.7 | 6546.1 | 3445.3 |

Appendix C.3. Continued…

| Bandwidth  (Hz) | Duration (s) | Minimum Frequency  (Hz) | Maximum Frequency  (Hz) | Peak Frequency  (Hz) |
| --- | --- | --- | --- | --- |
| 5168 | 0.2 | 1378.1 | 6546.1 | 4478.9 |
| 3962.1 | 0.4 | 1378.1 | 5340.2 | 3445.3 |
| 4478.9 | 0.3 | 1550.4 | 6029.3 | 4478.9 |
| 4134.4 | 0.1 | 1205.9 | 5340.2 | 3445.3 |
| 5512.5 | 0.2 | 1033.6 | 6546.1 | 2584 |
| 5168 | 0.2 | 861.3 | 6029.3 | 3789.8 |
| 8096.5 | 0.3 | 689.1 | 8785.5 | 3789.8 |
| 6890.6 | 0.2 | 1205.9 | 8096.5 | 3789.8 |
| 4995.7 | 0.3 | 1722.7 | 6718.4 | 4651.2 |
| 3962.1 | 0.2 | 2239.5 | 6201.6 | 3445.3 |
| 4995.7 | 0.2 | 1205.9 | 6201.6 | 3445.3 |
| 3962.1 | 0.2 | 2239.5 | 6201.6 | 3445.3 |

Appendix C.4. Measurements of the vocal characters for *P. samarensis.*

| Bandwidth  (Hz) | Duration (s) | Minimum Frequency  (Hz) | Maximum Frequency  (Hz) | Peak Frequency  (Hz) |
| --- | --- | --- | --- | --- |
| 4995.7 | 0.1 | 689.1 | 5684.8 | 4306.6 |
| 4306.6 | 0.1 | 1378.1 | 5684.8 | 4134.4 |
| 4134.4 | 0.1 | 1550.4 | 5684.8 | 4306.6 |
| 4478.9 | 0.2 | 1722.7 | 6201.6 | 4134.4 |
| 4651.2 | 0.2 | 1722.7 | 6373.8 | 4134.4 |
| 3100.8 | 0.1 | 2756.2 | 5857 | 3962.1 |
| 3273 | 0.1 | 2411.7 | 5684.8 | 4134.4 |
| 3445.3 | 0.1 | 1894.9 | 5340.2 | 3789.8 |
| 2928.5 | 0.1 | 3445.3 | 6373.8 | 3962.1 |
| 2067.2 | 0 | 3617.6 | 5684.8 | 4134.4 |
| 2928.5 | 0 | 3617.6 | 6546.1 | 4306.6 |
| 1722.7 | 0 | 3445.3 | 5168 | 4134.4 |
| 3617.6 | 0.1 | 2584 | 6201.6 | 4306.6 |
| 3617.6 | 0.1 | 2584 | 6201.6 | 4306.6 |
| 3789.8 | 0.1 | 2411.7 | 6201.6 | 4134.4 |
| 2928.5 | 0.1 | 2411.7 | 5340.2 | 4995.7 |
| 4823.4 | 0.1 | 689.1 | 5512.5 | 3962.1 |
| 4478.9 | 0.1 | 1722.7 | 6201.6 | 4995.7 |
| 3445.3 | 0.1 | 1894.9 | 5340.2 | 4134.4 |
| 4306.6 | 0.1 | 861.3 | 5168 | 3617.6 |
| 6201.6 | 0.2 | 689.1 | 6890.6 | 3962.1 |
| 5684.8 | 0.1 | 344.5 | 6029.3 | 344.5 |
| 3273 | 0.2 | 1722.7 | 4995.7 | 3789.8 |
| 2411.7 | 0.1 | 2239.5 | 4651.2 | 3789.8 |
| 3617.6 | 0.1 | 2239.5 | 5857 | 3962.1 |
| 3445.3 | 0.2 | 2239.5 | 5684.8 | 3962.1 |
| 4134.4 | 0.2 | 1722.7 | 5857 | 3445.3 |
| 4651.2 | 0.2 | 1550.4 | 6201.6 | 3962.1 |
| 5512.5 | 0.2 | 1205.9 | 6718.4 | 3962.1 |
| 4478.9 | 0.2 | 1550.4 | 6029.3 | 3273 |
| 5684.8 | 0.2 | 689.1 | 6373.8 | 1722.7 |
| 4478.9 | 0.2 | 1550.4 | 6029.3 | 1722.7 |
| 4651.2 | 0.2 | 1550.4 | 6201.6 | 3962.1 |
| 4823.4 | 0.2 | 1550.4 | 6373.8 | 3789.8 |
| Appendix C.4. Continued… | | |  |  |
| Bandwidth  (Hz) | **Duration (s)** | **Minimum Frequency**  **(Hz)** | **Maximum Frequency**  **(Hz)** | **Peak Frequency**  **(Hz)** |
| 4478.9 | 0.2 | 1550.4 | 6029.3 | 3445.3 |
| 4306.6 | 0.1 | 1722.7 | 6029.3 | 3789.8 |
| 4134.4 | 0.1 | 1550.4 | 5684.8 | 3962.1 |
| 4478.9 | 0.2 | 1722.7 | 6201.6 | 3789.8 |
| 4478.9 | 0.2 | 1722.7 | 6201.6 | 3962.1 |
| 4306.6 | 0.2 | 1550.4 | 5857 | 3100.8 |
| 4651.2 | 0.2 | 1550.4 | 6201.6 | 3789.8 |
| 4134.4 | 0.2 | 1722.7 | 5857 | 3789.8 |
| 4306.6 | 0.2 | 1550.4 | 5857 | 5168 |
| 4306.6 | 0.1 | 1722.7 | 6029.3 | 3617.6 |
| 4306.6 | 0.2 | 1722.7 | 6029.3 | 3445.3 |
| 4306.6 | 0.1 | 1722.7 | 6029.3 | 3617.6 |
| 3617.6 | 0.3 | 2584 | 6201.6 | 3789.8 |
| 5684.8 | 0.5 | 344.5 | 6029.3 | 3789.8 |
| 4134.4 | 0.2 | 2239.5 | 6373.8 | 5168 |
| 3962.1 | 0.3 | 2411.7 | 6373.8 | 4134.4 |
| 4478.9 | 0.3 | 1894.9 | 6373.8 | 3789.8 |
| 4651.2 | 0.5 | 1550.4 | 6201.6 | 4995.7 |
| 4478.9 | 1 | 1722.7 | 6201.6 | 3789.8 |
| 4478.9 | 2.3 | 1894.9 | 6373.8 | 3789.8 |
| 4306.6 | 0.4 | 1894.9 | 6201.6 | 3789.8 |
| 3962.1 | 0.3 | 2239.5 | 6201.6 | 3617.6 |
| 4306.6 | 0.3 | 1894.9 | 6201.6 | 4478.9 |
| 4651.2 | 0.3 | 1722.7 | 6373.8 | 3789.8 |
| 4478.9 | 0.3 | 1722.7 | 6201.6 | 4306.6 |
| 4823.4 | 0.2 | 1722.7 | 6546.1 | 3617.6 |
| 5512.5 | 0.3 | 689.1 | 6201.6 | 3445.3 |
| 4651.2 | 0.5 | 1722.7 | 6373.8 | 3962.1 |
| 3617.6 | 0.4 | 2584 | 6201.6 | 3962.1 |
| 4134.4 | 0.4 | 2067.2 | 6201.6 | 3445.3 |
| 3962.1 | 0.3 | 2067.2 | 6029.3 | 3789.8 |
| 3445.3 | 0.2 | 2584 | 6029.3 | 3789.8 |
| 3617.6 | 0.2 | 2239.5 | 5857 | 4306.6 |
| 3445.3 | 0.8 | 2584 | 6029.3 | 4478.9 |
| 3789.8 | 0.5 | 2411.7 | 6201.6 | 3789.8 |

Appendix C.4. Continued…

| Bandwidth  (Hz) | Duration (s) | Minimum Frequency  (Hz) | Maximum Frequency  (Hz) | Peak Frequency  (Hz) |
| --- | --- | --- | --- | --- |
| 3962.1 | 0.4 | 2239.5 | 6201.6 | 3617.6 |
| 4306.6 | 0.3 | 1722.7 | 6029.3 | 3445.3 |
| 3789.8 | 0.5 | 2411.7 | 6201.6 | 3789.8 |
| 3962.1 | 0.3 | 2411.7 | 6373.8 | 3789.8 |

Appendix C.5. Measurements of the vocal characters for *R. leucocephalus.*

| Bandwidth  (Hz) | Duration (s) | Minimum Frequency  (Hz) | Maximum Frequency  (Hz) | Peak Frequency  (Hz) |
| --- | --- | --- | --- | --- |
| 3445.3 | 0.2 | 689.1 | 4134.4 | 3273 |
| 3617.6 | 0.1 | 344.5 | 3962.1 | 2928.5 |
| 4995.7 | 0.1 | 689.1 | 5684.8 | 3445.3 |
| 3445.3 | 0.1 | 516.8 | 3962.1 | 3273 |
| 3617.6 | 0.1 | 344.5 | 3962.1 | 3100.8 |
| 3617.6 | 0.1 | 344.5 | 3962.1 | 3100.8 |
| 3445.3 | 0.1 | 516.8 | 3962.1 | 3445.3 |
| 3617.6 | 0.5 | 344.5 | 3962.1 | 689.1 |
| 4306.6 | 0.3 | 344.5 | 4651.2 | 861.3 |
| 4995.7 | 0.3 | 689.1 | 5684.8 | 3445.3 |
| 4478.9 | 0.2 | 344.5 | 4823.4 | 2756.2 |
| 3789.8 | 0.3 | 689.1 | 4478.9 | 1033.6 |
| 3445.3 | 0.4 | 516.8 | 3962.1 | 861.3 |
| 3617.6 | 0.1 | 689.1 | 4306.6 | 3273 |
| 2928.5 | 0.1 | 689.1 | 3617.6 | 3100.8 |
| 3100.8 | 0.1 | 689.1 | 3789.8 | 861.3 |
| 2928.5 | 0.1 | 516.8 | 3445.3 | 1033.6 |
| 3273 | 0.2 | 344.5 | 3617.6 | 1033.6 |
| 3789.8 | 0.3 | 344.5 | 4134.4 | 1033.6 |
| 4306.6 | 0.3 | 344.5 | 4651.2 | 689.1 |
| 4134.4 | 0.3 | 344.5 | 4478.9 | 2928.5 |
| 3789.8 | 0.3 | 344.5 | 4134.4 | 1033.6 |
| 4306.6 | 0.4 | 344.5 | 4651.2 | 3445.3 |
| 3789.8 | 0.3 | 344.5 | 4134.4 | 2928.5 |
| 4478.9 | 0.3 | 344.5 | 4823.4 | 1033.6 |
| 3273 | 0.3 | 689.1 | 3962.1 | 1033.6 |
| 3789.8 | 0.2 | 689.1 | 4478.9 | 3100.8 |
| 4134.4 | 0.3 | 516.8 | 4651.2 | 689.1 |
| 4134.4 | 0.3 | 344.5 | 4478.9 | 3273 |
| 3617.6 | 0.3 | 516.8 | 4134.4 | 1033.6 |
| 4306.6 | 0.3 | 344.5 | 4651.2 | 3273 |
| 3789.8 | 0.4 | 689.1 | 4478.9 | 3445.3 |
| 4134.4 | 0.5 | 689.1 | 4823.4 | 3273 |

Appendix C.5. Continued…

| Bandwidth  (Hz) | Duration (s) | Minimum Frequency  (Hz) | Maximum Frequency  (Hz) | Peak Frequency  (Hz) |
| --- | --- | --- | --- | --- |
| 3789.8 | 0.4 | 689.1 | 4478.9 | 3273 |
| 3445.3 | 0.2 | 516.8 | 3962.1 | 1033.6 |
| 3789.8 | 0.2 | 516.8 | 4306.6 | 1033.6 |
| 3617.6 | 0.2 | 689.1 | 4306.6 | 1033.6 |
| 3445.3 | 0.3 | 689.1 | 4134.4 | 1033.6 |
| 3962.1 | 0.2 | 516.8 | 4478.9 | 1033.6 |
| 2756.2 | 0.2 | 172.3 | 2928.5 | 689.1 |
| 2067.2 | 0.2 | 172.3 | 2239.5 | 344.5 |
| 3445.3 | 0.3 | 861.3 | 4306.6 | 2067.2 |
| 3100.8 | 0.4 | 516.8 | 3617.6 | 689.1 |
| 4134.4 | 0.5 | 516.8 | 4651.2 | 3445.3 |
| 2928.5 | 0.4 | 1722.7 | 4651.2 | 3617.6 |
| 4134.4 | 0.4 | 1033.6 | 5168 | 3617.6 |
| 3617.6 | 0.3 | 1378.1 | 4995.7 | 3789.8 |
| 4478.9 | 0.4 | 1033.6 | 5512.5 | 3617.6 |
| 4995.7 | 0.4 | 1033.6 | 6029.3 | 3617.6 |
| 4823.4 | 0.4 | 1205.9 | 6029.3 | 3617.6 |
| 3617.6 | 0.3 | 1033.6 | 4651.2 | 1722.7 |
| 2928.5 | 0.3 | 1378.1 | 4306.6 | 3445.3 |
| 4995.7 | 0.5 | 1033.6 | 6029.3 | 3617.6 |
| 3617.6 | 0.5 | 1378.1 | 4995.7 | 3445.3 |
| 3617.6 | 0.4 | 1205.9 | 4823.4 | 3445.3 |
| 4823.4 | 0.4 | 1205.9 | 6029.3 | 2756.2 |
| 3962.1 | 0.4 | 1205.9 | 5168 | 3617.6 |
| 3789.8 | 0.4 | 689.1 | 4478.9 | 3617.6 |
| 3789.8 | 0.4 | 689.1 | 4478.9 | 3617.6 |
| 5684.8 | 0.4 | 1033.6 | 6718.4 | 2584 |
| 5684.8 | 0.4 | 861.3 | 6546.1 | 3273 |
| 6029.3 | 0.5 | 689.1 | 6718.4 | 3789.8 |
| 5512.5 | 0.4 | 1378.1 | 6890.6 | 3445.3 |
| 3962.1 | 0.3 | 1033.6 | 4995.7 | 2584 |
| 3617.6 | 0.5 | 689.1 | 4306.6 | 3445.3 |

| Bandwidth  (Hz) | Duration (s) | Minimum Frequency  (Hz) | Maximum Frequency  (Hz) | Peak Frequency  (Hz) |
| --- | --- | --- | --- | --- |
| 3445.3 | 0.4 | 689.1 | 4134.4 | 2756.2 |
| 3273 | 0.5 | 1378.1 | 4651.2 | 2756.2 |
| 3273 | 0.5 | 1550.4 | 4823.4 | 3617.6 |
| 2928.5 | 0.4 | 1722.7 | 4651.2 | 2756.2 |
| 3877.22 | 0.31 | 729.01 | 4606.23 | 2486.61 |
| 737.18 | 0.12 | 380.81 | 856.07 | 1154.25 |
| 4478.9 | 0.1 | 861.3 | 5340.2 | 5168 |
| 4651.2 | 0.1 | 689.1 | 5340.2 | 4995.7 |
| 4478.9 | 0.1 | 861.3 | 5340.2 | 5168 |
| 4306.6 | 0.1 | 861.3 | 5168 | 1033.6 |
| 4134.4 | 0.1 | 1205.9 | 5340.2 | 5168 |
| 4306.6 | 0.1 | 516.8 | 4823.4 | 1033.6 |
| 4651.2 | 0.1 | 689.1 | 5340.2 | 4995.7 |
| 4823.4 | 0.1 | 516.8 | 5340.2 | 1894.9 |

Appendix C.5. Continued…

Appendix C.6. Measurements of the vocal characters for *R. waldeni.*

| Bandwidth  (Hz) | Duration (s) | Minimum Frequency  (Hz) | Maximum Frequency  (Hz) | Peak Frequency  (Hz) |
| --- | --- | --- | --- | --- |
| 4995.7 | 0.2 | 689.1 | 5684.8 | 1033.6 |
| 4995.7 | 0.3 | 689.1 | 5684.8 | 689.1 |
| 4823.4 | 0.2 | 689.1 | 5512.5 | 689.1 |
| 4651.2 | 0.2 | 689.1 | 5340.2 | 3962.1 |
| 4478.9 | 0.2 | 689.1 | 5168 | 3789.8 |
| 4823.4 | 0.2 | 689.1 | 5512.5 | 4823.4 |
| 4651.2 | 0.2 | 689.1 | 5340.2 | 3962.1 |
| 4651.2 | 0.2 | 689.1 | 5340.2 | 4134.4 |
| 4651.2 | 0.3 | 689.1 | 5340.2 | 4306.6 |
| 5168 | 0.3 | 516.8 | 5684.8 | 689.1 |
| 3789.8 | 0.1 | 516.8 | 4306.6 | 1033.6 |
| 3789.8 | 0.1 | 516.8 | 4306.6 | 861.3 |
| 3617.6 | 0.1 | 689.1 | 4306.6 | 1033.6 |
| 3789.8 | 0.1 | 516.8 | 4306.6 | 861.3 |
| 3789.8 | 0.2 | 516.8 | 4306.6 | 1033.6 |
| 3962.1 | 0.3 | 516.8 | 4478.9 | 4306.6 |
| 3789.8 | 0.2 | 516.8 | 4306.6 | 861.3 |
| 3789.8 | 0.2 | 689.1 | 4478.9 | 861.3 |
| 3789.8 | 0.2 | 689.1 | 4478.9 | 1033.6 |
| 3789.8 | 0.2 | 689.1 | 4478.9 | 861.3 |
| 3789.8 | 0.1 | 516.8 | 4306.6 | 861.3 |

Appendix C.7. Measurements of the vocal characters for *R. plicatus.*

| Bandwidth  (Hz) | Duration (s) | Minimum Frequency  (Hz) | Maximum Frequency  (Hz) | Peak Frequency  (Hz) |
| --- | --- | --- | --- | --- |
| 3273 | 0.4 | 172.3 | 3445.3 | 516.8 |
| 3445.3 | 1.1 | 172.3 | 3617.6 | 1205.9 |
| 3100.8 | 0.6 | 172.3 | 3273 | 1033.6 |
| 3100.8 | 0.4 | 172.3 | 3273 | 1205.9 |
| 4995.7 | 0.3 | 172.3 | 5168 | 1205.9 |
| 3445.3 | 1.6 | 172.3 | 3617.6 | 516.8 |
| 3100.8 | 0 | 344.5 | 3445.3 | 516.8 |
| 2756.2 | 0.2 | 344.5 | 3100.8 | 1205.9 |
| 1894.9 | 0.9 | 0 | 1894.9 | 172.3 |
| 1894.9 | 1 | 0 | 1894.9 | 172.3 |
| 2239.5 | 0.5 | 344.5 | 2584 | 1205.9 |
| 3100.8 | 0.6 | 344.5 | 3445.3 | 1205.9 |
| 3789.8 | 0.4 | 172.3 | 3962.1 | 516.8 |
| 1894.9 | 0.4 | 344.5 | 2239.5 | 1205.9 |
| 3100.8 | 0.5 | 344.5 | 3445.3 | 516.8 |
| 2928.5 | 0.5 | 344.5 | 3273 | 1205.9 |
|  |  |  |  |  |

Appendix C.8. Measurements of the vocal characters for *B. h. hydrocorax*

| Bandwidth  (Hz) | Duration (s) | Minimum Frequency  (Hz) | Maximum Frequency  (Hz) | Peak Frequency  (Hz) |
| --- | --- | --- | --- | --- |
| 2411.7 | 0.2 | 516.8 | 2928.5 | 861.3 |
| 2584 | 0.2 | 516.8 | 3100.8 | 861.3 |
| 2584 | 0.2 | 516.8 | 3100.8 | 861.3 |
| 2756.2 | 0.2 | 344.5 | 3100.8 | 861.3 |
| 2756.2 | 0.3 | 344.5 | 3100.8 | 861.3 |
| 2584 | 0.2 | 516.8 | 3100.8 | 861.3 |
| 2756.2 | 0.2 | 344.5 | 3100.8 | 689.1 |
| 2928.5 | 0.2 | 516.8 | 3445.3 | 861.3 |
| 1894.9 | 0.2 | 516.8 | 2411.7 | 689.1 |
| 2928.5 | 0.2 | 516.8 | 3445.3 | 689.1 |
| 2411.7 | 0.3 | 689.1 | 3100.8 | 861.3 |
| 1894.9 | 0.3 | 689.1 | 2584 | 861.3 |
| 2928.5 | 0.3 | 689.1 | 3617.6 | 861.3 |
| 1722.7 | 0.2 | 516.8 | 2239.5 | 861.3 |
| 1722.7 | 0.3 | 516.8 | 2239.5 | 861.3 |
| 1894.9 | 0.3 | 516.8 | 2411.7 | 861.3 |
| 2756.2 | 0.3 | 516.8 | 3273 | 861.3 |
| 1722.7 | 0.2 | 344.5 | 2067.2 | 861.3 |
| 2067.2 | 0.3 | 344.5 | 2411.7 | 861.3 |
| 2067.2 | 0.3 | 344.5 | 2411.7 | 861.3 |
| 2067.2 | 0.4 | 516.8 | 2584 | 689.1 |
| 3617.6 | 0.4 | 344.5 | 3962.1 | 861.3 |
| 2239.5 | 0.2 | 516.8 | 2756.2 | 861.3 |
| 861.3 | 0.2 | 516.8 | 1378.1 | 861.3 |
| 344.5 | 0.2 | 689.1 | 1033.6 | 861.3 |
| 1894.9 | 0.2 | 516.8 | 2411.7 | 689.1 |
| 1894.9 | 0.2 | 516.8 | 2411.7 | 861.3 |
| 2067.2 | 0.2 | 516.8 | 2584 | 689.1 |
| 1205.9 | 0.2 | 516.8 | 1722.7 | 861.3 |
| 344.5 | 0.2 | 689.1 | 1033.6 | 861.3 |
| 1378.1 | 0.2 | 516.8 | 1894.9 | 861.3 |
| 1722.7 | 0.3 | 516.8 | 2239.5 | 861.3 |
| 2239.5 | 0.4 | 344.5 | 2584 | 861.3 |

Appendix C.8. Continued…

| Bandwidth  (Hz) | Duration (s) | Minimum Frequency  (Hz) | Maximum Frequency  (Hz) | Peak Frequency  (Hz) |
| --- | --- | --- | --- | --- |
| 2239.5 | 0.6 | 344.5 | 2584 | 689.1 |
| 3100.8 | 0.8 | 344.5 | 3445.3 | 861.3 |
| 1894.9 | 0.3 | 689.1 | 2584 | 861.3 |
| 2584 | 0.3 | 689.1 | 3273 | 861.3 |
| 1894.9 | 0.3 | 689.1 | 2584 | 861.3 |
| 2584 | 0.3 | 344.5 | 2928.5 | 861.3 |
| 2756.2 | 0.2 | 516.8 | 3273 | 861.3 |
| 2067.2 | 0.3 | 516.8 | 2584 | 861.3 |
| 2584 | 0.3 | 516.8 | 3100.8 | 689.1 |
| 2756.2 | 0.2 | 516.8 | 3273 | 689.1 |
| 2756.2 | 0.3 | 516.8 | 3273 | 1033.6 |
| 2756.2 | 1.1 | 344.5 | 3100.8 | 1033.6 |
| 2584 | 0.2 | 516.8 | 3100.8 | 689.1 |
| 2756.2 | 0.3 | 516.8 | 3273 | 689.1 |
| 1378.1 | 0.2 | 516.8 | 1894.9 | 689.1 |
| 1378.1 | 0.3 | 689.1 | 2067.2 | 861.3 |
| 1722.7 | 0.4 | 516.8 | 2239.5 | 689.1 |

Appendix C.9. Measurements of the vocal characters for *B. h. semigaleatus.*

| Bandwidth  (Hz) | Duration (s) | Minimum Frequency  (Hz) | Maximum Frequency  (Hz) | Peak Frequency  (Hz) |
| --- | --- | --- | --- | --- |
| 4651.2 | 0.4 | 861.3 | 5512.5 | 1033.6 |
| 4651.2 | 0.7 | 861.3 | 5512.5 | 5168 |
| 4651.2 | 0.5 | 861.3 | 5512.5 | 5340.2 |
| 4651.2 | 0.8 | 861.3 | 5512.5 | 2584 |
| 4651.2 | 0.3 | 689.1 | 5340.2 | 861.3 |
| 4823.4 | 0.7 | 689.1 | 5512.5 | 5168 |
| 4823.4 | 0.4 | 689.1 | 5512.5 | 4478.9 |
| 4823.4 | 0.7 | 861.3 | 5684.8 | 5168 |
| 4823.4 | 0.7 | 689.1 | 5512.5 | 5340.2 |
| 4651.2 | 1 | 861.3 | 5512.5 | 5340.2 |
| 4651.2 | 0.3 | 861.3 | 5512.5 | 1033.6 |
| 4651.2 | 0.4 | 861.3 | 5512.5 | 1033.6 |
| 4478.9 | 0.4 | 861.3 | 5340.2 | 1033.6 |
| 4651.2 | 0.5 | 689.1 | 5340.2 | 1033.6 |
| 4823.4 | 0.7 | 861.3 | 5684.8 | 5168 |
| 4651.2 | 0.7 | 861.3 | 5512.5 | 5340.2 |
| 4478.9 | 0.7 | 861.3 | 5340.2 | 2756.2 |
| 4478.9 | 0.7 | 861.3 | 5340.2 | 3273 |
| 4478.9 | 0.5 | 1033.6 | 5512.5 | 1033.6 |
| 4651.2 | 0.4 | 861.3 | 5512.5 | 5340.2 |
| 4651.2 | 0.4 | 861.3 | 5512.5 | 5340.2 |
| 4823.4 | 1.2 | 516.8 | 5340.2 | 689.1 |
| 4823.4 | 0.6 | 516.8 | 5340.2 | 689.1 |
| 4651.2 | 0.3 | 689.1 | 5340.2 | 861.3 |
| 4651.2 | 0.3 | 689.1 | 5340.2 | 689.1 |
| 4651.2 | 0.3 | 861.3 | 5512.5 | 5340.2 |

Appendix C.10. Measurements of the vocal characters for *B. h. mindanensis.*

| Bandwidth  (Hz) | Duration (s) | Minimum Frequency  (Hz) | Maximum Frequency  (Hz) | Peak Frequency  (Hz) |
| --- | --- | --- | --- | --- |
| 1894.9 | 0.3 | 689.1 | 2584 | 861.3 |
| 1894.9 | 0.3 | 689.1 | 2584 | 861.3 |
| 689.1 | 0.3 | 516.8 | 1205.9 | 861.3 |
| 1550.4 | 0.3 | 516.8 | 2067.2 | 861.3 |
| 1205.9 | 0.3 | 516.8 | 1722.7 | 1033.6 |
| 1722.7 | 0.3 | 516.8 | 2239.5 | 1033.6 |
| 2411.7 | 0.3 | 344.5 | 2756.2 | 861.3 |
| 2239.5 | 0.3 | 344.5 | 2584 | 861.3 |
| 2411.7 | 0.3 | 344.5 | 2756.2 | 689.1 |
| 2928.5 | 0.3 | 344.5 | 3273 | 689.1 |
| 3273 | 0.3 | 344.5 | 3617.6 | 689.1 |
| 1722.7 | 0.3 | 516.8 | 2239.5 | 861.3 |
| 1722.7 | 0.3 | 516.8 | 2239.5 | 1033.6 |
| 1894.9 | 0.3 | 516.8 | 2411.7 | 1033.6 |
| 1894.9 | 0.3 | 516.8 | 2411.7 | 861.3 |
| 516.8 | 0.2 | 689.1 | 1205.9 | 1033.6 |
| 516.8 | 0.3 | 689.1 | 1205.9 | 1033.6 |
| 1033.6 | 0.3 | 689.1 | 1722.7 | 1033.6 |
| 2239.5 | 0.2 | 689.1 | 2928.5 | 861.3 |
| 2239.5 | 0.3 | 689.1 | 2928.5 | 1033.6 |
| 2584 | 0.3 | 689.1 | 3273 | 1033.6 |
| 3445.3 | 0.3 | 689.1 | 4134.4 | 1033.6 |
| 3617.6 | 0.3 | 689.1 | 4306.6 | 1033.6 |
| 2584 | 0.2 | 689.1 | 3273 | 1033.6 |
| 3273 | 0.3 | 516.8 | 3789.8 | 861.3 |
| 2411.7 | 0.3 | 516.8 | 2928.5 | 861.3 |
| 3445.3 | 0.3 | 516.8 | 3962.1 | 861.3 |
| 1550.4 | 0.2 | 689.1 | 2239.5 | 861.3 |
| 1550.4 | 0.3 | 689.1 | 2239.5 | 1033.6 |
| 1550.4 | 0.3 | 689.1 | 2239.5 | 1033.6 |
| 1894.9 | 0.3 | 516.8 | 2411.7 | 1033.6 |
| 2067.2 | 0.3 | 344.5 | 2411.7 | 689.1 |
| 2928.5 | 0.3 | 344.5 | 3273 | 689.1 |

Appendix C.10. Continued…

| Bandwidth  (Hz) | Duration (s) | Minimum Frequency  (Hz) | Maximum Frequency  (Hz) | Peak Frequency  (Hz) |
| --- | --- | --- | --- | --- |
| 2239.5 | 0.3 | 344.5 | 2584 | 689.1 |
| 1722.7 | 0.3 | 516.8 | 2239.5 | 861.3 |
| 3273 | 0.3 | 516.8 | 3789.8 | 689.1 |
| 2584 | 0.3 | 516.8 | 3100.8 | 1033.6 |
| 2756.2 | 0.2 | 516.8 | 3273 | 689.1 |
| 2756.2 | 0.3 | 516.8 | 3273 | 861.3 |
| 2239.5 | 0.3 | 516.8 | 2756.2 | 1033.6 |
| 2756.2 | 0.3 | 516.8 | 3273 | 861.3 |
| 2584 | 0.3 | 516.8 | 3100.8 | 1033.6 |
| 2067.2 | 0.3 | 689.1 | 2756.2 | 861.3 |
| 2411.7 | 0.3 | 689.1 | 3100.8 | 1033.6 |
| 1894.9 | 0.3 | 689.1 | 2584 | 861.3 |
| 3273 | 0.3 | 516.8 | 3789.8 | 1894.9 |
| 2411.7 | 0.2 | 689.1 | 3100.8 | 1894.9 |
| 2756.2 | 0.2 | 516.8 | 3273 | 2067.2 |
| 3273 | 0.3 | 516.8 | 3789.8 | 689.1 |
| 2928.5 | 0.2 | 516.8 | 3445.3 | 1894.9 |
| 2411.7 | 0.2 | 689.1 | 3100.8 | 861.3 |
| 3100.8 | 0.2 | 516.8 | 3617.6 | 689.1 |
| 3273 | 0.3 | 516.8 | 3789.8 | 1894.9 |
| 2067.2 | 0.3 | 516.8 | 2584 | 861.3 |
| 2067.2 | 0.3 | 516.8 | 2584 | 861.3 |
| 2584 | 0.3 | 516.8 | 3100.8 | 689.1 |
| 2584 | 0.3 | 516.8 | 3100.8 | 689.1 |
| 2584 | 0.3 | 516.8 | 3100.8 | 689.1 |
| 3100.8 | 0.2 | 689.1 | 3789.8 | 1033.6 |
| 2928.5 | 0.2 | 689.1 | 3617.6 | 1033.6 |
| 2928.5 | 0.2 | 689.1 | 3617.6 | 1033.6 |
| 2411.7 | 0.3 | 516.8 | 2928.5 | 1033.6 |
| 2584 | 0.3 | 516.8 | 3100.8 | 1033.6 |
| 2067.2 | 0.2 | 516.8 | 2584 | 1033.6 |
| 2239.5 | 0.2 | 516.8 | 2756.2 | 1033.6 |

Appendix C.10. Continued…

| Bandwidth  (Hz) | Duration (s) | Minimum Frequency  (Hz) | Maximum Frequency  (Hz) | Peak Frequency  (Hz) |
| --- | --- | --- | --- | --- |
| 2239.5 | 0.2 | 516.8 | 2756.2 | 1033.6 |
| 3445.3 | 0.3 | 516.8 | 3962.1 | 2584 |
| 3445.3 | 0.3 | 516.8 | 3962.1 | 2584 |
| 3445.3 | 0.3 | 516.8 | 3962.1 | 2584 |
| 3100.8 | 0.2 | 689.1 | 3789.8 | 1033.6 |
| 3100.8 | 0.2 | 689.1 | 3789.8 | 1033.6 |
| 3100.8 | 0.2 | 689.1 | 3789.8 | 1033.6 |
| 3617.6 | 0.3 | 344.5 | 3962.1 | 861.3 |
| 4306.6 | 0.3 | 516.8 | 4823.4 | 1033.6 |
| 3100.8 | 0.2 | 344.5 | 3445.3 | 689.1 |
| 3100.8 | 0.2 | 344.5 | 3445.3 | 689.1 |
| 3100.8 | 0.2 | 344.5 | 3445.3 | 689.1 |
| 2928.5 | 0.3 | 516.8 | 3445.3 | 1033.6 |
| 2239.5 | 0.2 | 516.8 | 2756.2 | 861.3 |
| 2239.5 | 0.2 | 516.8 | 2756.2 | 861.3 |
| 4478.9 | 0.3 | 516.8 | 4995.7 | 1033.6 |
| 3962.1 | 0.3 | 516.8 | 4478.9 | 861.3 |
| 3962.1 | 0.3 | 344.5 | 4306.6 | 689.1 |
| 3273 | 0.4 | 344.5 | 3617.6 | 689.1 |
| 2584 | 0.3 | 344.5 | 2928.5 | 689.1 |
| 1894.9 | 0.3 | 344.5 | 2239.5 | 861.3 |
| 2239.5 | 0.4 | 344.5 | 2584 | 689.1 |
| 3273 | 0.2 | 344.5 | 3617.6 | 689.1 |
| 3273 | 0.3 | 344.5 | 3617.6 | 861.3 |
| 3445.3 | 0.2 | 516.8 | 3962.1 | 861.3 |
| 3100.8 | 0.2 | 344.5 | 3445.3 | 861.3 |
| 3273 | 0.2 | 344.5 | 3617.6 | 689.1 |
| 2756.2 | 0.2 | 516.8 | 3273 | 689.1 |
| 2584 | 0.2 | 344.5 | 2928.5 | 516.8 |
| 2067.2 | 0.2 | 344.5 | 2411.7 | 689.1 |
| 2239.5 | 0.3 | 344.5 | 2584 | 689.1 |

Appendix C.10. Continued…

| Bandwidth  (Hz) | Duration (s) | Minimum Frequency  (Hz) | Maximum Frequency  (Hz) | Peak Frequency  (Hz) |
| --- | --- | --- | --- | --- |
| 2067.2 | 0.2 | 344.5 | 2411.7 | 689.1 |
| 2239.5 | 0.2 | 344.5 | 2584 | 689.1 |
| 2239.5 | 0.3 | 344.5 | 2584 | 689.1 |
| 1550.4 | 0.1 | 344.5 | 1894.9 | 689.1 |
| 2239.5 | 0.2 | 344.5 | 2584 | 689.1 |
| 2584 | 0.2 | 516.8 | 3100.8 | 689.1 |
| 3445.3 | 0.3 | 516.8 | 3962.1 | 689.1 |
| 2239.5 | 0.3 | 516.8 | 2756.2 | 689.1 |
| 2928.5 | 0.3 | 516.8 | 3445.3 | 689.1 |
| 2411.7 | 0.2 | 344.5 | 2756.2 | 689.1 |
| 2584 | 0.2 | 344.5 | 2928.5 | 689.1 |
| 2756.2 | 0.2 | 344.5 | 3100.8 | 689.1 |
| 3273 | 0.3 | 344.5 | 3617.6 | 689.1 |
| 3100.8 | 0.3 | 344.5 | 3445.3 | 689.1 |
| 3100.8 | 0.3 | 344.5 | 3445.3 | 689.1 |
| 7752 | 0.3 | 344.5 | 8096.5 | 689.1 |
| 2928.5 | 0.3 | 344.5 | 3273 | 689.1 |
| 2928.5 | 0.3 | 344.5 | 3273 | 689.1 |
| 2584 | 0.3 | 344.5 | 2928.5 | 689.1 |
| 2239.5 | 0.3 | 344.5 | 2584 | 689.1 |
| 2067.2 | 0.3 | 344.5 | 2411.7 | 689.1 |
| 2411.7 | 0.3 | 344.5 | 2756.2 | 689.1 |
| 2239.5 | 0.3 | 344.5 | 2584 | 689.1 |
| 3100.8 | 0.3 | 344.5 | 3445.3 | 689.1 |
| 2584 | 0.3 | 344.5 | 2928.5 | 689.1 |
| 2928.5 | 0.3 | 344.5 | 3273 | 1205.9 |
| 2928.5 | 0.3 | 344.5 | 3273 | 1205.9 |
| 2928.5 | 0.3 | 344.5 | 3273 | 1205.9 |
| 2928.5 | 0.2 | 172.3 | 3100.8 | 689.1 |
| 2756.2 | 0.3 | 344.5 | 3100.8 | 689.1 |
| 2584 | 0.3 | 344.5 | 2928.5 | 689.1 |
| 2067.2 | 0.3 | 344.5 | 2411.7 | 689.1 |
| 1722.7 | 0.3 | 516.8 | 2239.5 | 861.3 |
| 1722.7 | 0.3 | 344.5 | 2067.2 | 861.3 |

Appendix C.10. Continued…

| Bandwidth  (Hz) | Duration (s) | Minimum Frequency  (Hz) | Maximum Frequency  (Hz) | Peak Frequency  (Hz) |
| --- | --- | --- | --- | --- |
| 2067.2 | 0.4 | 344.5 | 2411.7 | 861.3 |
| 1722.7 | 0.3 | 344.5 | 2067.2 | 861.3 |
| 2067.2 | 0.3 | 344.5 | 2411.7 | 861.3 |
| 1722.7 | 0.3 | 516.8 | 2239.5 | 861.3 |
| 1722.7 | 0.3 | 344.5 | 2067.2 | 861.3 |
| 2067.2 | 0.4 | 344.5 | 2411.7 | 861.3 |
| 2067.2 | 0.3 | 344.5 | 2411.7 | 861.3 |
| 1894.9 | 0.2 | 516.8 | 2411.7 | 689.1 |
| 1722.7 | 0.2 | 516.8 | 2239.5 | 689.1 |
| 1894.9 | 0.3 | 516.8 | 2411.7 | 689.1 |
| 1894.9 | 0.3 | 516.8 | 2411.7 | 689.1 |
| 1894.9 | 0.3 | 516.8 | 2411.7 | 861.3 |
| 1205.9 | 0.3 | 516.8 | 1722.7 | 861.3 |
| 689.1 | 0.3 | 516.8 | 1205.9 | 861.3 |
| 689.1 | 0.3 | 516.8 | 1205.9 | 861.3 |
| 1033.6 | 0.3 | 516.8 | 1550.4 | 861.3 |
| 1378.1 | 0.3 | 516.8 | 1894.9 | 861.3 |
| 1550.4 | 0.3 | 516.8 | 2067.2 | 861.3 |
| 1378.1 | 0.3 | 516.8 | 1894.9 | 689.1 |
| 1550.4 | 0.2 | 516.8 | 2067.2 | 689.1 |
| 1550.4 | 0.2 | 516.8 | 2067.2 | 689.1 |
| 1550.4 | 0.3 | 516.8 | 2067.2 | 861.3 |
| 1378.1 | 0.4 | 516.8 | 1894.9 | 689.1 |
| 1378.1 | 0.3 | 516.8 | 1894.9 | 689.1 |
| 1550.4 | 0.3 | 516.8 | 2067.2 | 689.1 |
| 1378.1 | 0.3 | 516.8 | 1894.9 | 861.3 |
| 3617.6 | 0.2 | 516.8 | 4134.4 | 689.1 |
| 3789.8 | 0.3 | 516.8 | 4306.6 | 689.1 |
| 3617.6 | 0.2 | 516.8 | 4134.4 | 861.3 |
| 1378.1 | 0.3 | 516.8 | 1894.9 | 861.3 |
| 1378.1 | 0.3 | 516.8 | 1894.9 | 861.3 |
| 2411.7 | 0.1 | 516.8 | 2928.5 | 861.3 |
| 2584 | 0.3 | 516.8 | 3100.8 | 1033.6 |

Appendix C.10. Continued…

| Bandwidth  (Hz) | Duration (s) | Minimum Frequency  (Hz) | Maximum Frequency  (Hz) | Peak Frequency  (Hz) |
| --- | --- | --- | --- | --- |
| 1378.1 | 0.2 | 689.1 | 2067.2 | 861.3 |
| 1378.1 | 0.3 | 516.8 | 1894.9 | 861.3 |
| 1205.9 | 0.3 | 689.1 | 1894.9 | 861.3 |
| 2411.7 | 0.2 | 516.8 | 2928.5 | 861.3 |
| 2584 | 0.3 | 516.8 | 3100.8 | 861.3 |
| 2411.7 | 0.3 | 516.8 | 2928.5 | 861.3 |
| 2584 | 0.3 | 516.8 | 3100.8 | 861.3 |
| 2067.2 | 0.2 | 689.1 | 2756.2 | 861.3 |
| 2411.7 | 0.2 | 516.8 | 2928.5 | 861.3 |
| 1894.9 | 0.2 | 516.8 | 2411.7 | 861.3 |
| 2067.2 | 0.3 | 516.8 | 2584 | 861.3 |
| 4134.4 | 0.3 | 516.8 | 4651.2 | 1033.6 |
| 3789.8 | 0.3 | 516.8 | 4306.6 | 1033.6 |
| 3789.8 | 0.3 | 516.8 | 4306.6 | 1033.6 |
| 3789.8 | 0.2 | 516.8 | 4306.6 | 1033.6 |
| 4478.9 | 0.3 | 689.1 | 5168 | 1033.6 |
| 4478.9 | 0.3 | 689.1 | 5168 | 1033.6 |
| 3789.8 | 0.3 | 516.8 | 4306.6 | 861.3 |
| 3445.3 | 0.3 | 689.1 | 4134.4 | 1033.6 |
| 2239.5 | 0.2 | 689.1 | 2928.5 | 861.3 |
| 2411.7 | 0.2 | 689.1 | 3100.8 | 1033.6 |
| 2584 | 0.2 | 689.1 | 3273 | 1033.6 |
| 2584 | 0.2 | 689.1 | 3273 | 1033.6 |
| 1894.9 | 0.2 | 689.1 | 2584 | 1033.6 |
| 1894.9 | 0.3 | 689.1 | 2584 | 1033.6 |
| 2928.5 | 0.2 | 689.1 | 3617.6 | 861.3 |
| 2756.2 | 0.3 | 689.1 | 3445.3 | 1033.6 |
| 2756.2 | 0.3 | 689.1 | 3445.3 | 1033.6 |
| 2928.5 | 0.3 | 516.8 | 3445.3 | 2411.7 |
| 2928.5 | 0.3 | 689.1 | 3617.6 | 2584 |
| 2756.2 | 0.3 | 861.3 | 3617.6 | 1205.9 |
| 2756.2 | 0.3 | 861.3 | 3617.6 | 1033.6 |
