# Supplementary material for: Description and comparison of Philippine hornbill (Bucerotidae) vocalizations
Source: Biodivers Data J. 2019 Nov 13;7:e31723. doi: 10.3897/BDJ.7.e31723 (PMC6868047; doi:10.3897/BDJ.7.e31723)
Supplement: Supplementary material 2 — Spectrogram [file bdj-07-e31723-s002.pdf]

APPENDIX B

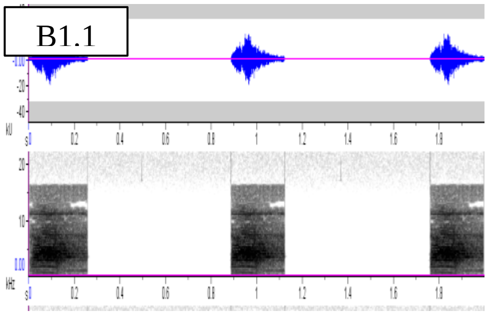
B1.1

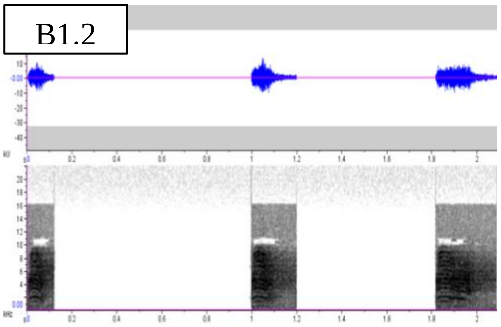
B1.2

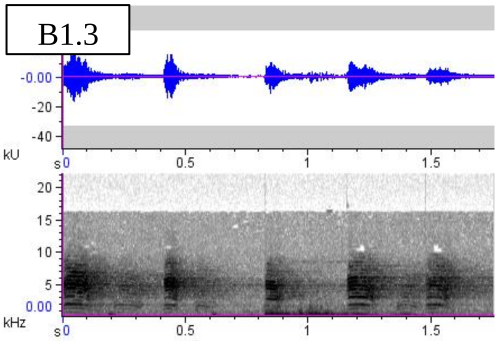
B1.3

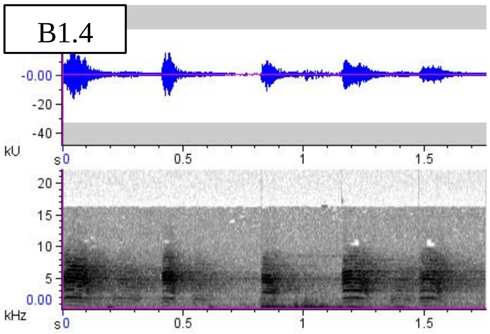
B1.4

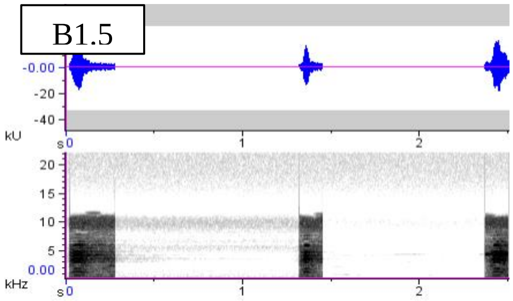
B1.5

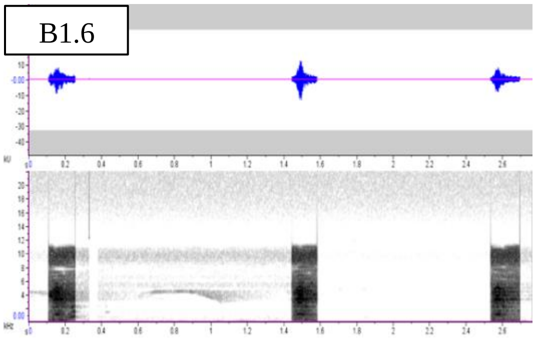
B1.6

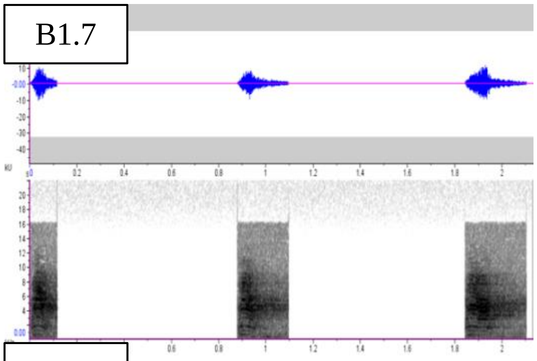
B1.7

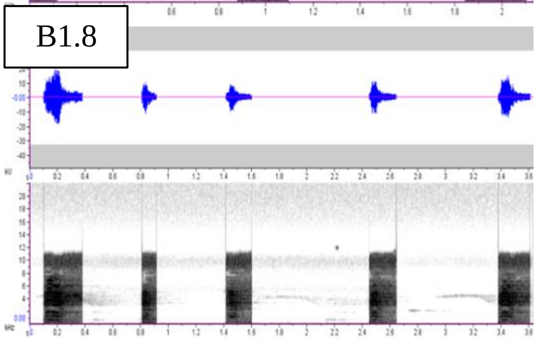
B1.8

Appendix B.1. Waveform and Spectrogram of *P. affinis*.

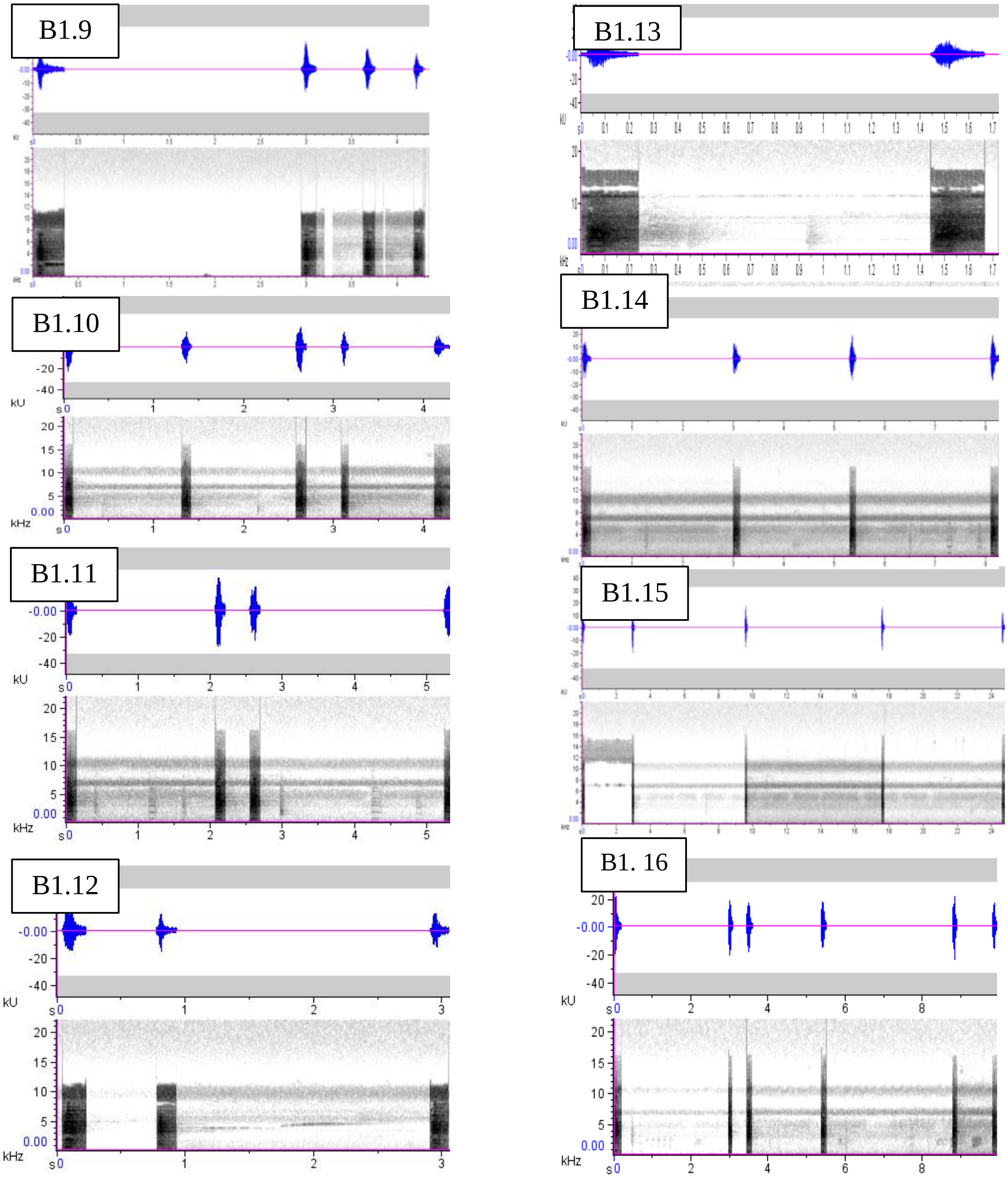

Appendix B.1. Continued…

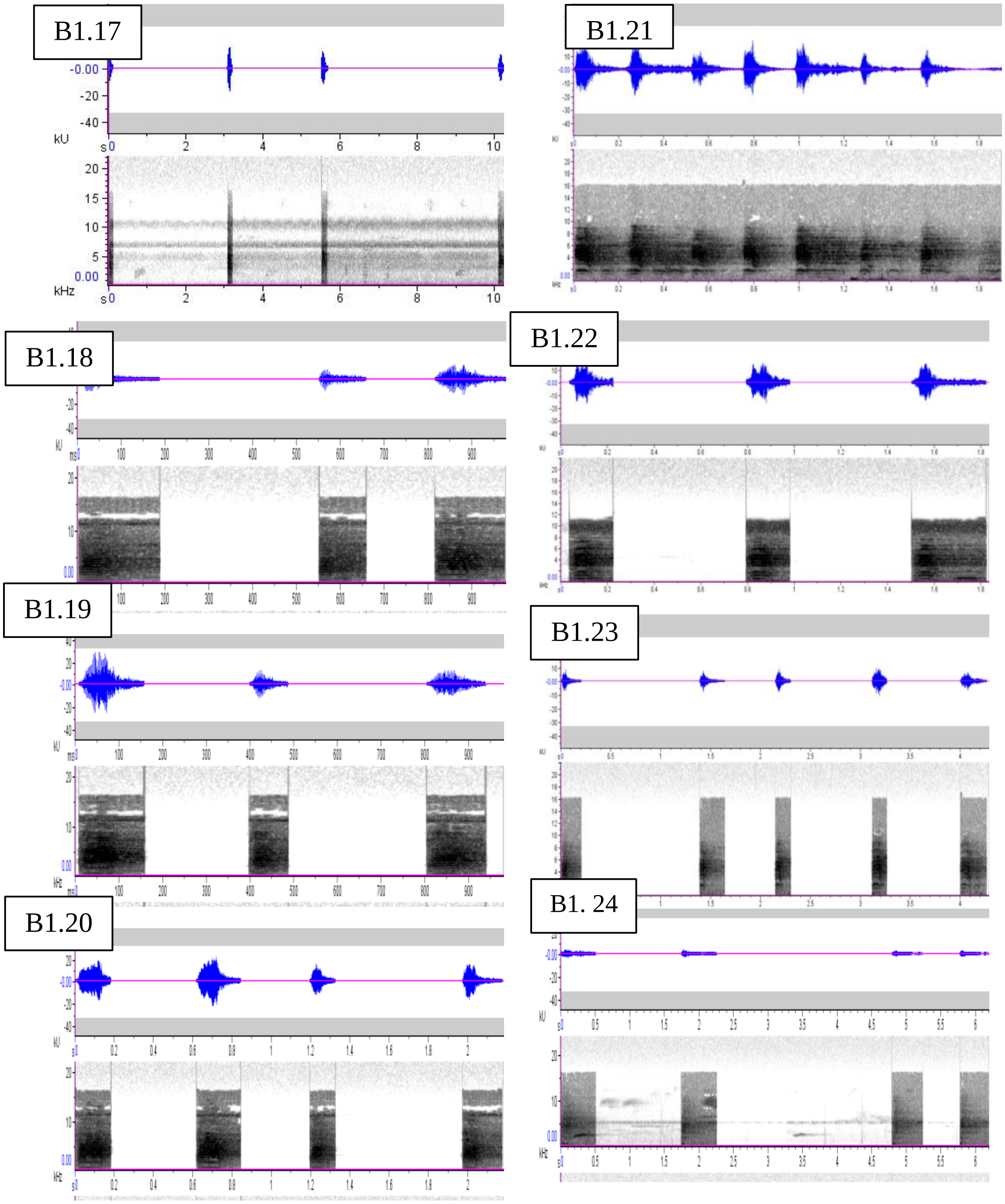

Appendix B.1. Continued…

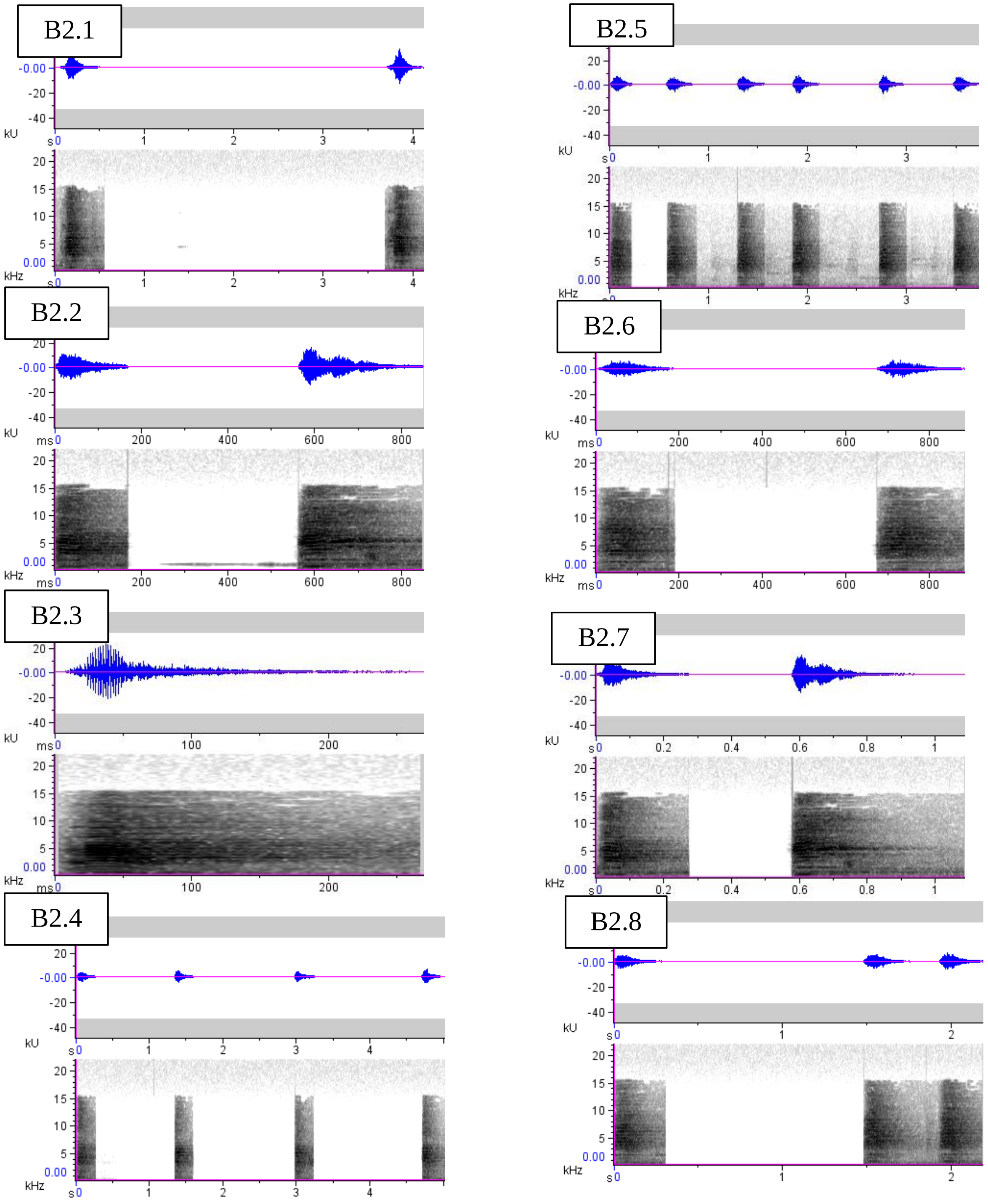

Appendix B.2. Waveform and Spectrogram of *P. manillae*.

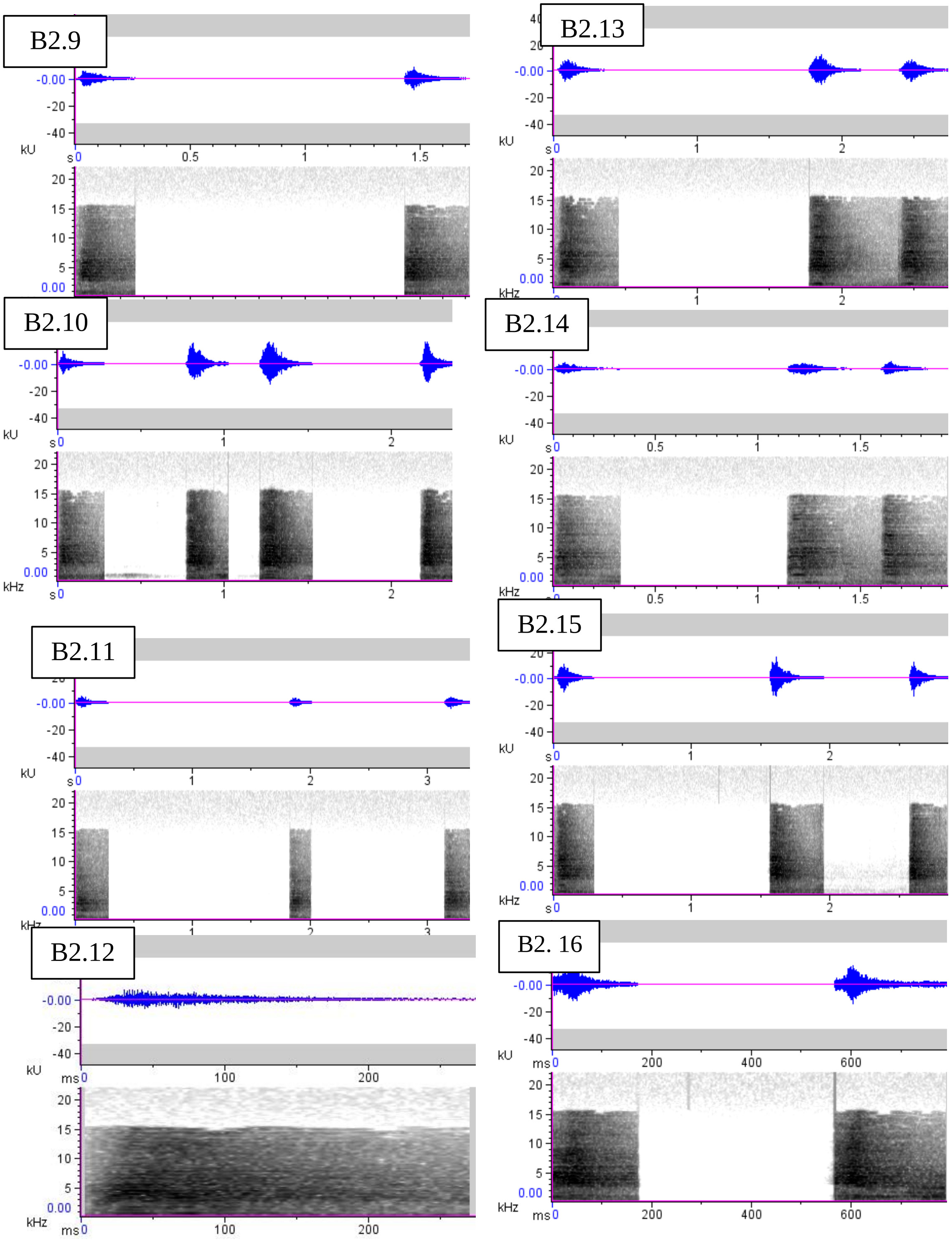

Appendix B.2. Continued…

B2.17

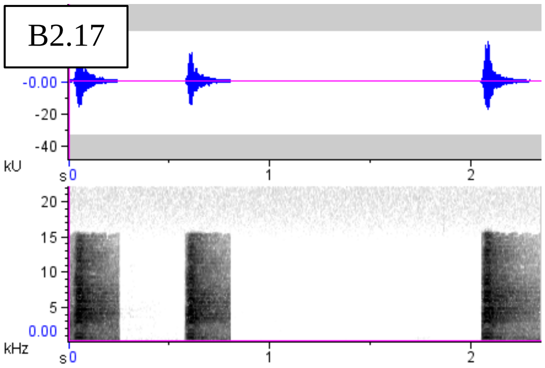

B2.18

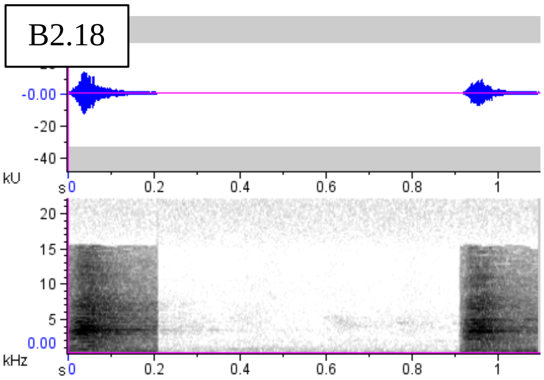

B2.19

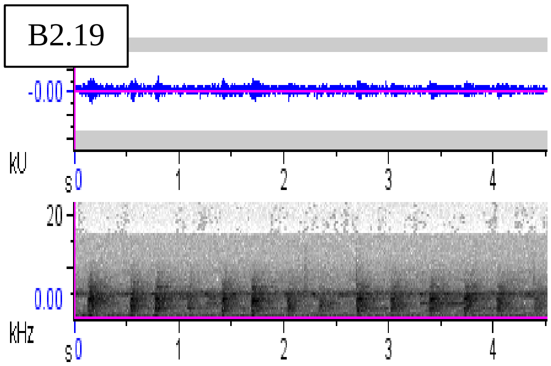

B2.20

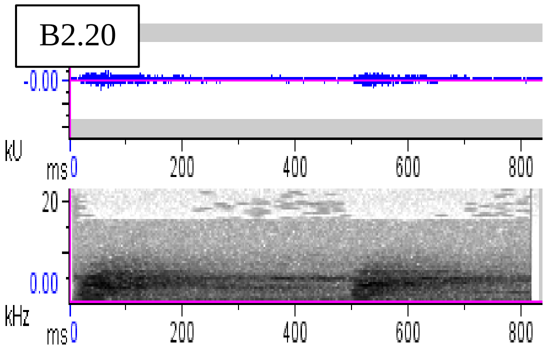

B2.21

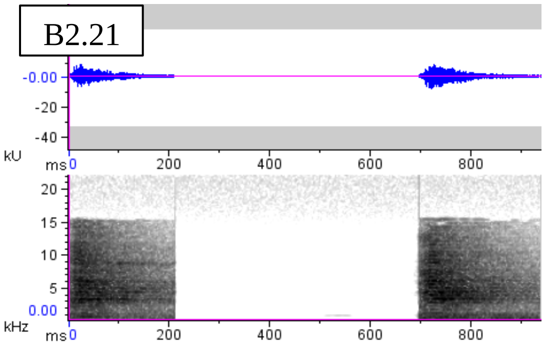

B2.22

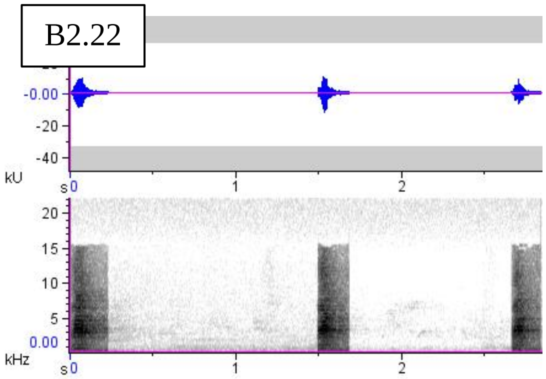

B2.23

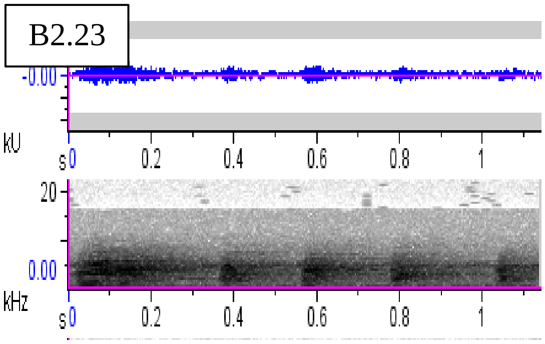

B2. 24

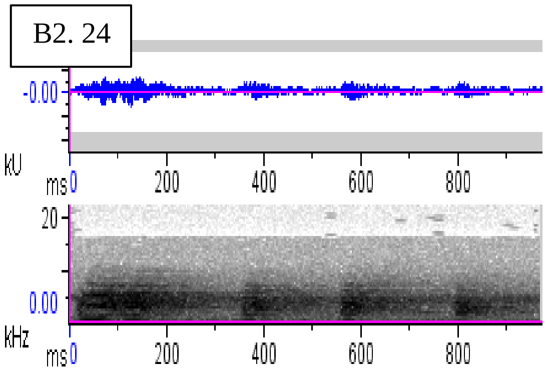

Appendix B.2. Continued…

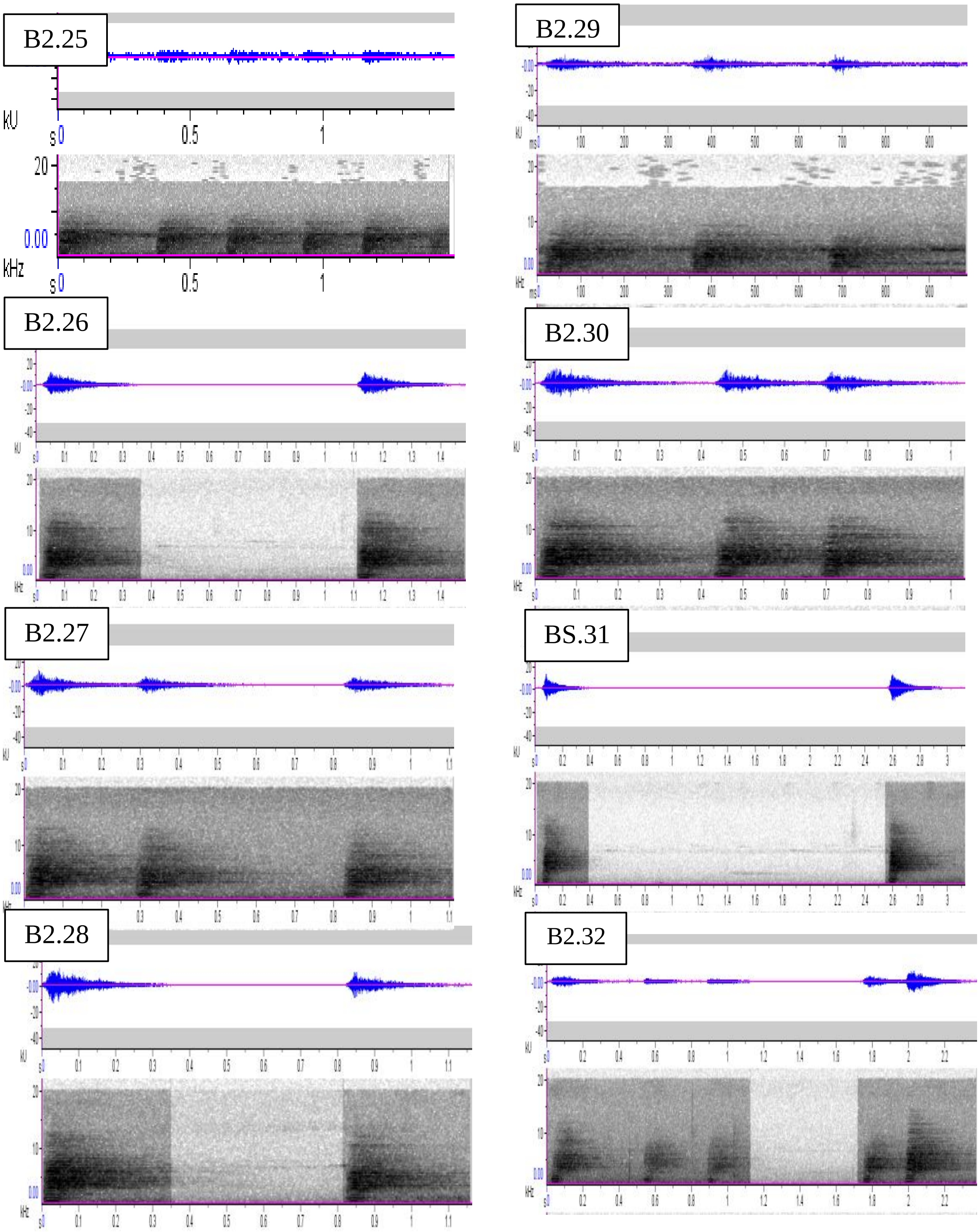

Appendix B.2. Continued…

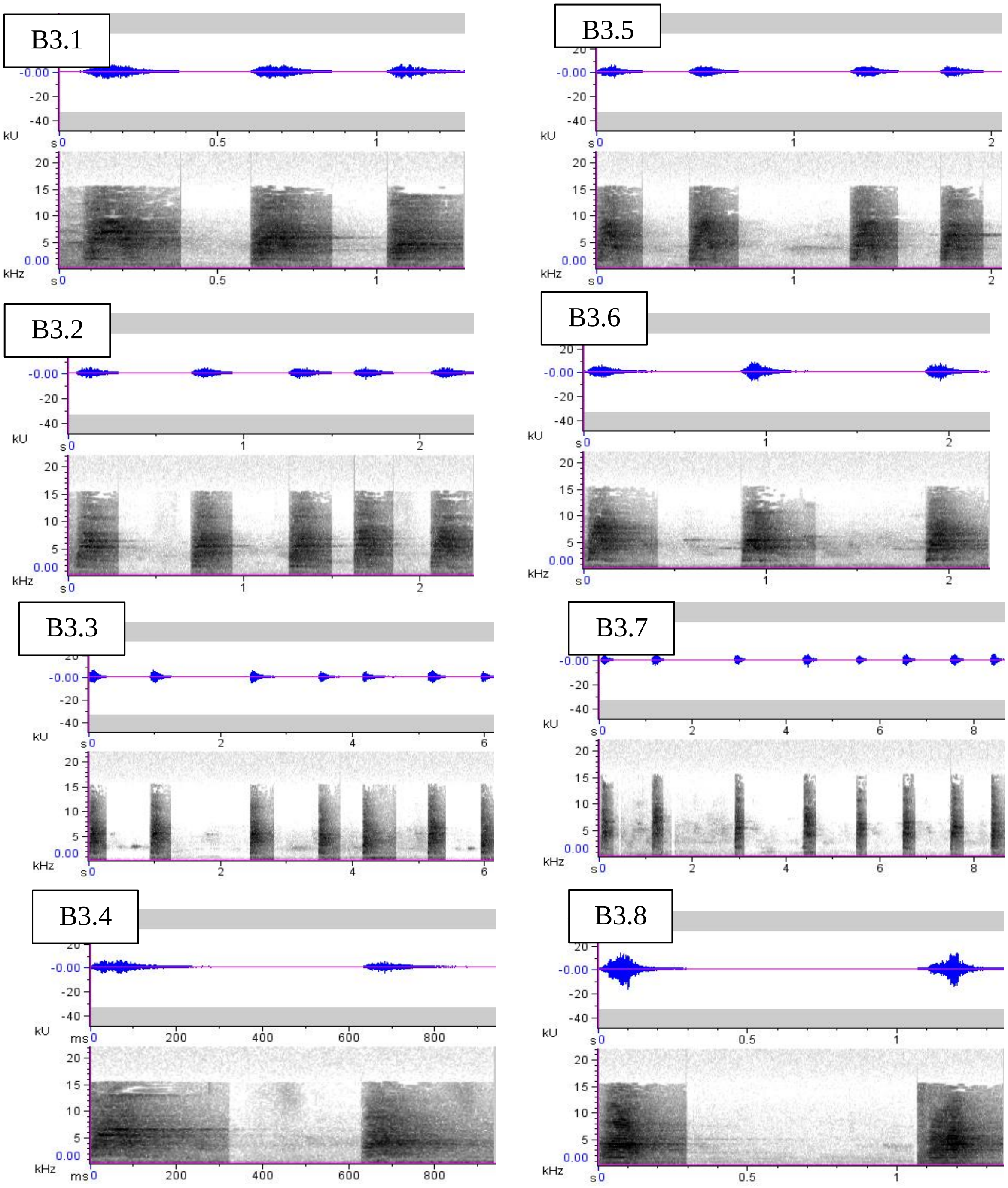

Appendix B.3. Waveform and Spectrogram of *P. panini.*

B3.9

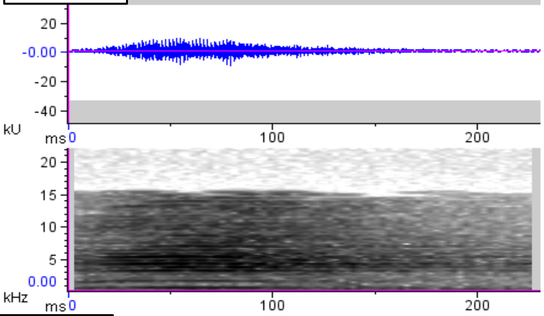

B3.10

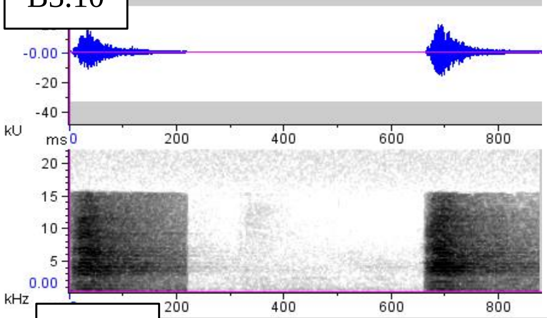

B3.11

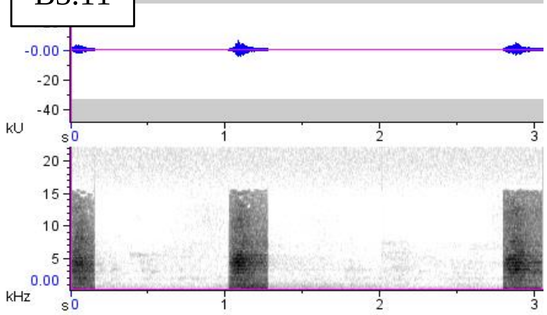

B3.12

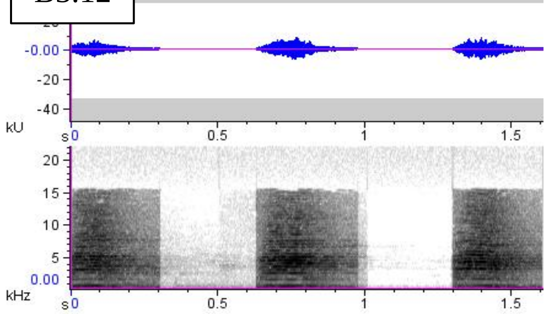

B3.13

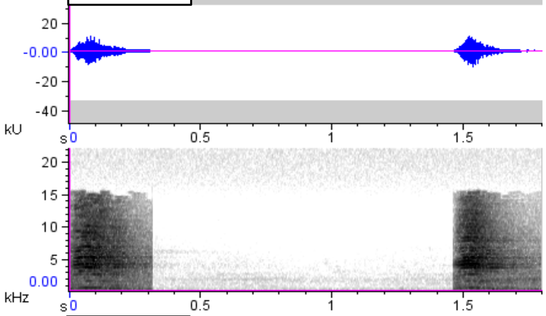

B3.14

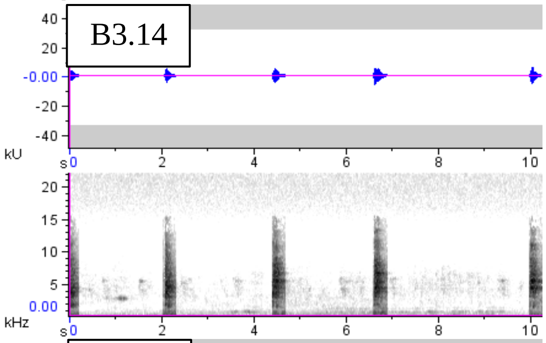

B3.15

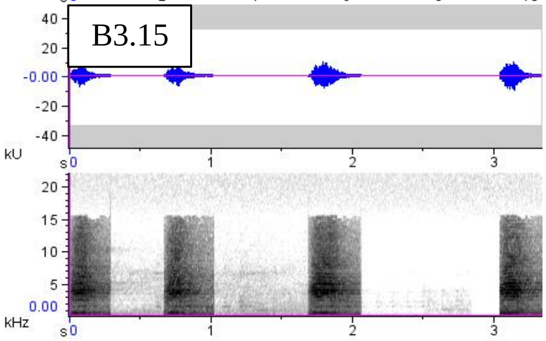

B3. 16

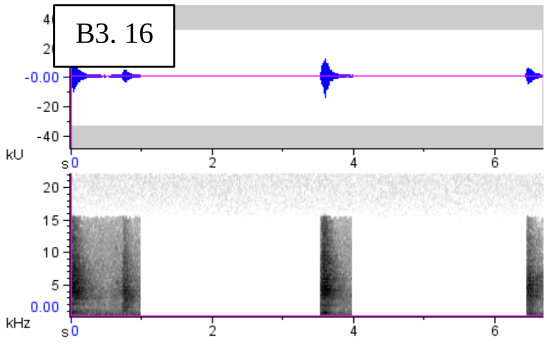

Appendix B.3. Continued…

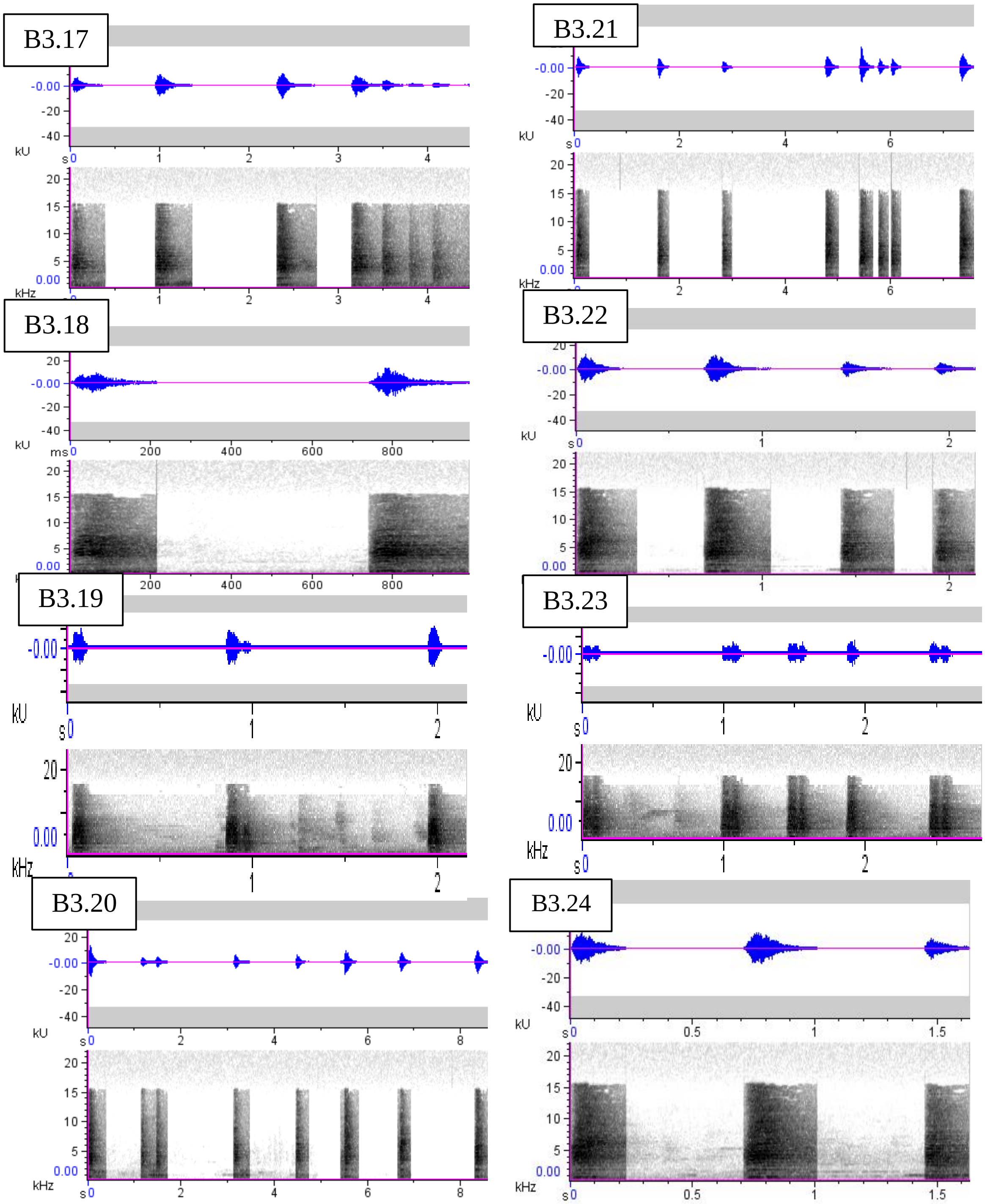

Appendix B.3. Continued…

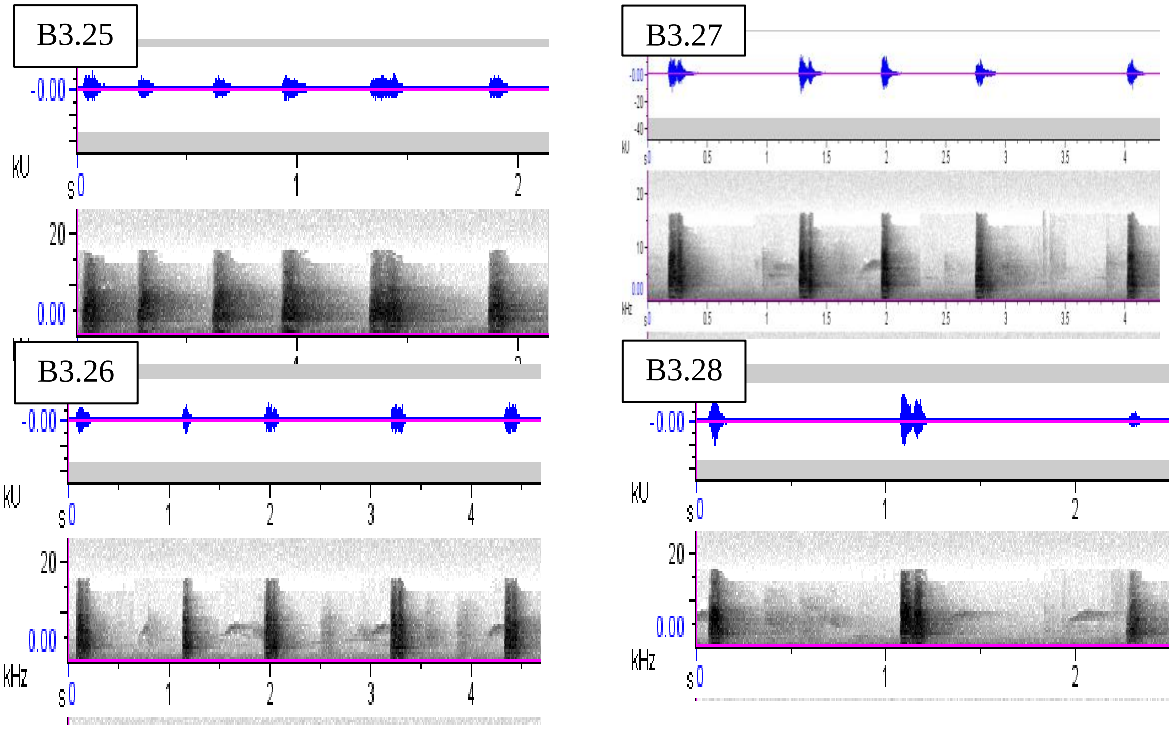

Appendix B.3. Continued…

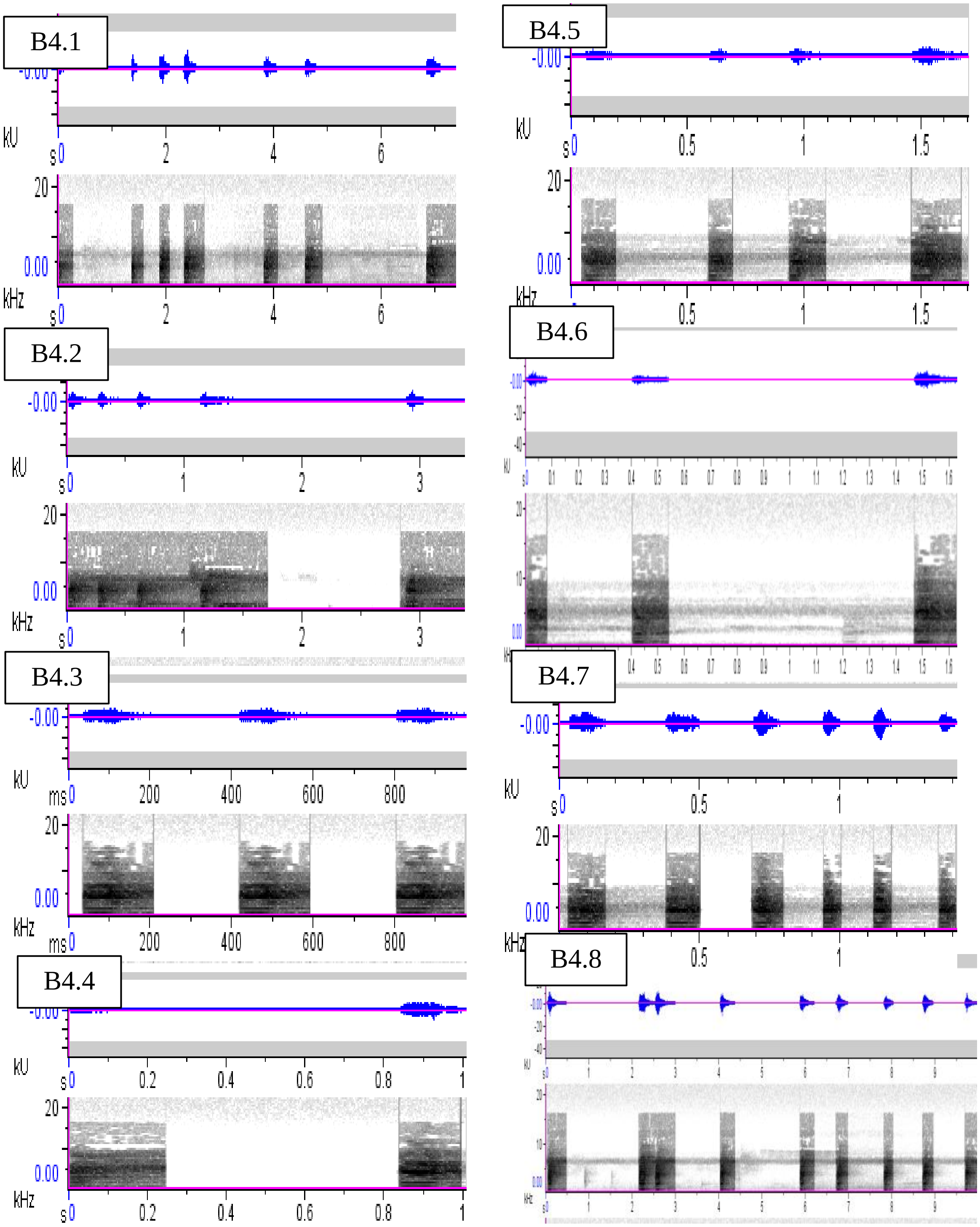

Appendix B.4. Waveform and Spectrogram of *P. samarensis*.

B4.9

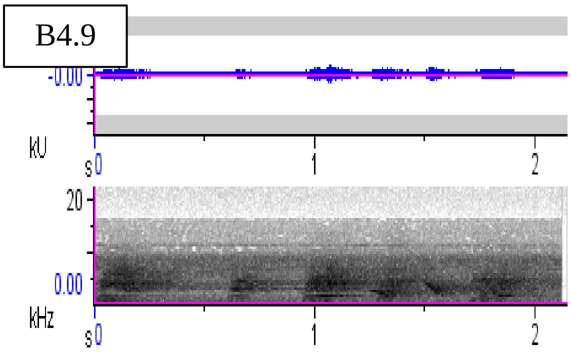

B4.10

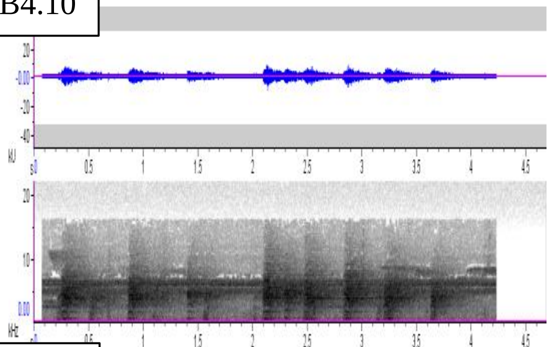

B4.11

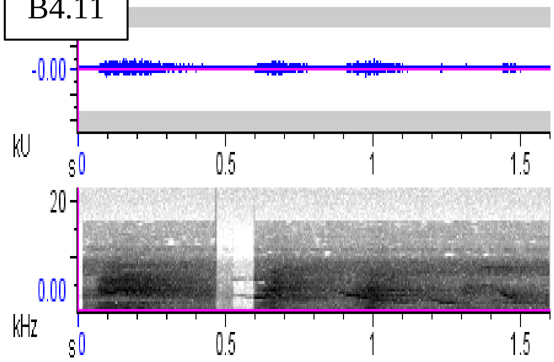

B4.12

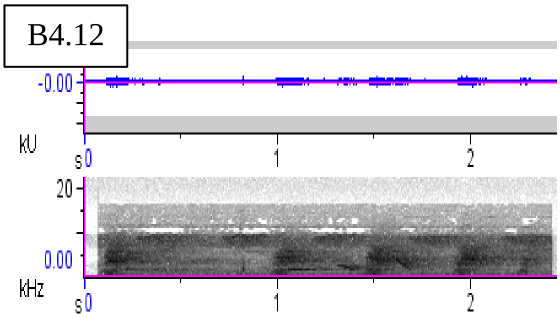

B4.13

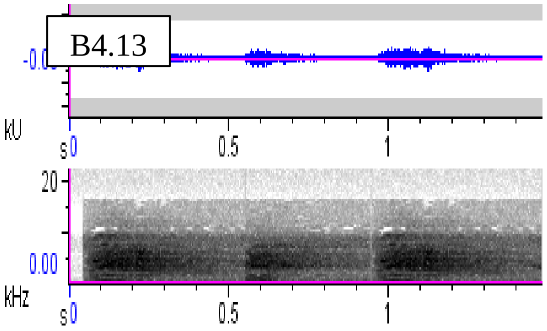

B4.14

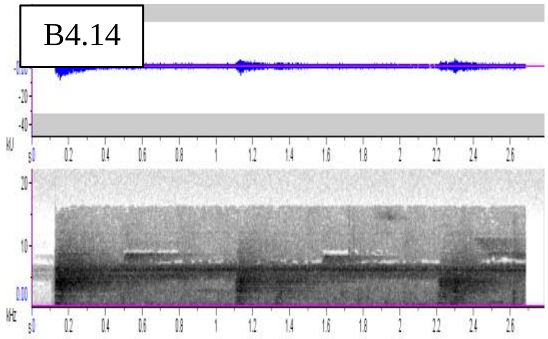

B4.15

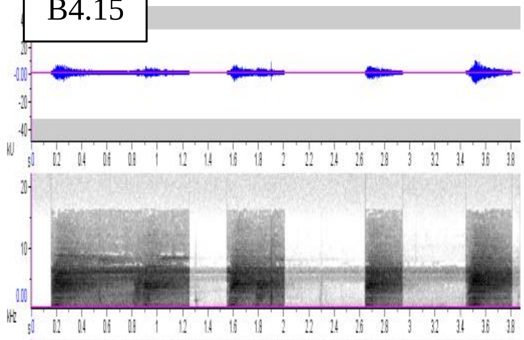

B4. 16

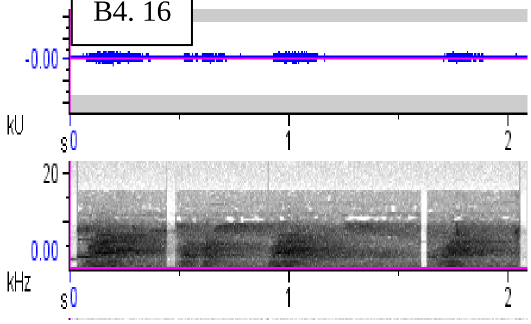

Appendix B.4. Continued…

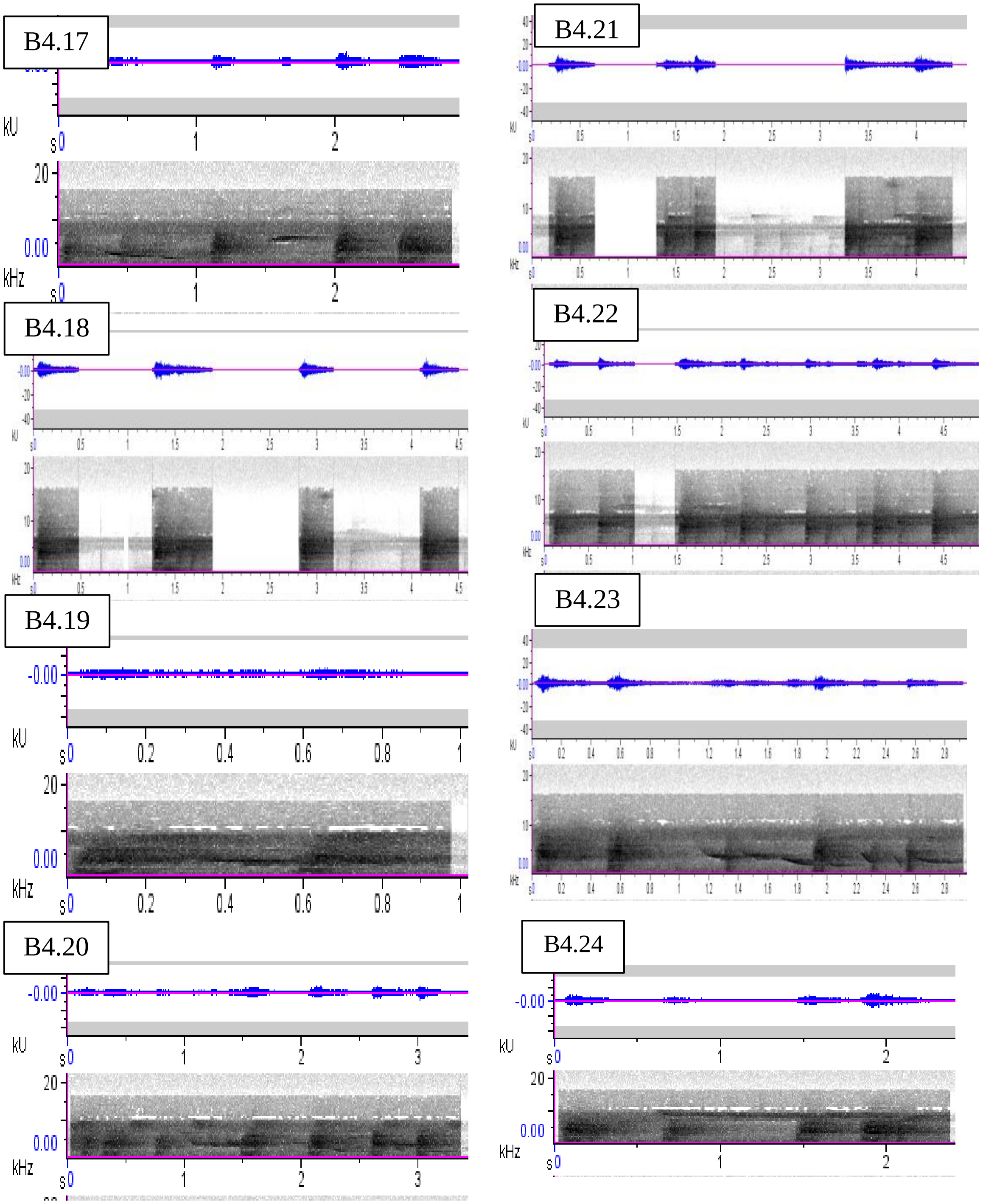

Appendix B.4. Continued…

B4.25

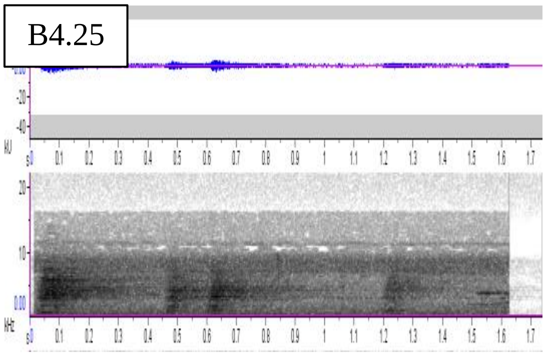

B4.26

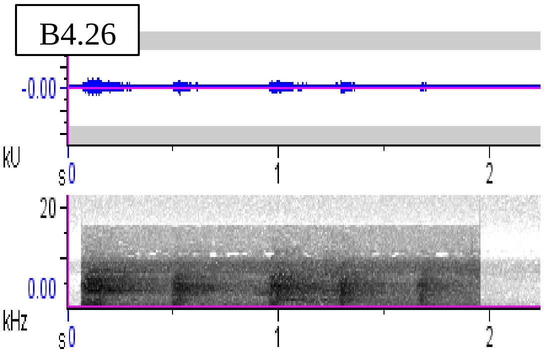

Appendix B.4. Continued…

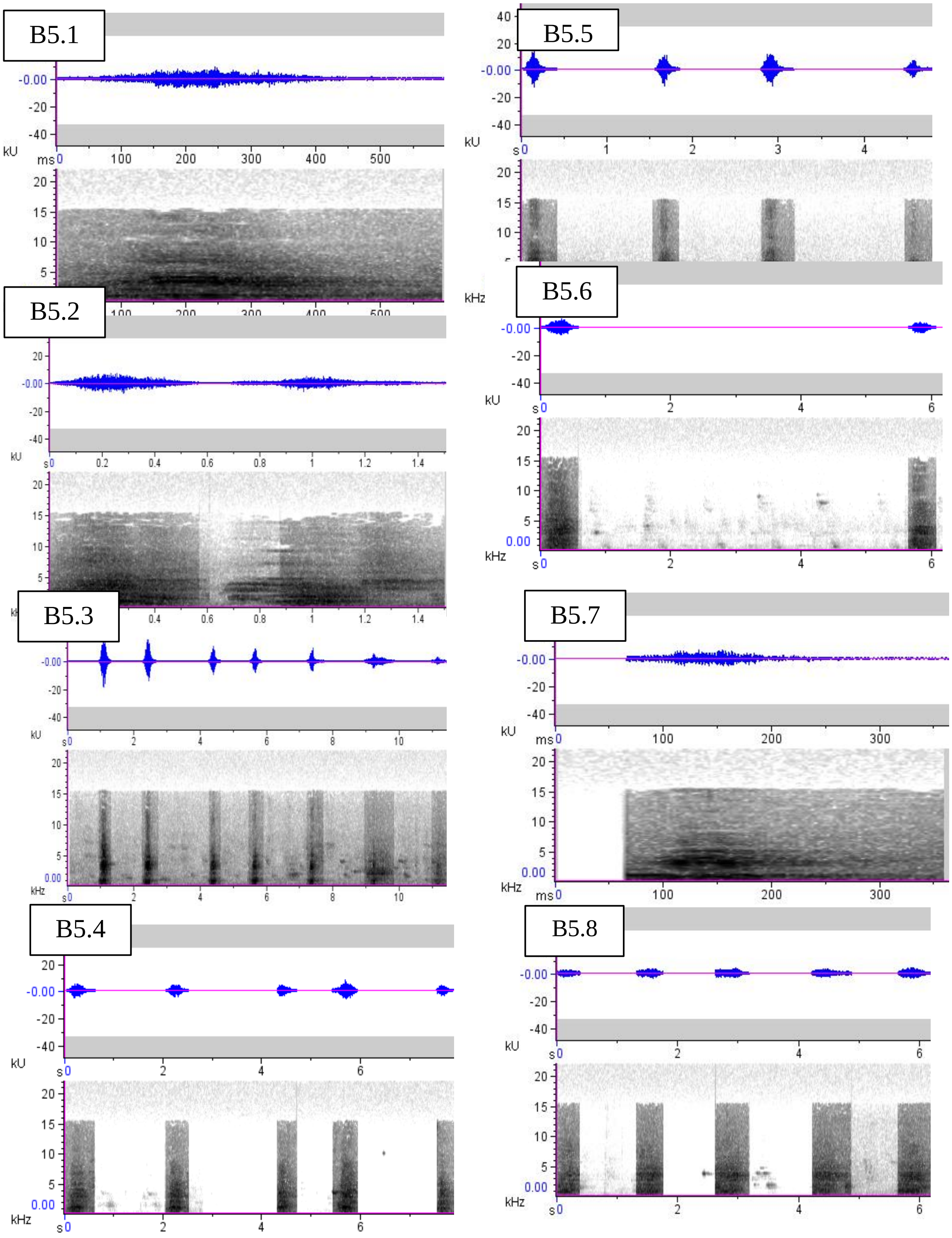

Appendix B5. Waveform and Spectrogram of *R. leucocephalus*.

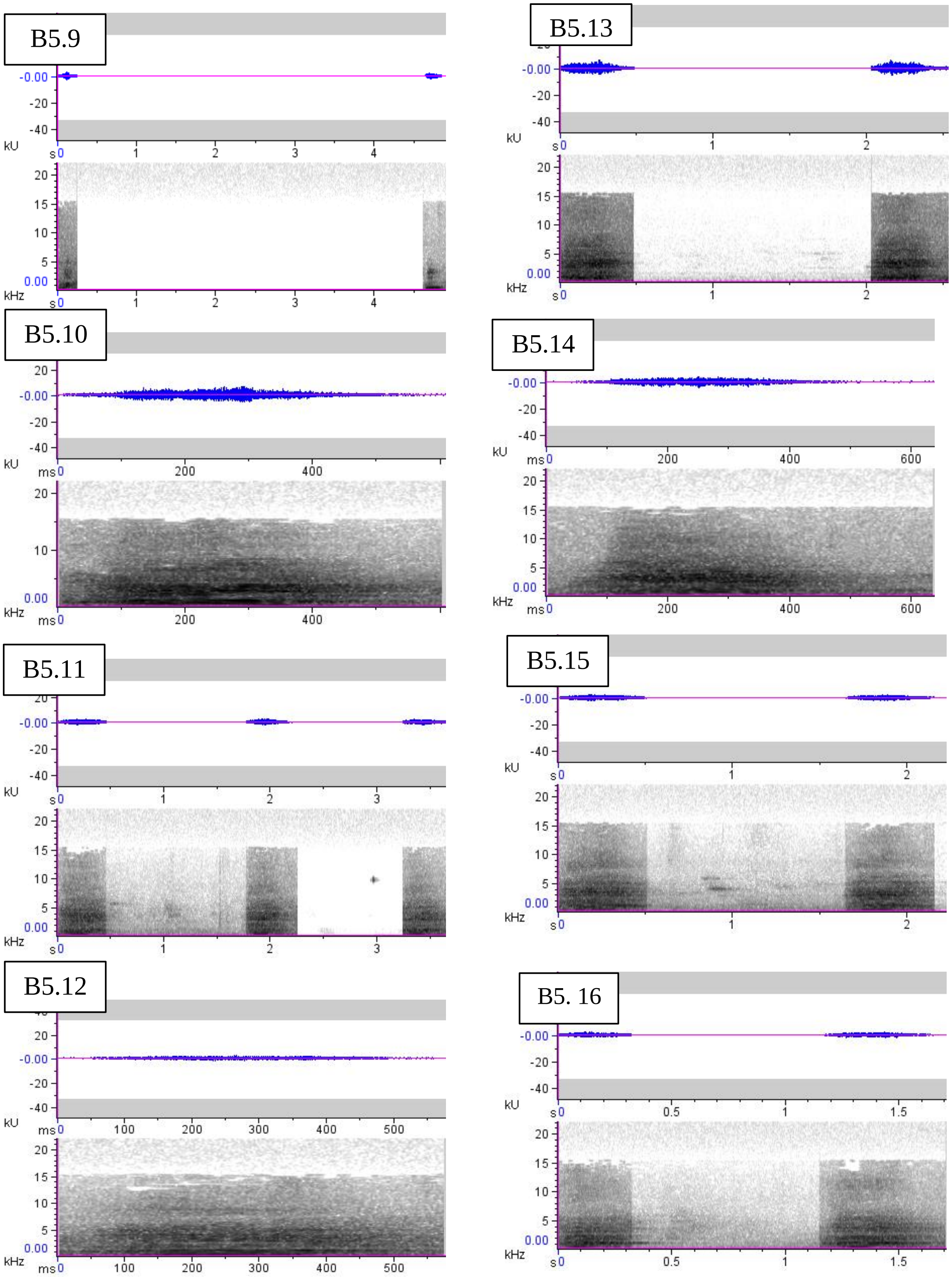

Appendix B.5. Continued…

B5.17

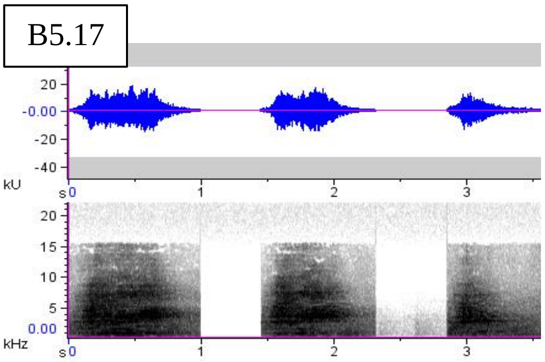

B5.18

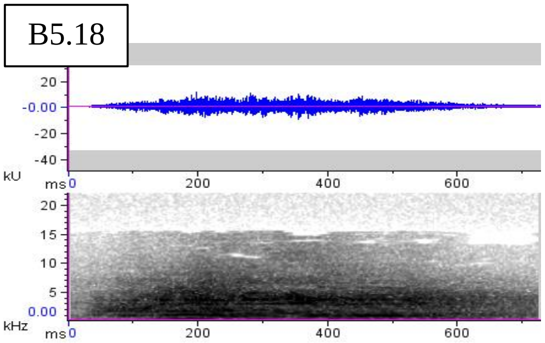

B5.19

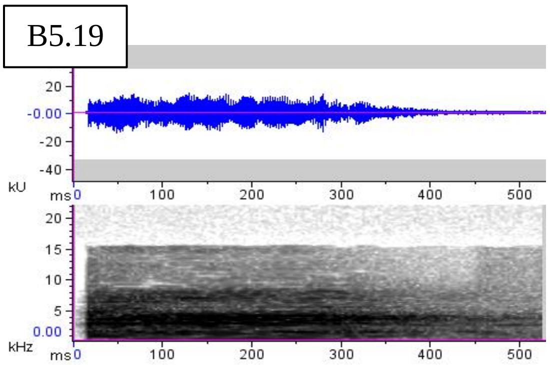

B5.20

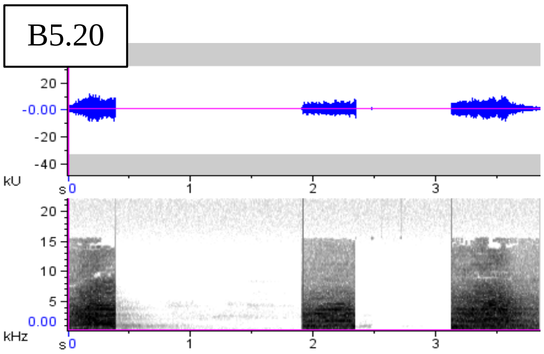

B5.21

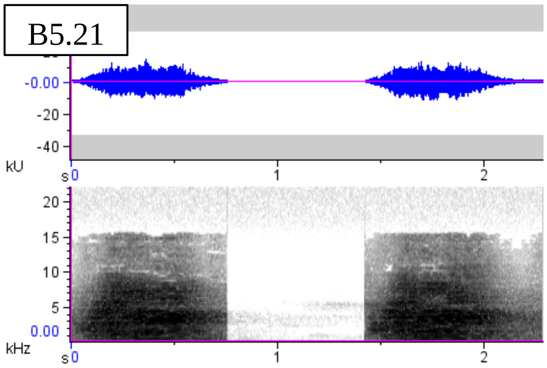

B5.22

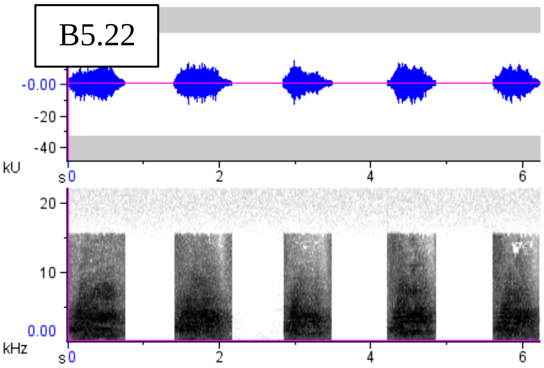

B5.23

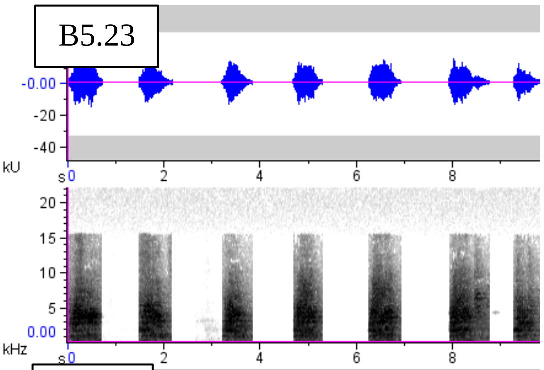

B5.24

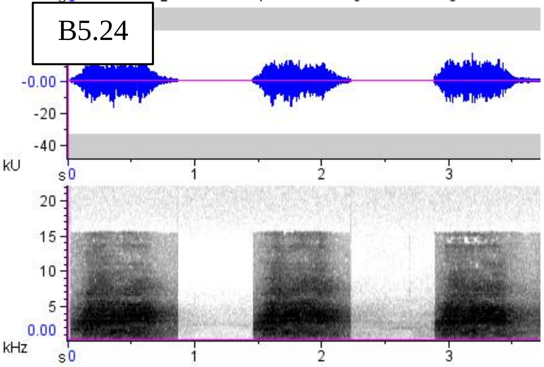

Appendix B.5. Continued…

B5.25

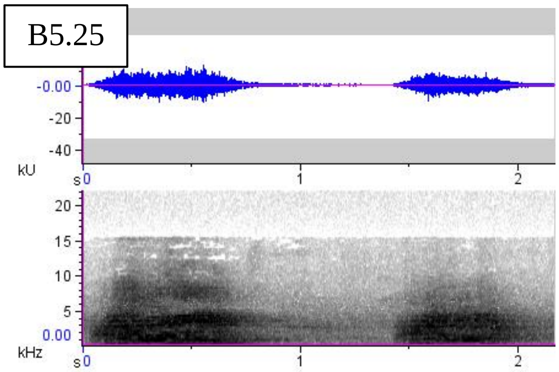

B5.26

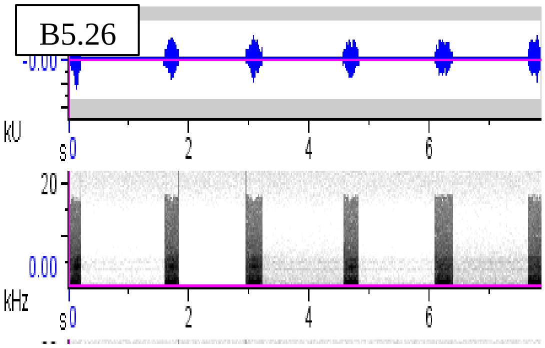

Appendix B.5. Continued…

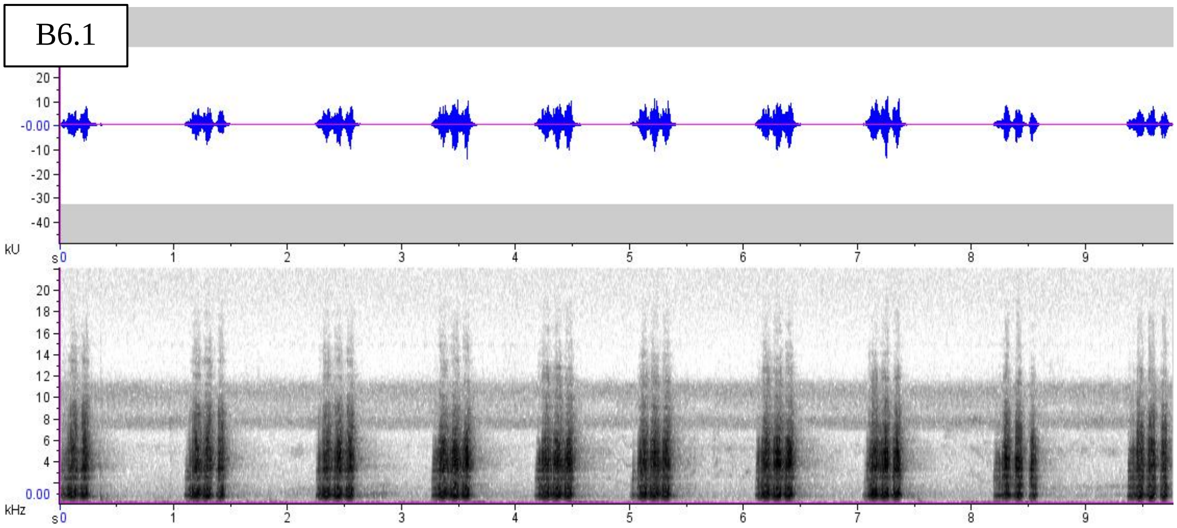

Appendix B.6. Waveform and Spectrogram of *R. waldeni*.

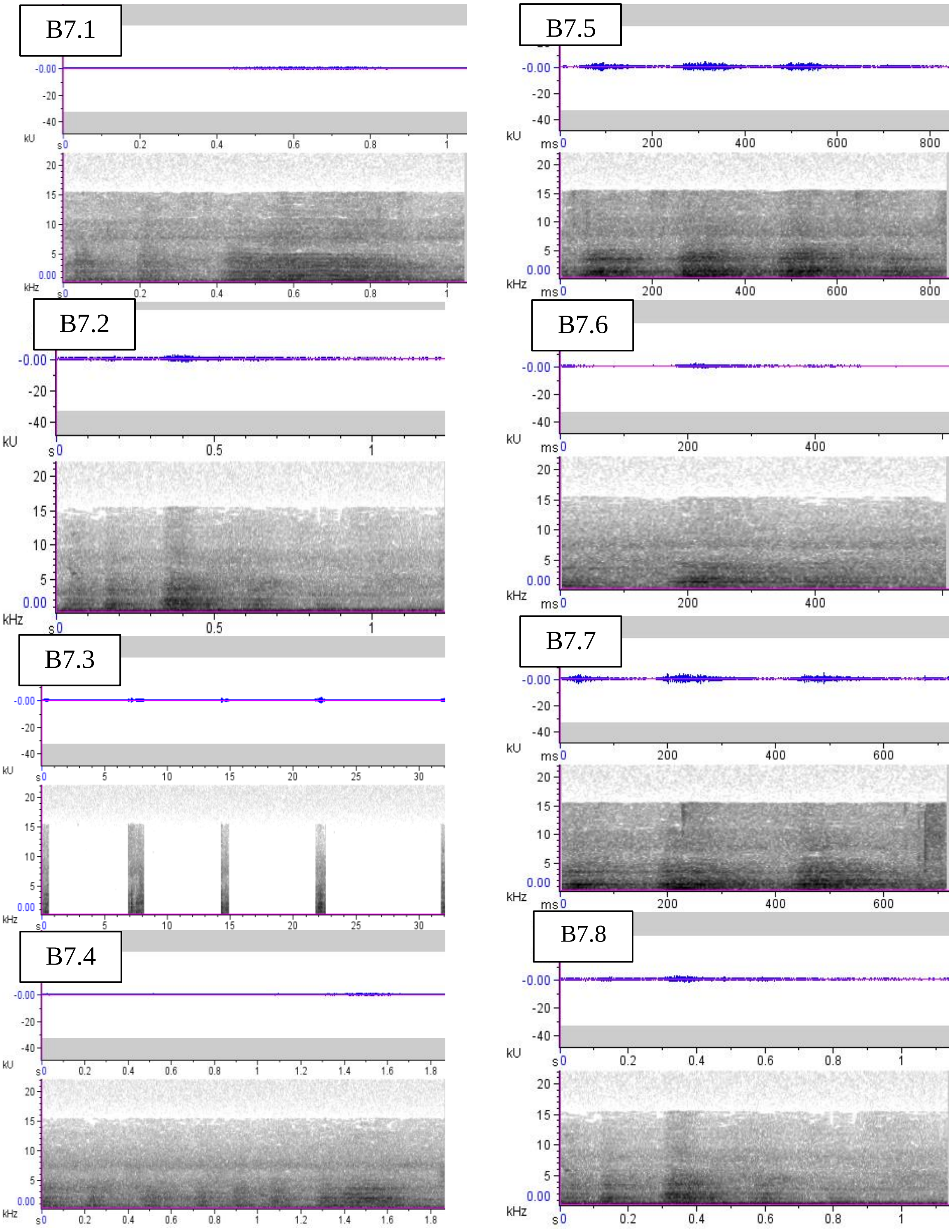

Appendix B.7. Waveform and Spectrogram of *R. plicatus*.

B7.9

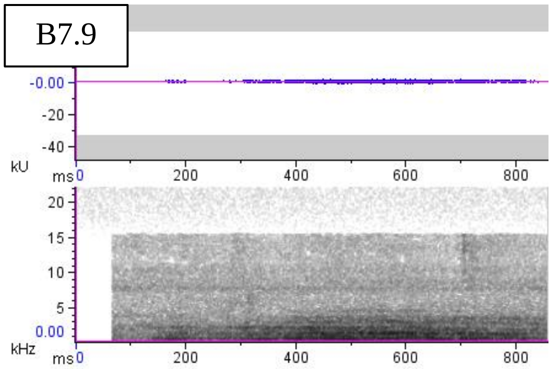

B7.11

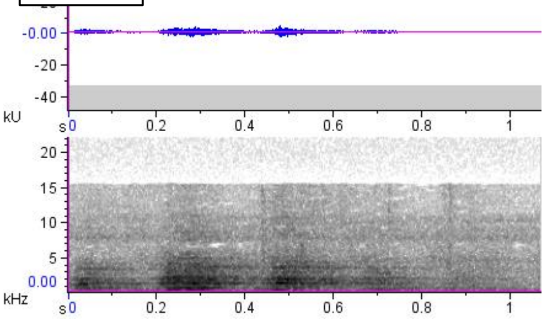

B7.10

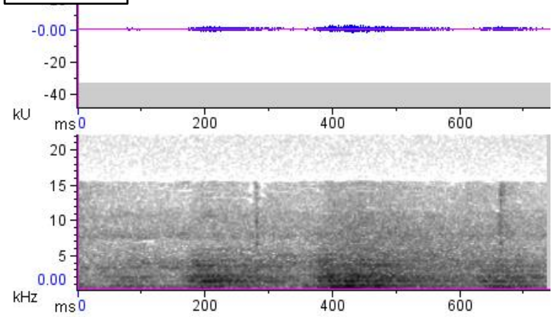

Appendix B.7. Continued…

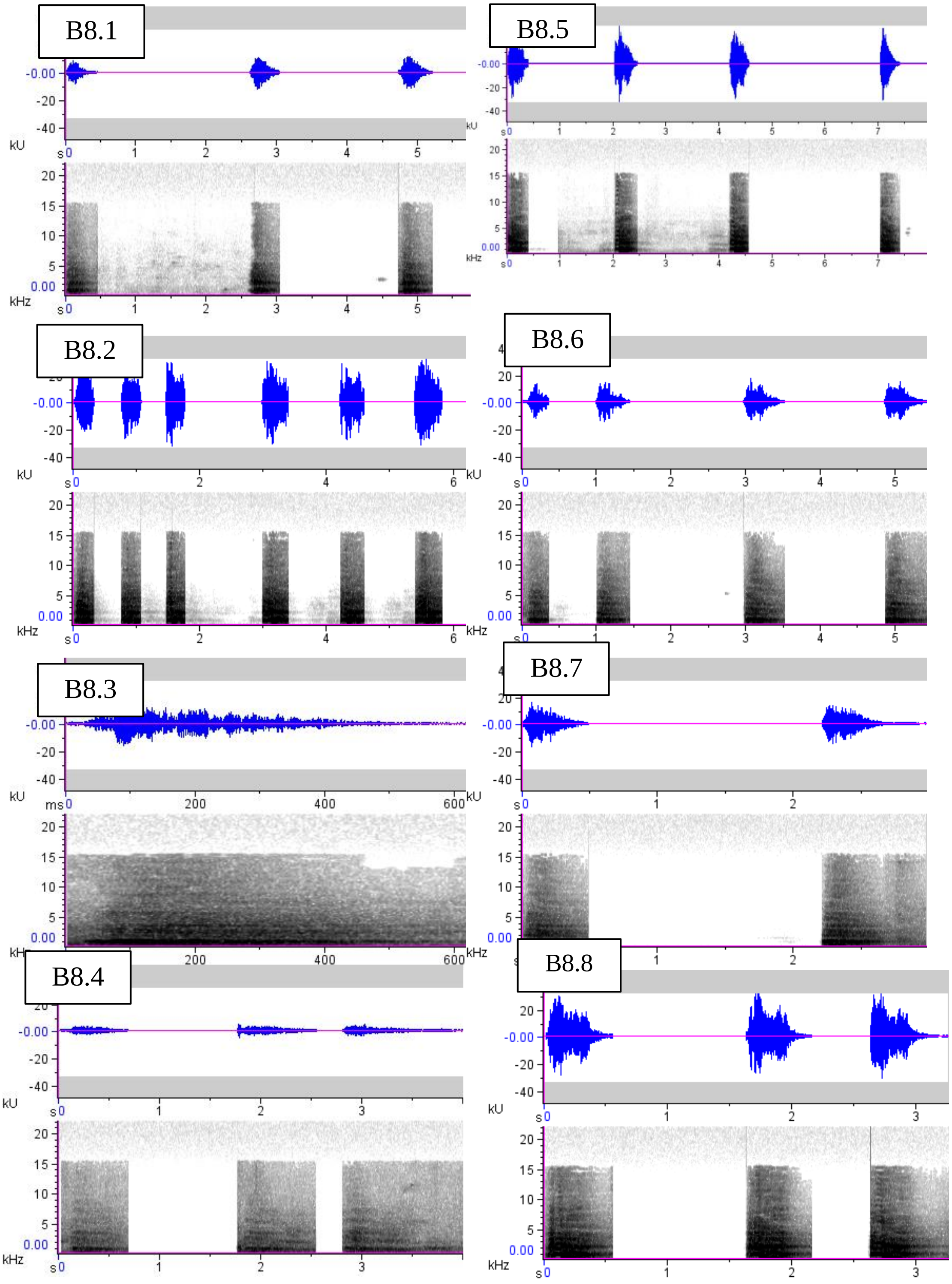

Appendix B.8. Waveform and Spectrogram of *B. h. hydrocorax.*

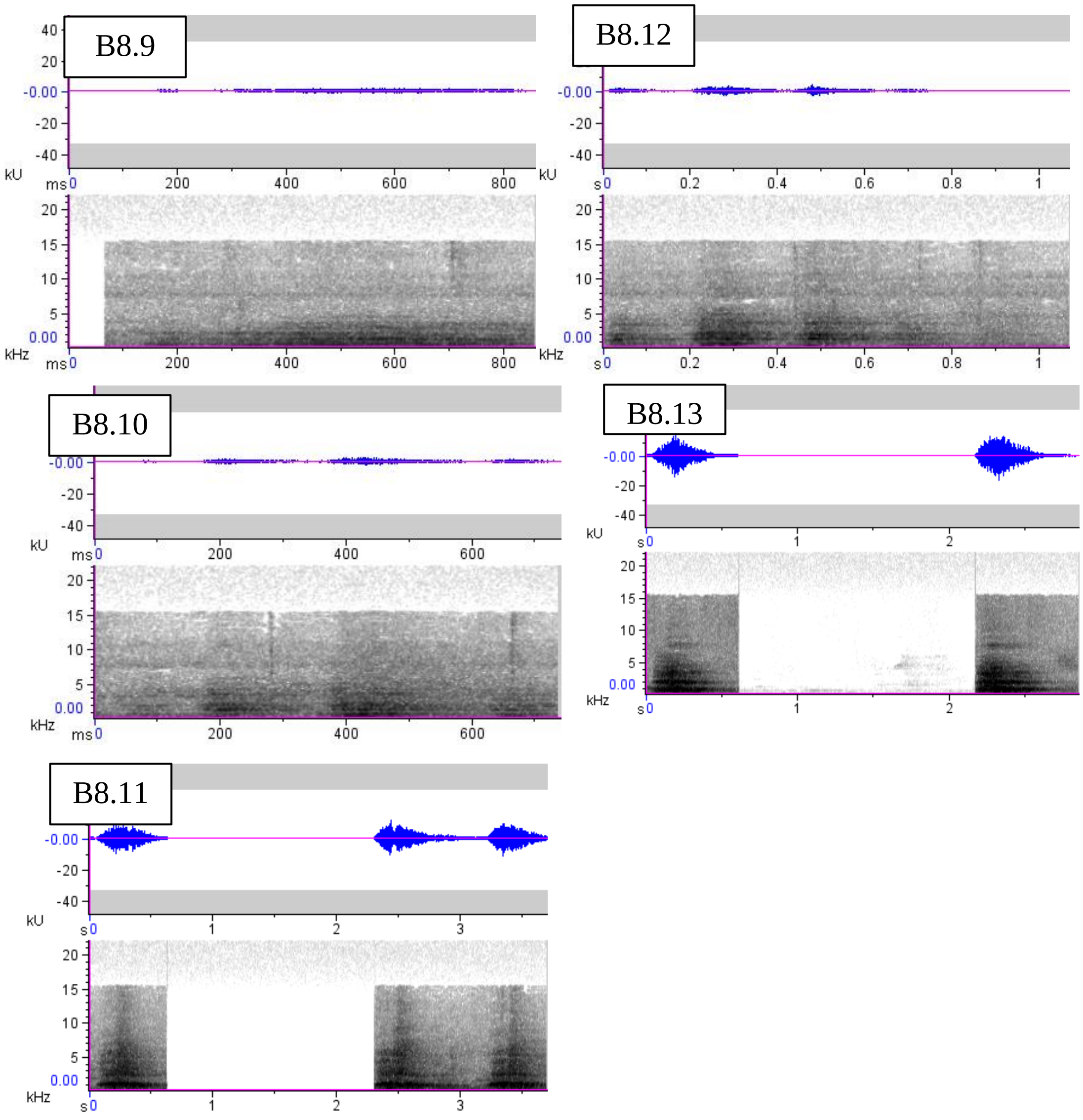

Appendix B.8. Continued…

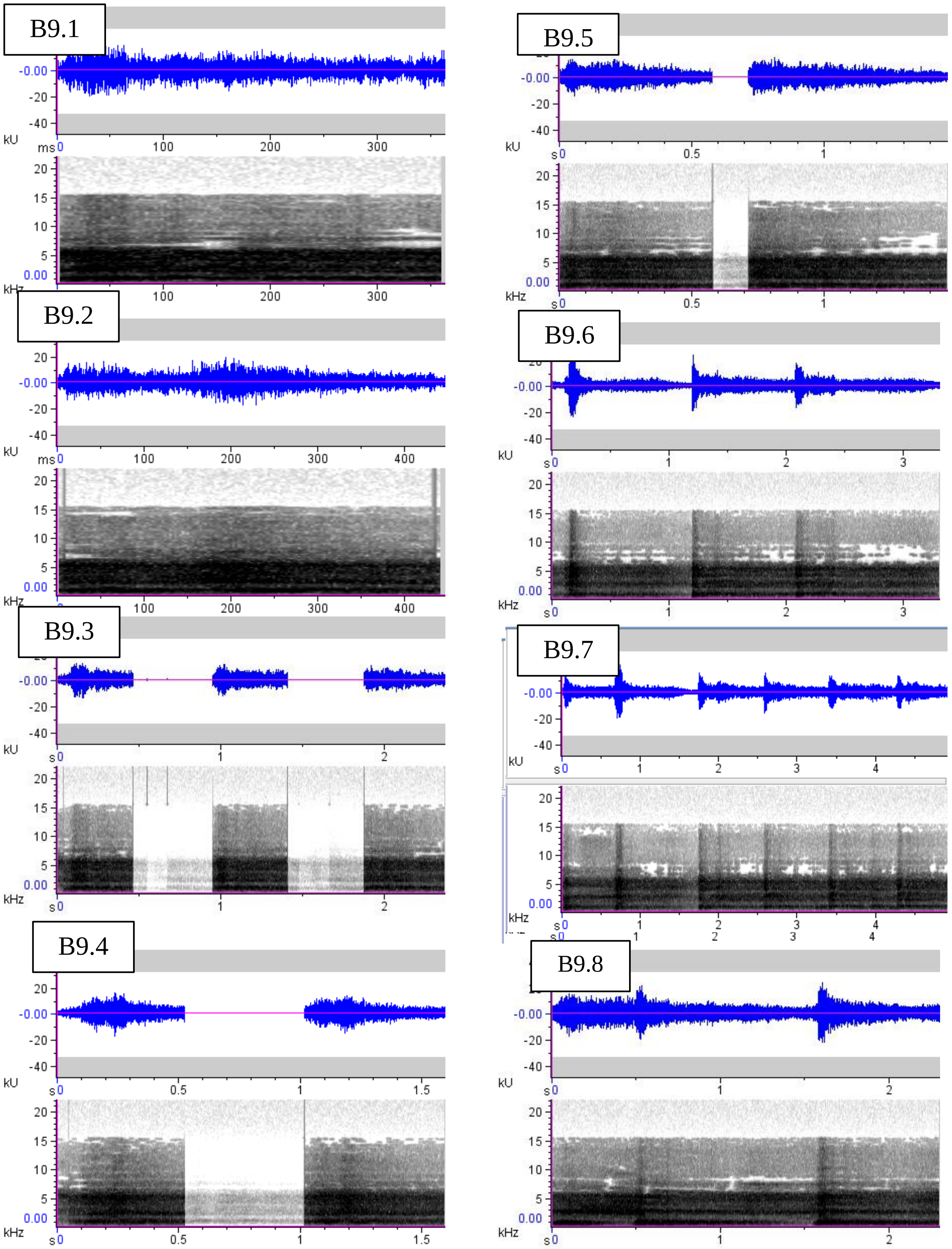

Appendix B.9. Waveform and Spectrogram of *B. h. semigaleatus*.

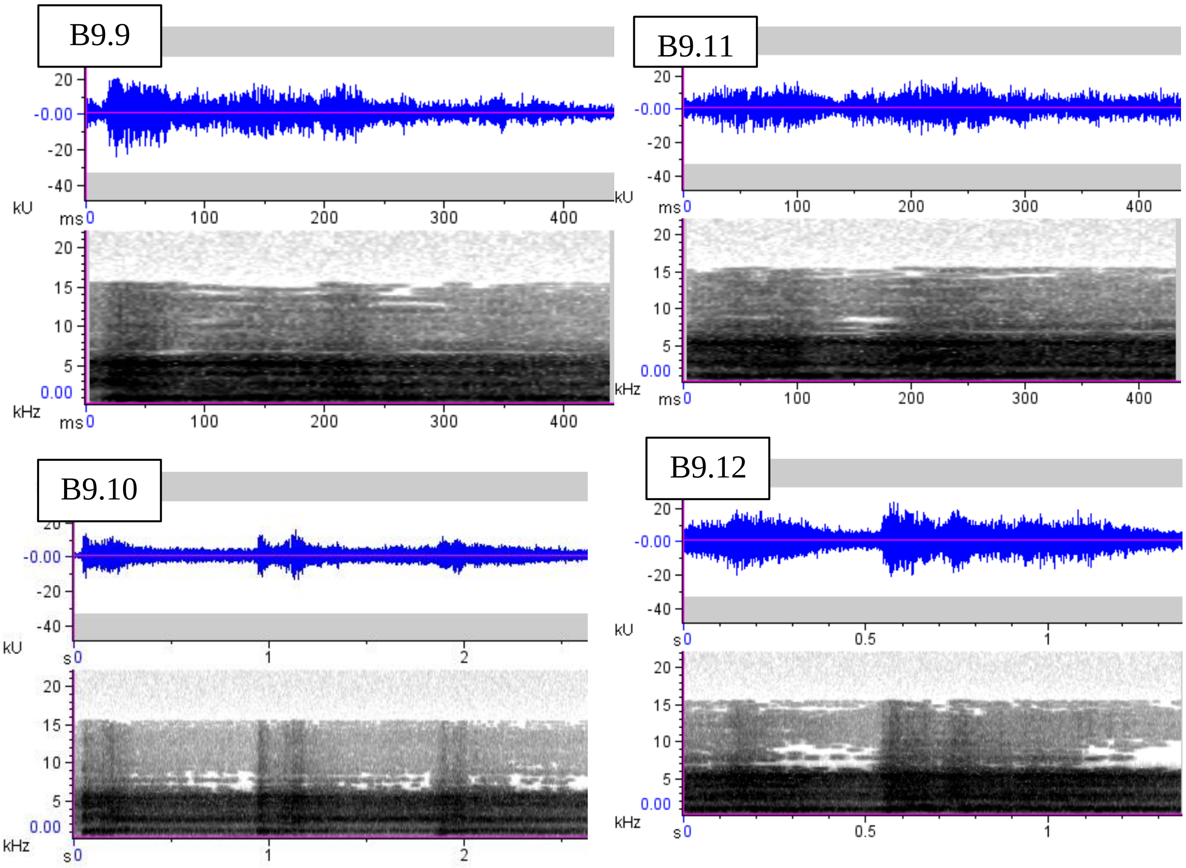

Appendix B.9. Continued…

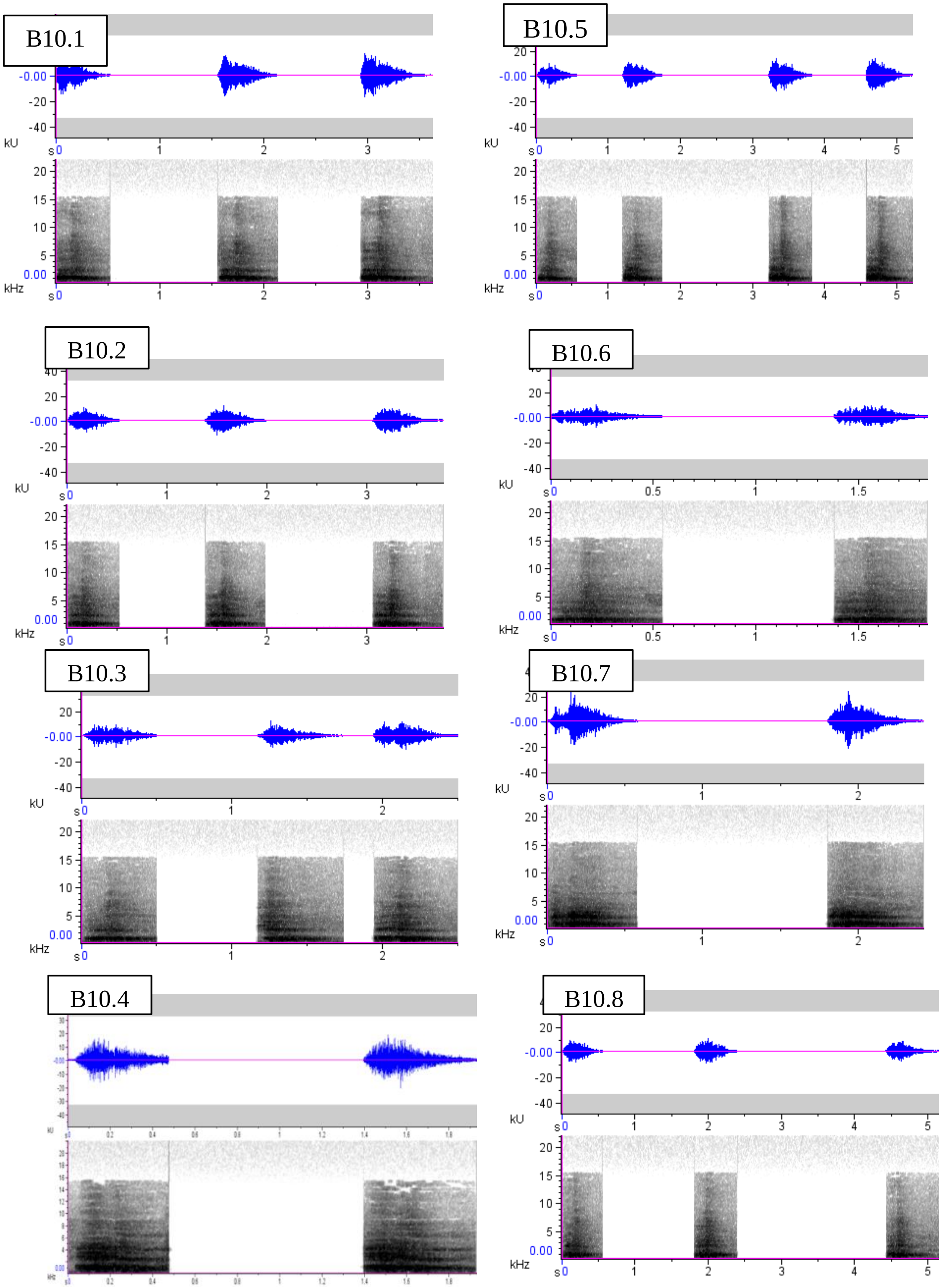

Appendix B.10. Waveform and Spectrogram of *B. h. mindanensis*.

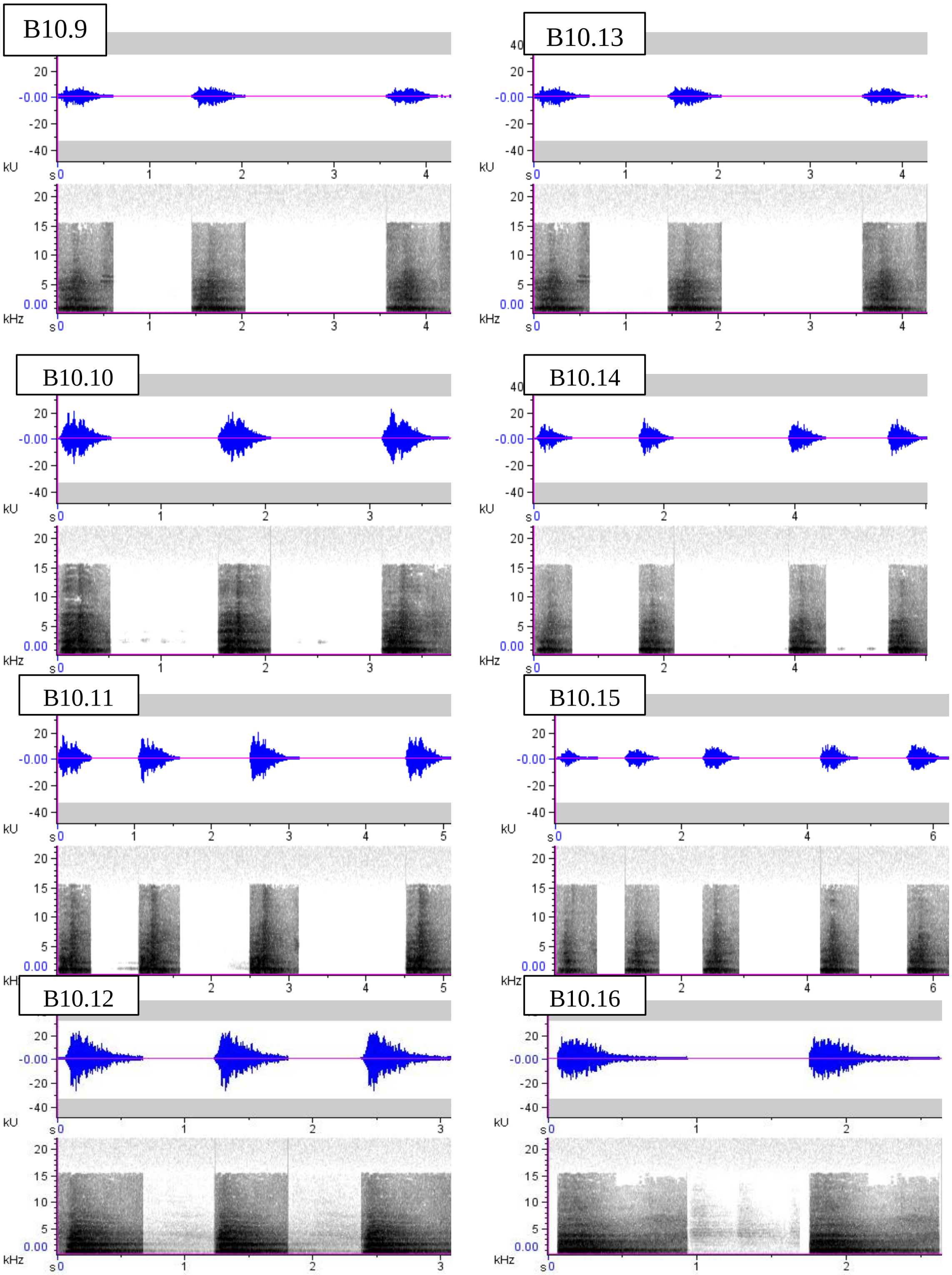

Appendix B.10. Continued…

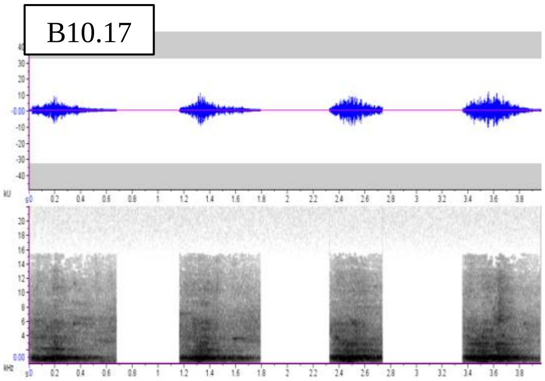

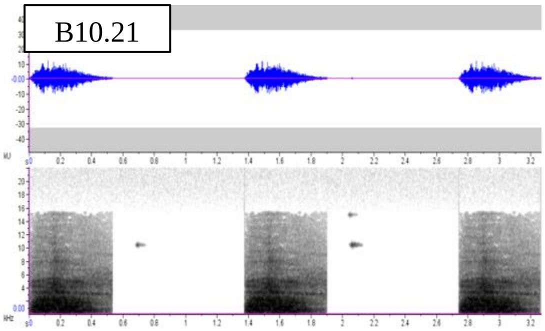

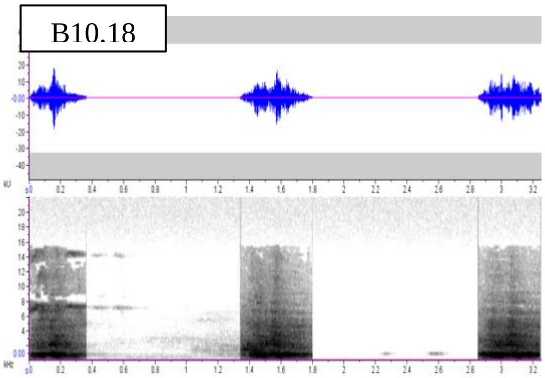

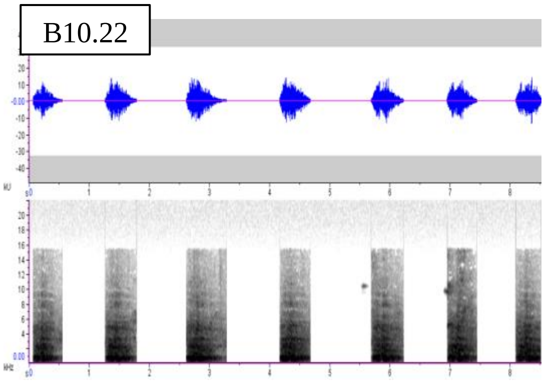

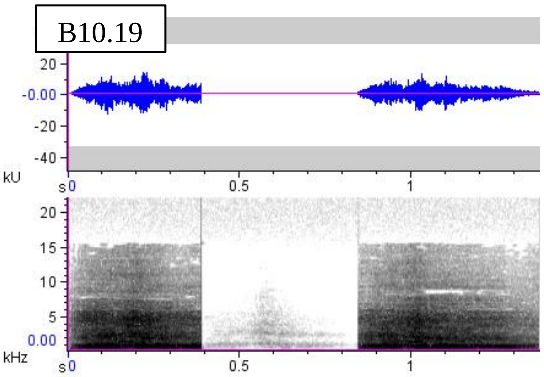

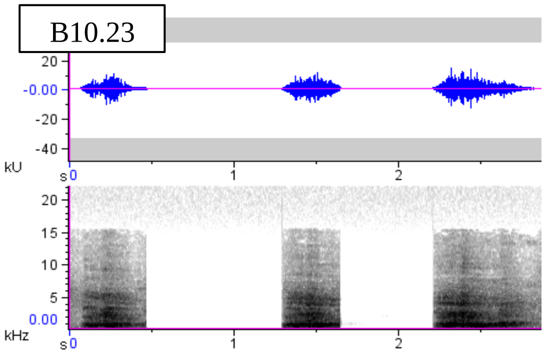

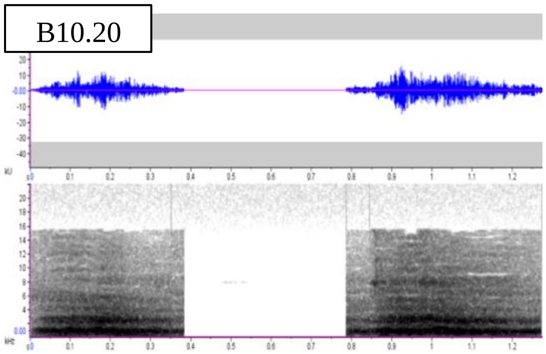

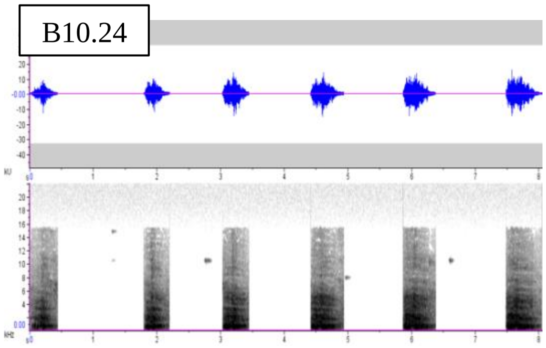

Appendix B.10. Continued…

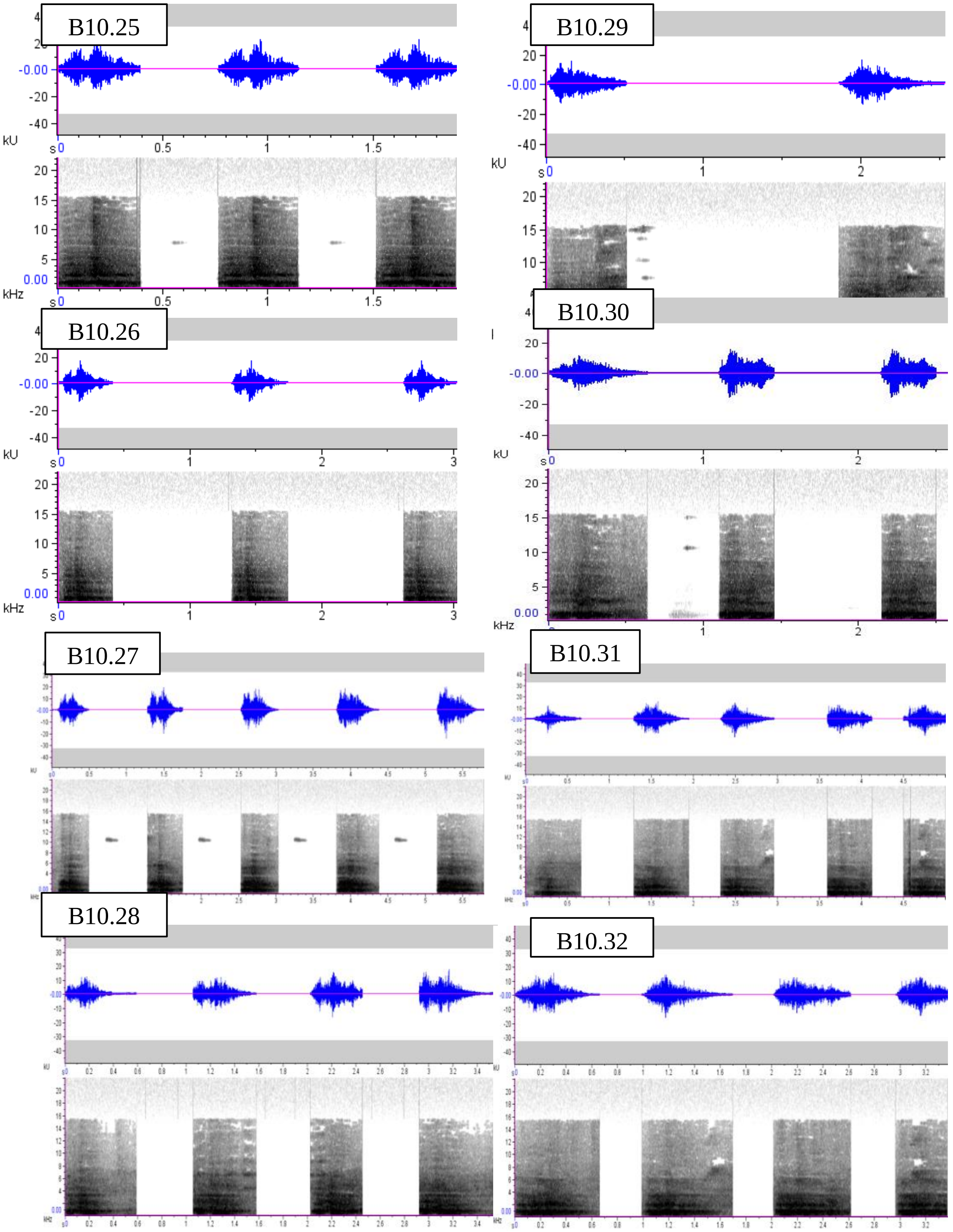

Appendix B.10. Continued…

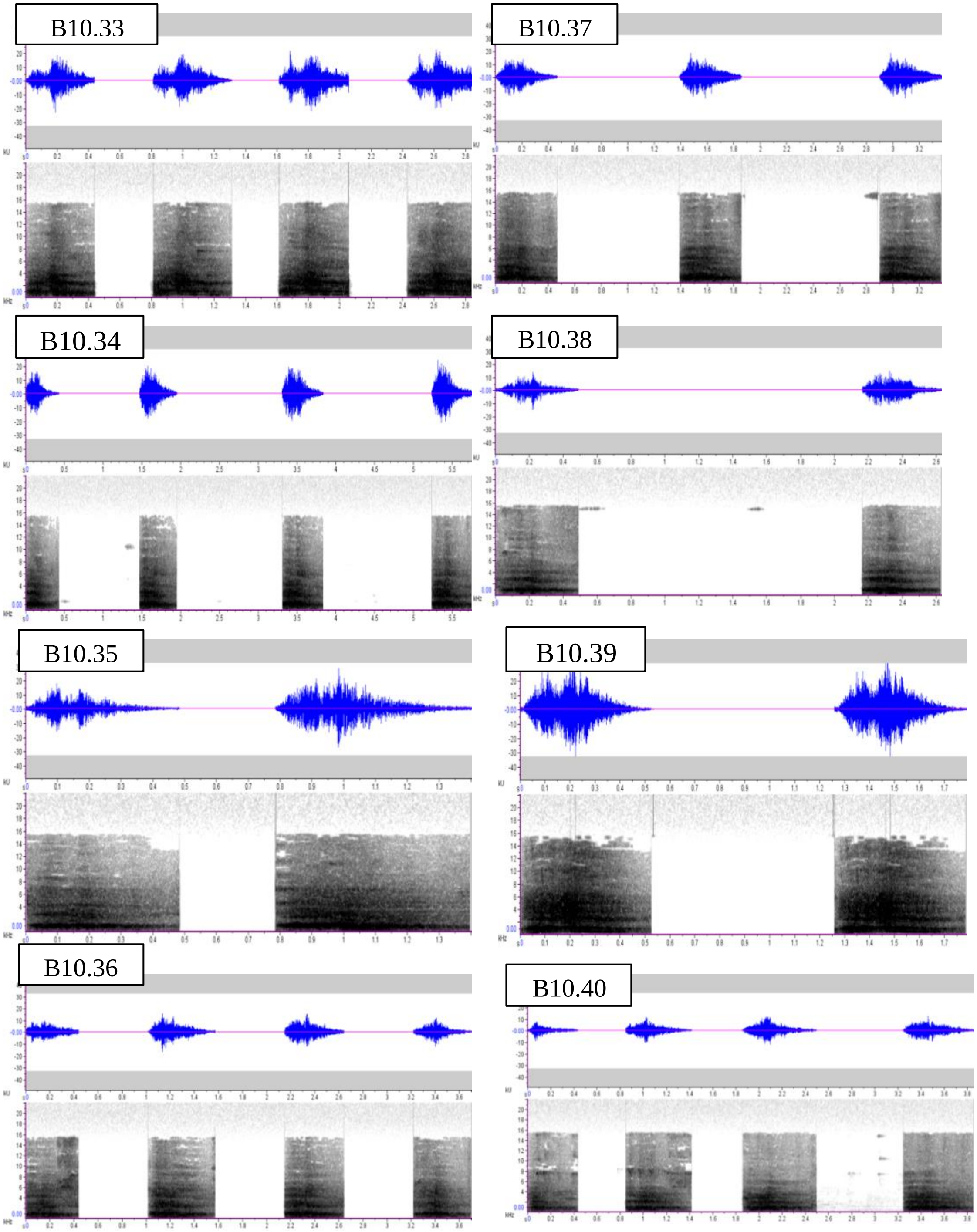

Appendix B.10. Continued…

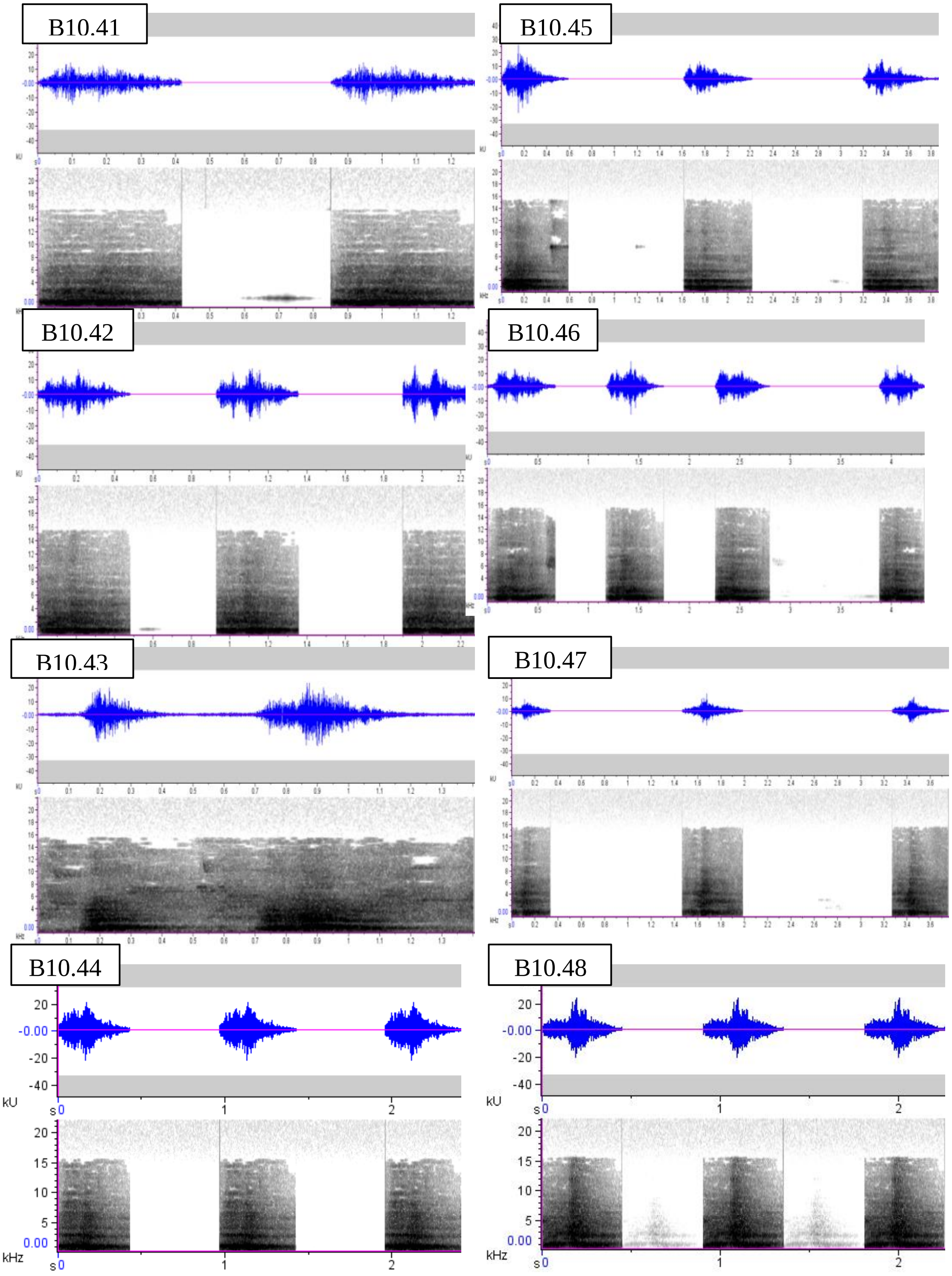

Appendix B.10. Continued…

B10.49

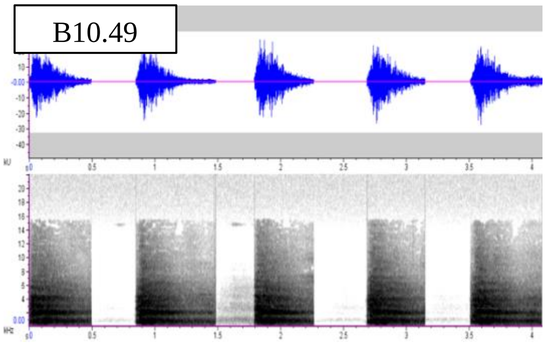

B10.51

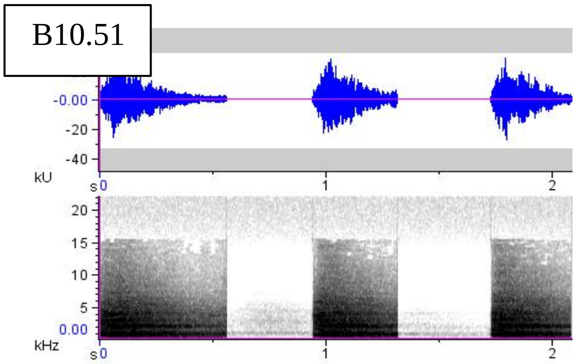

B10.50

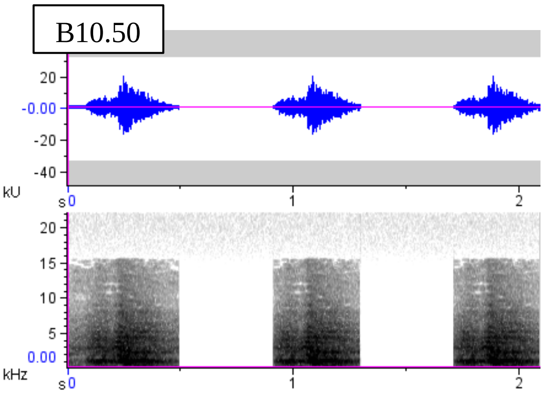

B10.52

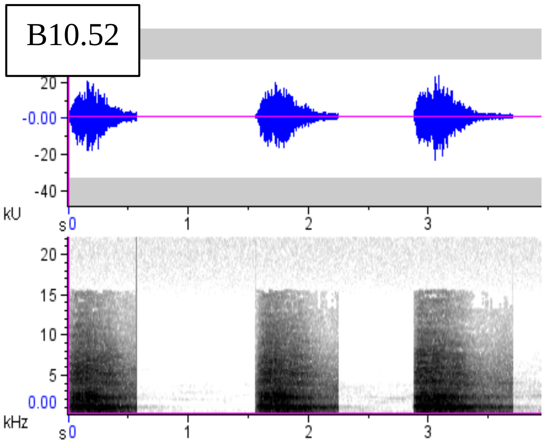

Appendix B.10. Continued…
